# Targeting CDK4 overcomes EMT-mediated tumor heterogeneity and therapeutic resistance in KRAS-mutant lung cancer

**DOI:** 10.1172/jci.insight.148392

**Published:** 2021-09-08

**Authors:** Aparna Padhye, Jessica M. Konen, B. Leticia Rodriguez, Jared J. Fradette, Joshua K. Ochieng, Lixia Diao, Jing Wang, Wei Lu, Luisa S. Solis, Harsh Batra, Maria G. Raso, Michael D. Peoples, Rosalba Minelli, Alessandro Carugo, Christopher A. Bristow, Don L. Gibbons

**Affiliations:** 1 Department of Thoracic/Head and Neck Medical Oncology, University of Texas MD Anderson Cancer Center, Houston, Texas, USA.; 2 University of Texas MD Anderson Cancer Center UTHealth Graduate School of Biomedical Sciences, Houston, Texas, USA.; 3 Department of Bioinformatics and Computational Biology,; 4 Department of Translational Molecular Pathology,; 5 TRACTION Platform, Division of Therapeutics Development, and; 6 Department of Molecular and Cellular Oncology, University of Texas MD Anderson Cancer Center, Houston, Texas, USA.

**Keywords:** Oncology, Lung cancer, Signal transduction

## Abstract

Lack of sustained response to therapeutic agents in patients with KRAS-mutant lung cancer poses a major challenge and arises partly due to intratumor heterogeneity that defines phenotypically distinct tumor subpopulations. To attain better therapeutic outcomes, it is important to understand the differential therapeutic sensitivities of tumor cell subsets. Epithelial-mesenchymal transition is a biological phenomenon that can alter the state of cells along a phenotypic spectrum and cause transcriptional rewiring to produce distinct tumor cell subpopulations. We utilized functional shRNA screens, in in vitro and in vivo models, to identify and validate an increased dependence of mesenchymal tumor cells on cyclin-dependent kinase 4 (CDK4) for survival, as well as a mechanism of resistance to MEK inhibitors. High zinc finger E-box binding homeobox 1 levels in mesenchymal tumor cells repressed p21, leading to perturbed CDK4 pathway activity. Increased dependence on CDK4 rendered mesenchymal cancer cells particularly vulnerable to selective CDK4 inhibitors. Coadministration of CDK4 and MEK inhibitors in heterogeneous tumors effectively targeted different tumor subpopulations, subverting the resistance to either single-agent treatment.

## Graphical abstract

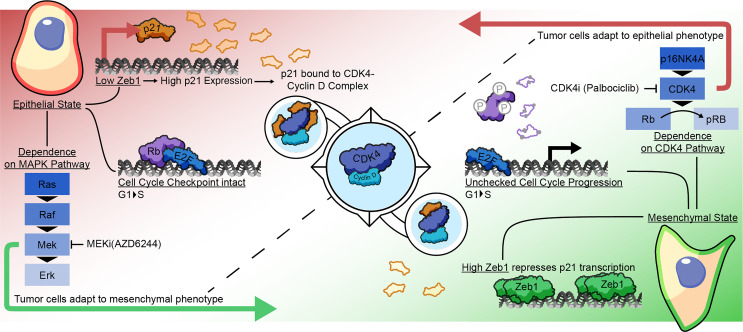

## Introduction

Activating *KRAS* mutation is one of the most frequent oncogenic events in lung cancer, occurring in about 30% of patients with lung adenocarcinoma (1–3). Despite the identification of the oncogene over 20 years ago and significant efforts to treat this subset of patients, 5-year survival rates remain dismal (4). Unlike *EGFR*-mutant lung cancer, KRAS oncoproteins are largely undruggable, with the very recent exception of the *KRAS^G12C^* allele (5, 6). Pharmacological inhibitors of the MAPK pathway (e.g., MEK), such as selumetinib and trametinib, are available, but preclinical and clinical trials have demonstrated poor responses to MEK inhibitors (7). Combination of MEK inhibitors with conventional chemotherapy did not demonstrate any added benefit to progression-free survival (8). Resistance to MEK inhibitors may be intrinsic (de novo) due to tumor cell heterogeneity or acquired due to tumor evolution as an adaptive response to pharmacological agents. In either case, the presence of phenotypically distinct tumor cell subpopulations with reprogrammed cellular machinery makes it difficult to effectively eliminate the broader tumor cell population. To address this, we need to understand the differences in the tumor cell subpopulations within a heterogeneous tumor.

Genetically identical tumor cells possess the ability to undergo transcriptional reprogramming to activate alternative survival pathways and evade therapeutic targeting. Research from our group and others has demonstrated that epithelial-mesenchymal transition (EMT) is a central phenomenon occurring in KRAS-mutant lung cancer, which contributes to intracellular tumor heterogeneity, increased metastatic potential, therapeutic resistance to pharmacological agents, and poor patient outcomes (9–11). Murine lung cancer models driven by Kras and p53 mutations recapitulate EMT-mediated tumor cell heterogeneity, with the zinc finger E-box binding homeobox 1/miRNA-200 (ZEB1/miR-200) double-negative feedback loop playing a central role in dynamically altering the cellular phenotype (10). Our previous research highlighted the reliance of KRAS-mutant epithelial-like lung cancer cells with high miR-200 expression on activated MAPK signaling pathway and increased susceptibility to MEK inhibitors. On the other hand, tumors demonstrating a mesenchymal-like phenotype due to elevated expression of ZEB1 remained largely unresponsive to MEK inhibitors. Moreover, after an initial response to MEK inhibition, tumors demonstrating epithelial phenotype acquired therapeutic resistance by undergoing EMT (11, 12). The study identified an unmet need to develop therapeutic approaches to target distinct tumor subpopulations within heterogeneous KRAS-mutant lung tumor to achieve a robust therapeutic response. Utilizing multiple loss-of-function shRNA screens, we analyzed the effects on phenotypically different tumor subpopulations and identified cyclin-dependent kinase 4/retinoblastoma protein (CDK4/RB) as a major survival pathway in mesenchymal cell–like tumor cells.

CDK4 acts as a master integrator of mitogenic/oncogenic signaling cascades by inactivating the central tumor suppressor RB and cell cycle commitment at the restriction point allowing cells to transition to S phase (13). The CDK4 axis is altered in many cancers, with clinically approved pharmacological inhibitors showing promising antitumor activity (14). Some studies have shown that CDK4 and cyclin D1 expression is correlated with the presence of *KRAS* mutation in lung tumors (15), and a synthetic lethal interaction occurs between KRAS and CDK4 in lung cancer tumor progression (16, 17). We found that the differential activation of the CDK4 pathway in epithelial-like and mesenchymal-like cells was determined by ZEB1-mediated p21 regulation. Levels of p21, an intrinsic regulator of CDK4, in cells determine the downstream CDK4 pathway activity. p21 is transcriptionally regulated by direct binding of the transcription factor ZEB1 to the promoter region. Our study demonstrates in multiple preclinical models that intrinsic and acquired MEK inhibitor resistance is associated with a rewired kinome in tumors by which the mesenchymal phenotype activates the CDK4 pathway as a common occurrence across models. This dependence on the CDK4 pathway resulted in a potential therapeutic approach to combine MEK and CDK4 inhibitors to target different tumor subpopulations along the EMT spectrum and combat resistant outgrowth of epigenetic subsets in a heterogeneous tumor.

## Results

### Mesenchymal lung cancer cells exhibit increased dependency on CDK4 for growth.

**Figure 1:**
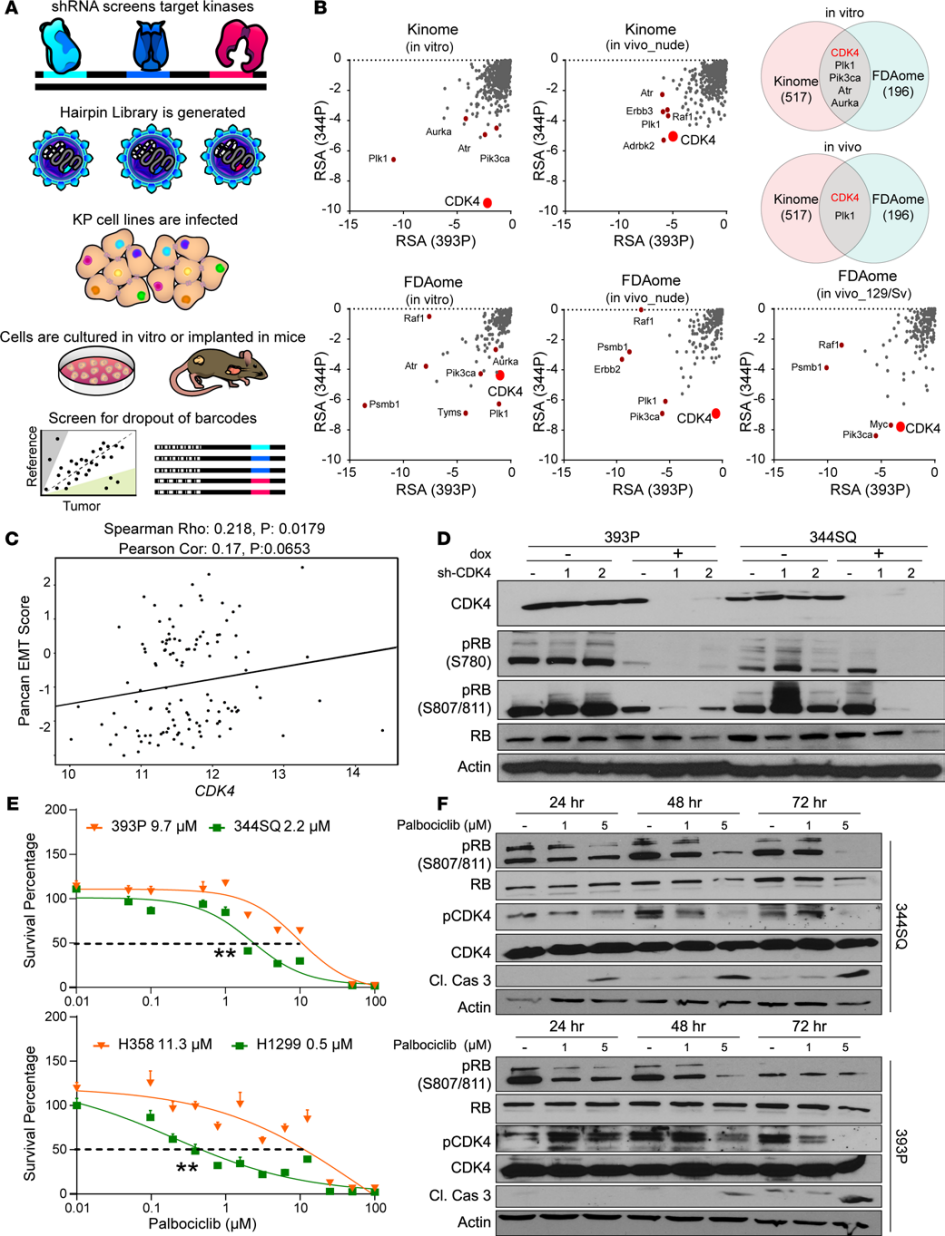
Mesenchymal lung cancer cells exhibit increased dependency on CDK4 for growth. (**A**) Schematic illustration of the workflow of the shRNA dropout screens. A library of lentiviral particles expressing 10 different barcoded shRNAs was transduced into murine KP-mutant lung cancer cells. The cells were cultured in vitro or implanted in nude or syngeneic 129/Sv mice and later sequenced for barcoded shRNAs and compared with reference cells. (**B**) Results from Kinome and FDAome shRNA dropout screens in 393P and 344P cell lines and tumors compared based on the redundant shRNA activity (RSA). Top differential hits are labeled on the graphs, most important being CDK4. Venn diagram shows comparisons across different conditions and top hits identified. (**C**) Cluster plot analysis of correlation between EMT score and *CDK4* mRNA expression of 118 human NSCLC cell lines. (**D**) Western blot analysis of CDK4 pathway after 6 days of CDK4 knockdown. (**E**) In vitro cell viability after 48-hour palbociclib treatment in a panel of epithelial and mesenchymal murine (393P, 344SQ) and human (H358, H1299) lung cancer cell lines. *n* = 8 per drug concentration. The curve was generated using a nonlinear regression fit model. Vertical error bars shown. **P < 0.001, 2-tailed Student’s *t* test. (**F**) 344SQ and 393P cells were treated for 24, 48, and 72 hours with 1 and 5 μM palbociclib, and Western blot analysis was utilized to demonstrate drug efficacy over a dose range. Cleaved caspase-3 was used as an apoptotic marker.

In order to effectively target the mesenchymal tumor subpopulations within heterogeneous tumors, we sought to identify the survival dependencies of these tumor cells. A loss-of-function screen with a barcoded, pooled small hairpin RNA (shRNA) library targeting about 500 genes with known kinase activity (Kinome) was conducted. Each gene was targeted with 10 unique shRNA sequences to limit false hits due to off-target effects. This library of shRNAs was transduced into representative nonmetastatic, epithelial-like (393P) and metastatic, mesenchymal-like (344P) murine lung cancer cell lines derived from a previously described *Kras/p53*-mutant (KP) genetically engineered mouse model (10). The cell lines stably expressing the shRNAs from the Kinome library were either cultured in vitro or implanted subcutaneously in nude mice (Figure 1A). Tumors were harvested, shRNA barcodes were quantified by deep sequencing and referenced with the respective in vitro cell population, and quality control measures were completed to ensure sufficient barcode coverage across the library was maintained in vivo (Supplemental Figure 1A and Supplemental Table 1; supplemental material available online with this article; https://doi.org/10.1172/jci.insight.148392DS1). The phenotypic impact of gene knockdown was inferred by the redundant shRNA activity (RSA) algorithm, where a lower rank of the shRNA barcodes signified dropout from the population and greater dependency on the gene for tumorigenesis (Supplemental Table 2). Although both cell line models have activating *Kras^G12D^* and *p53^R172H^* mutations, comparison of the results of the Kinome screen revealed that the mesenchymal-like cells (344P) and the matched syngeneic tumors were more reliant on *Cdk4* for in vitro and in vivo growth (Figure 1B). We also compared these results to our previously published FDAome shRNA screen (11) and identified *Cdk4* as the most consistent hit across in vitro and in vivo conditions in both screens (Figure 1B, Supplemental Figure 1A, and Supplemental Tables 3 and 4). *CDK4* mRNA expression showed a positive correlation with a previously reported 76-gene EMT signature (18) in 118 human non–small cell lung cancer (NSCLC) cell lines (Figure 1C). When subclassified based on mutational status, 41 KRAS-mutant NSCLC cell lines also showed positive correlation of *CDK4* mRNA with the EMT signature (Supplemental Figure 1B). A panel of epithelial cell–like and mesenchymal-like murine lung cancer cells were tested, and the mesenchymal-like cells demonstrated higher *Cdk4* mRNA levels (Supplemental Figure 1C). We employed a genetic approach to confirm the dependency of mesenchymal cell–like tumor cells on CDK4 for survival. Mesenchymal-like cells with an inducible shRNA targeting CDK4 showed a greater reduction in tumor cell growth (Supplemental Figure 1, D and E), with suppression of phosphorylated RB (Figure 1D) compared with epithelial-like 393P cells. In fact, 393P tumor cells appeared to have slightly greater growth rate with CDK4 knockdown than the control cells (Supplemental Figure 1E) and continued RB phosphorylation (Figure 1D). We did not observe a significant difference in baseline proliferation rate between 393P and 344SQ murine lung cancer cells (Supplemental Figure 1F).

Next, we functionally validated the shRNA screen and determined whether response to CDK4 inhibitors is dependent on the EMT status of tumor cells. We treated a panel of human and murine lung cancer cells, stratified as epithelial-like or mesenchymal-like based on previous profiling (10, 18), with CDK4 inhibitors. Both human and murine mesenchymal-like lung cancer were more sensitive to CDK4 inhibitors (palbociclib, abemaciclib, and ribociclib) (Figure 1E and Table 1). As previously noted (10), EMT status is tightly regulated by the ZEB1/miR-200 double-negative feedback loop, and manipulation of this axis can induce an epithelial or mesenchymal shift in tumor cells. We therefore utilized isogenic pairs of human (H441) and murine (393P) epithelial-like cell lines with ZEB1 expression to produce a mesenchymal phenotype (19) and isogenic pairs of human (H1299, H23) and murine (344SQ) mesenchymal-like cells with miR-200 expression or ZEB1 knockdown to push the cells to an epithelial state (20). Comparisons across the different cell line pairs revealed that sensitivity to the CDK4 inhibitors was determined by the EMT status (Supplemental Figure 2, A–C, and Table 1).

The downstream targets of CDK4, RB and FoxM1, are important readouts for CDK4 kinase activity, whereas phosphorylated CDK4 may continue to be present for another 24 hours after inhibitor treatment. Suppression of RB and FoxM1 was observed in mesenchymal-like cells upon treatment with abemaciclib or ribociclib for 24 and 48 hours (Supplemental Figure 2D). Initially, 393P cells showed suppression of CDK4 targets, but it was not a sustained response. 344SQ cells showed a more robust response to the inhibitor palbociclib over a range of concentrations and at shorter treatment times compared with 393P in terms of suppression of downstream signaling and induction of apoptosis (Figure 1F).

### The CDK4 pathway is dynamically regulated by the EMT status of tumor cells.

**Figure 2:**
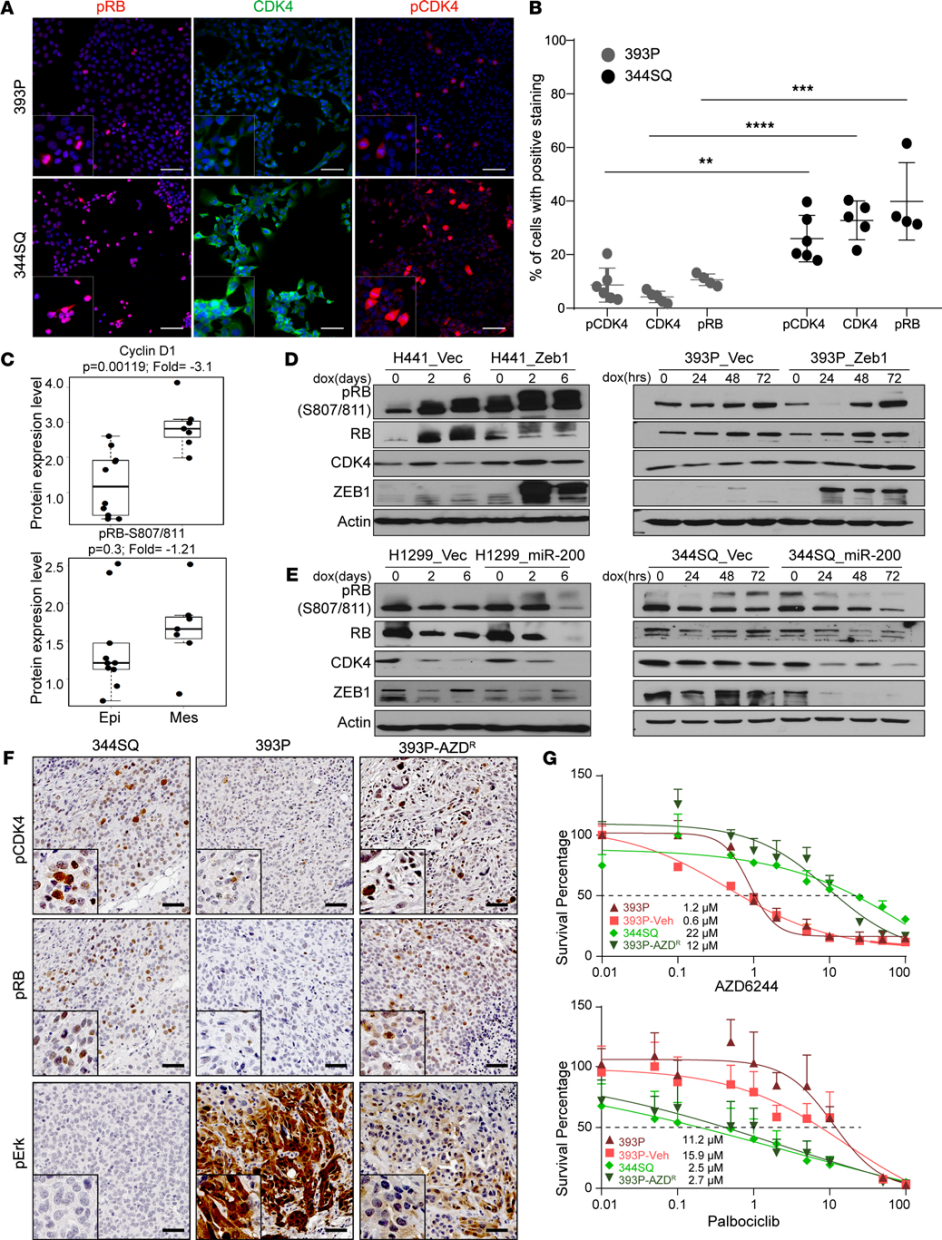
The CDK4 pathway is dynamically regulated by the EMT status of tumor cells. (**A**) Representative images of immunofluorescence on 393P and 344SQ cells for indicated markers. Scale bar: 50 μm. (**B**) Quantification of the fluorescent signal in 4–6 biological replicates. Data are represented as mean ± SD. One-way ANOVA was used for statistical analysis. (**C**) Dot plots of Cyclin D1 and RB phosphorylation (S807/811) from RPPA data set in a panel of epithelial and mesenchymal murine lung cancer cell lines. The box plots depict the minimum and maximum values (whiskers), the upper and lower quartiles, and the median. The length of the box represents the interquartile range. (**D** and **E**) Western blot analysis of CDK4 pathway in human and murine cells with ZEB1 or miR-200 overexpression. Cells were induced with 2 μg/mL of doxycycline for times indicated. (**F**) IHC on tumors derived from 393P, 344SQ, and 393P_AZD^R^ tumors for indicated markers. Scale bar: 50 μm. (**G**) In vitro cell viability assay on 393P, 344SQ, 393P_vehicle, and 393P_AZD^R^ after 48 hours of AZD6244 and palbociclib treatment. *n* = 8 per drug concentration. The curve was generated using a nonlinear regression fit model. Vertical error bars shown. ****P < 0.0001; ***P < 0.005; **P < 0.001, 2-tailed Student’s *t* test.

Immunofluorescence staining of tumor cells demonstrated an activated CDK4/RB axis with a higher percentage of 344SQ tumor cells with positive nuclear staining for phosphorylated CDK4 (phospho-CDK4) and phospho-RB and a stronger staining for total CDK4 (Figure 2, A and B). Using reverse phase protein arrays (RPPAs) to analyze changes in cell signaling proteins in a high-throughput manner, we screened a panel of previously characterized isogenic murine epithelial-like and mesenchymal-like lung cancer cell lines and observed an increase in CDK4 axis–related molecules, phospho-RB and Cyclin D1, in cells with a mesenchymal phenotype (Figure 2C). A subcellular fractionation assay also showed higher levels of phospho-RB, Cyclin D1, and CDK4 in mesenchymal-like cells (Supplemental Figure 3A).

We next tested the effects of altering the EMT status of tumor cells on the CDK4 signaling pathway using the previously described isogenic cell line pairs. ZEB1 overexpression in H441 and 393P cells produced higher levels of CDK4 and phospho-RB (Figure 2D). Conversely, miR-200 expression in H1299 and 344SQ cells caused a suppression of the CDK4 axis (Figure 2E). Immunohistochemistry (IHC) on 344SQ syngeneic tumors revealed higher phospho-CDK4 and phospho-RB staining with absent phospho-Erk, and the reverse was observed in 393P syngeneic tumors (Figure 2F). Additionally, we have previously observed that epithelial-like 393P tumors initially respond to MEK inhibitors; however, long-term exposure produces acquired resistance with the acquisition of a mesenchymal phenotype (393P-AZD^R^) (11). 393P-AZD^R^ tumors also showed higher phospho-CDK4 and phospho-RB staining, with suppressed phospho-Erk, an observation similar to the de novo 344SQ mesenchymal-like tumors (Figure 2F). Cell lines derived from 393P-AZD^R^ tumors showed higher phospho-CDK4 and ZEB1 expression, with generally lower levels of phospho-Erk (Supplemental Figure 3B). Resistant cells were no longer sensitive to AZD6244, and instead became sensitive to palbociclib with an IC_50_ similar to 344SQ cells (Figure 2G) and greater suppression of phospho-RB and phospho-CDK4 in 393P-AZD^R^ than in 393P-vehicle cell lines. In contrast there was an accumulation of phospho-CDK4 in 393P-vehicle cells (Supplemental Figure 3C).

We next tested if there were phenotypic differences in the manner in which epithelial-like and mesenchymal-like cell lines undergo cell cycle progression. Upon serum starvation for up to 48 hours, 393P cells almost completely (~90% of the cells) arrested in the G_0_/G_1_ phase of the cell cycle with a complete suppression of the CDK4 pathway (Supplemental Figure 3, D and E). In contrast, 344SQ cells resisted cell cycle arrest in serum-free conditions, with approximately 80% of the cells in G_0_/G_1_ state but 20% of cells continuing to cycle through S or G2/M (Supplemental Figure 3D). This observation corresponded to higher levels of CDK4, Cyclin D1, and phospho-RB in the cells when assayed by subcellular fractionation (Supplemental Figure 3E), suggesting that CDK4 activity in mesenchymal tumor cells could be uncoupled from extrinsic mitogenic signals. After release of cells from the arrested state by addition of serum-containing media, 344SQ cells transitioned into S phase more readily (within 20 hours) than 393P cells, which remained arrested in G1 phase up to 36 hours before returning to the baseline cycling state (Supplemental Figure 3D). Although cell cycle arrest in G1 phase with palbociclib was essentially similar between 393P and 344SQ cells, a significant increase in the percentage of 344SQ cells in apoptotic cells was detected (Supplemental Figure 3, F and G), corresponding to increased cleaved caspase-3 (Figure 1F). We conclude from the above findings that tumor cells with a mesenchymal-like phenotype, either due to intrinsic factors or arising from epithelial cells undergoing EMT as an adaptive resistance mechanism, have rewired survival pathways to activate CDK4 signaling that is independent of mitogenic signals.

### ZEB1 regulates p21 expression and causes differential CDK4 pathway activation.

To identify the mechanistic basis of the differential dependency on the CDK4 pathway between the phenotypic epithelial and mesenchymal cancer cells, we investigated the canonical upstream survival pathways, including AKT, PIK3CA, MAPK11, and FGFR, but did not observe differential regulation between epithelial-like and mesenchymal-like cells. We then focused on the intrinsic regulators of CDK4 activity, p21 (*WAF1/CIP1*) and p27 (*KIP1*), and transiently knocked down each in epithelial-like and mesenchymal-like cells to determine the effects on the CDK4 pathway (Supplemental Figure 4A). p21 knockdown had a more significant impact on the phosphorylation of CDK4 and RB compared with p27 (Supplemental Figure 4B). The much higher phosphorylation of RB in 393P cells with p21 knockdown indicated that p21 maintains a check on the CDK4/RB pathway in the epithelial-like cells and when disrupted activates the CDK4 pathway. Loss of p21 in mesenchymal-like cells only modestly increased phosphorylation of RB compared to the control cells, suggesting that an intrinsic deficiency of p21 protein in the mesenchymal cells could lead to a dysregulated CDK4 pathway. Immunofluorescence assays on 393P, 393P-AZD^R^, and 344SQ cells revealed that a higher percentage of epithelial-like cells had nuclear p21 than mesenchymal-like cells, along with higher colocalization of CDK4 and p21 in epithelial-like tumor cells (Supplemental Figure 4, C and D). Alteration in the tumor suppressor *TP53* is one of the most commonly occurring comutation events in *KRAS*-driven lung cancer, and p21 is a direct target of p53. Therefore, we investigated if there was any effect of p53 on CDK4 pathway in the epithelial-like and mesenchymal-like tumor cells. With transient knockdown of p53, there was no significant difference in downstream CDK4 signaling (Supplemental Figure 4, E and F). We also utilized previously published *Kras^G12D^* mutant (K1) and *Kras^G12D^/p21^–/–^* (KC3 and KC4) murine tumor cells (21). Absence of p21 in tumor cells sensitized KC cell lines to palbociclib with an increase in CDK4 signaling (Supplemental Figure 4, G and H), emphasizing that p21-mediated CDK4 dysregulation was independent of p53 control.

**Figure 3:**
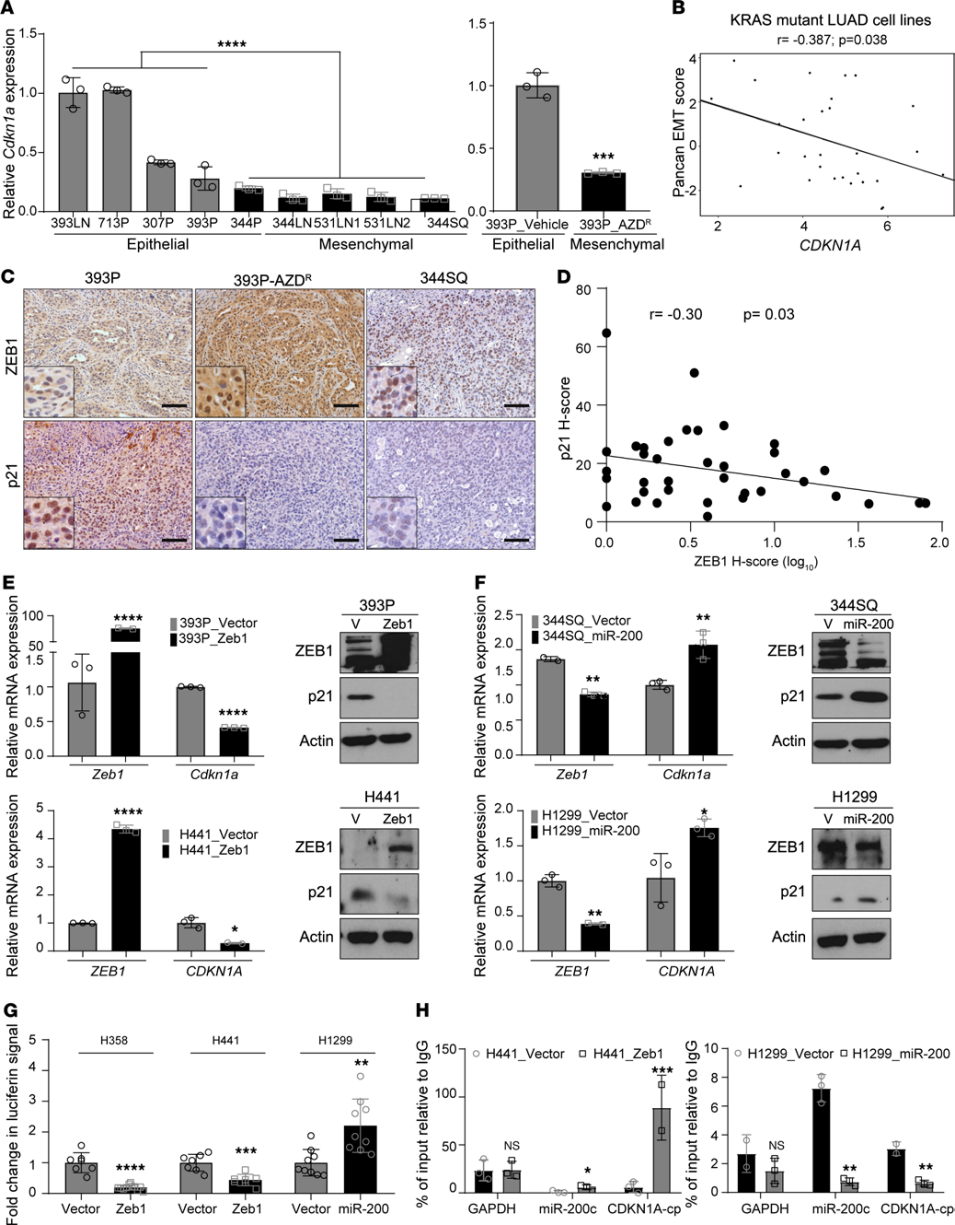
ZEB1 regulates p21 expression and causes differential CDK4 pathway activation. (**A**) Relative expression of *Cdkn1a* mRNA in a panel of murine lung cancer cells and 393P vehicle and AZD^R^ cell lines. (**B**) Cluster plot analysis of correlation between *CDKN1A* mRNA and EMT score in 29 KRAS-mutant human lung adenocarcinoma cell lines. (**C**) IHC on tumors derived from 393P, 344SQ, and 393P-AZD^R^ cells. Scale bar: 50 μm. (**D**) Cluster plot analysis of correlation between ZEB1 and p21 H-scores in NSCLC specimens. (**E** and **F**) Relative expression of *Cdkn1a* mRNA and p21 protein levels upon induction of EMT or MET. ZEB1 induction for 48 hours with 2 μg/mL of doxycycline in murine and human epithelial lung cancer cell lines. miR-200 induction for 48 hours with 2 μg/mL of doxycycline in murine and human mesenchymal lung cancer cell lines. (**G**) Luciferase reporter assay to determine relative luciferase activity of *CDKN1A* promoter reporter construct transfected into epithelial H358 and H441 cells with induced ZEB1 expression or mesenchymal H1299 with induced miR-200 expression. Relative luciferin signal was normalized to promoter-less vector control signal. (**H**) Fold enrichment by qPCR analysis of CDKN1A promoter containing ZEB1 binding site after endogenous ZEB1 ChIP in H441 cells with inducible ZEB1 expression or H1299 cells with inducible miR-200 expression, using ZEB1 antibody or IgG control antibody. Data are presented as mean ± SD, and 1-way ANOVA was used for statistical analysis in all the panels. ****P < 0.0001; ***P < 0.005; **P < 0.001; *P < 0.05.

Next, we inquired whether EMT status of tumor cells could directly regulate the expression of p21. Our previously published microarray data sets that interrogate differential gene expression in epithelial-like and mesenchymal-like tumor cells (10) demonstrated that epithelial-like cells have a higher expression of *Cdkn1a* (gene encoding p21), including comparisons of 344SQ versus 393P cells (fold change 0.57, *P* = 0.003) and 393P-ZEB1 versus 393P-vector (fold change 0.27, *P* < 0.0001). We confirmed and extended this observation with a panel of murine cell lines by quantitative PCR (qPCR) and found that p21 levels inversely correlated with ZEB1 levels across the panel (Figure 3A and Supplemental Figure 5A). Analysis of *CDKN1A* mRNA expression in 29 *KRAS*-mutant human lung adenocarcinoma cell lines revealed an inverse correlation with the previously published EMT gene signature (Figure 3B) (18). IHC analysis of 393P, 344SQ, and 393P-AZD^R^ tumors also showed an inverse correlation between p21 and ZEB1 levels (Figure 3C). Pathological analysis of human NSCLC samples for ZEB1 and p21 by IHC staining revealed an inverse correlation between nuclear ZEB1 and p21 H-scores (Figure 3D). We also grouped the samples based on low ZEB1 (<4 H-score) or high ZEB1 (>4 H-score) staining and found a significant difference in p21 H-score (Supplemental Figure 5B).

We further tested this observation by inducing EMT or mesenchymal-epithelial transition (MET) via overexpression of ZEB1 or miR-200, respectively, in human and murine isogenic cell line pairs. We observed p21 mRNA and protein repression with ZEB1 expression. Conversely, with miR-200 expression, there was an upregulation of p21 (Figure 3, E and F; and Supplemental Figure 5, C and D). Transient and stable knockdown of ZEB1 in human and murine cells, respectively, caused p21 expression (Supplemental Figure 5, E–G). Cells treated with the histone deacetylase inhibitor mocetinostat undergo an MET by upregulation of the miR-200 family and ZEB1 suppression (11, 22). Treatment also produced increased expression of p21 (Supplemental Figure 5, H–J). Induction of miR-200 in tumor cells by different means pushes the cells to a more epithelial state, which is generally considered a less aggressive phenotype for tumor cells and more akin to a “normal” cell state, with expression of p21 and restoration of the cell cycle checkpoint that is lost or blunted in mesenchymal-like tumor cells with high ZEB1 activity.

Luciferase reporter assays were utilized to investigate ZEB1-mediated transcriptional regulation of p21. The promoter region of p21 was subcloned upstream of a luciferase reporter and transfected into human lung cancer cells with either ZEB1 or miR-200 expression. H358 and H441 cells expressing ZEB1 led to a decrease in relative luciferin signal, demonstrating transcriptional repression of the p21 promoter in the presence of high ZEB1. Conversely, an increase in luciferin signal was detected in H1299 cells with miR-200 induction, which suppresses the endogenous cellular ZEB1 expression and relieves transcriptional repression of the p21 promoter (Figure 3G). Binding of ZEB1 to the endogenous p21 promoter was confirmed by ChIP qPCR assays in cells with inducible ZEB1 or miR-200 expression, using previously published primer pairs (23). Using GAPDH as the negative control and miR-200c as a positive control, we showed direct binding of ZEB1 to the p21 promoter (Figure 3H). Altogether the data support the EMT-dependent regulation of p21 in tumor cells by specific and direct ZEB1 binding.

### Suppression of p21 in mesenchymal cells regulates CDK4 pathway.

**Figure 4:**
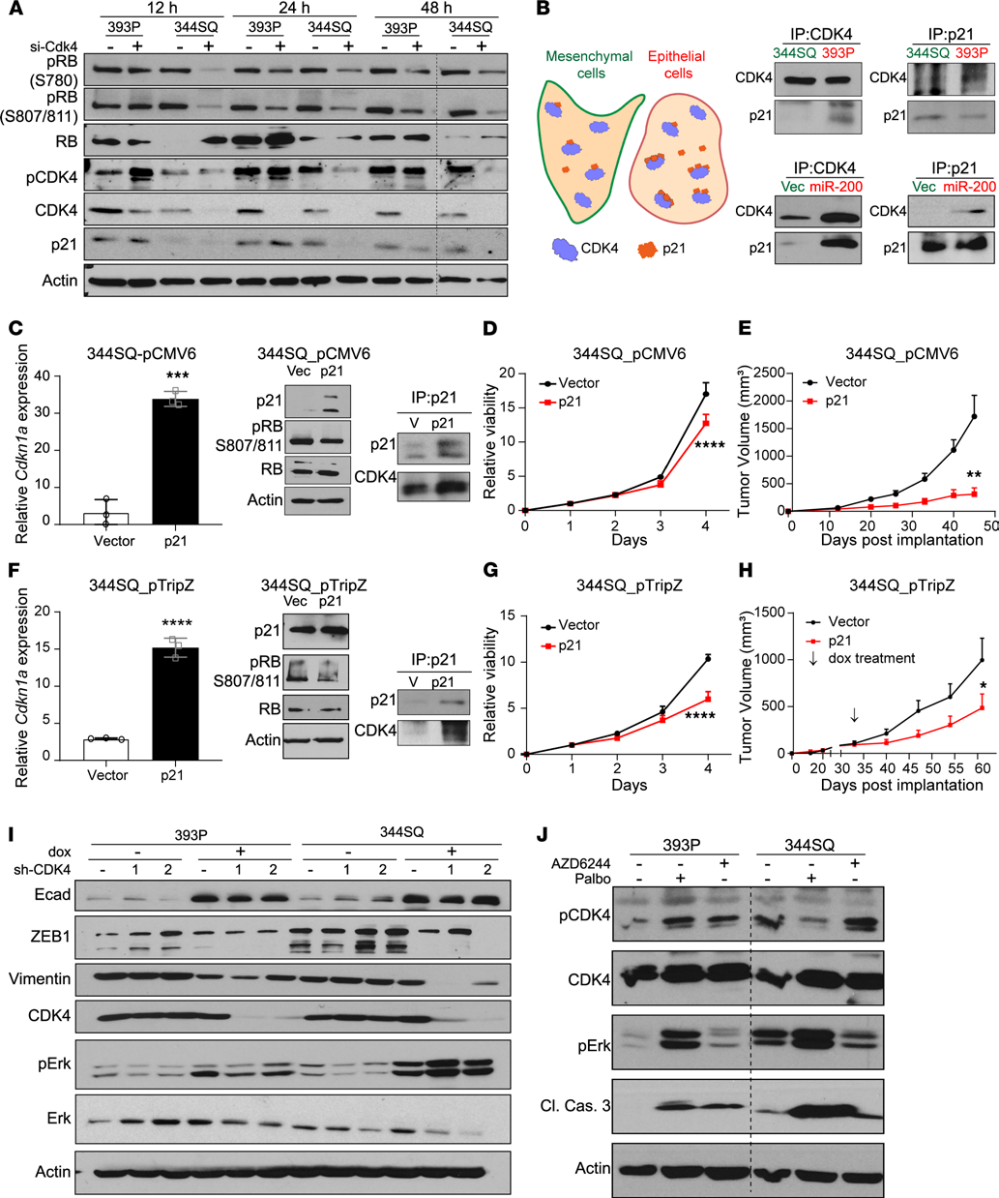
Suppression of p21 in mesenchymal cells regulates CDK4 pathway. (**A**) Transient knockdown of CDK4 using 20 nM siRNAs for indicated times followed by Western blot analysis. (**B**) Graphical representation of the differences in CDK4-p21 complex formation in epithelial and mesenchymal cancer cells. Coimmunoprecipitation (co-IP) of endogenous CDK4 and p21 in epithelial (393P and 344SQ_miR-200) and mesenchymal (344SQ and 344SQ_vec) cell lines. (**C**) Constitutive overexpression of *Cdkn1a* in 344SQ cell lines. Relative *Cdkn1a* mRNA expression, Western blot analysis of CDK4 pathway, and co-IP of CDK4 and p21 in 344SQ cells. (**D**) Growth rates of 344SQ cells ± p21 constitutive overexpression over 4 days measured by water-soluble tetrazolium salt assay. (**E**) Tumor volume measurements at indicated time points of 344SQ tumors ± p21 constitutive expression (*n* = 5 per group). Data are presented as mean ± SEM. (**F**) Doxycycline-induced overexpression of *Cdkn1a* in 344SQ cell lines for 48 hours. Relative *Cdkn1a* mRNA expression, Western blot analysis of CDK4 pathway, and co-IP of CDK4 and p21 in 344SQ cells. (**G**) Growth rates of 344SQ cells ± p21 overexpression (doxycycline induced) over 4 days measured by WST-1 assay. (**H**) Tumor volume measurements at indicated time points of 344SQ tumors ± p21 expression with doxycycline feed (*n* = 9–10 per group). Doxycycline feed was started after tumors reached a size of 100–150 mm^3^ (indicated by arrow). Data are presented as mean ± SEM. (**I**) Western blot analysis of 393P and 344SQ cells with CDK4 knockdown for 7 days. (**J**) Western blot analysis on 393P and 344SQ cells treated with AZD6244 (5 μM) and palbociclib (5 μM) for 48 hours. Data are presented as mean ± SD unless otherwise indicated. Statistical analysis (**C**, **E**, **F**, and **H**): unpaired 2-tailed Student’s *t* test and (**D** and **G**): 2-way ANOVA test. ****P < 0.0001; ***P < 0.005; **P < 0.001; *P < 0.05.

We next explored the effect of p21 on CDK4 activity in epithelial and mesenchymal lung cancer cells. With transient knockdown of CDK4, phosphorylation of RB was continuously suppressed in 344SQ cells for 48 hours (Supplemental Figure 6A and Figure 4A). On the contrary, CDK4 knockdown in 393P cells appeared to have only slightly muted downstream signaling, which coincided with a surprising accumulation of phospho-CDK4, even with very low levels of total CDK4 protein. This also corresponded to a continued presence of p21 protein in 393P cells (Figure 4A). These intriguing findings recapitulated previous data by Bisteau et al. (24), which showed that a sustained presence of p21 protein in cells was able to maintain the phosphorylation status of CDK4 (and hence the stability of the complex) but still inhibit its kinase activity. A similar observation was made in our epithelial-like, but not mesenchymal-like, model where the presence of p21 maintained CDK4 in a phosphorylated state. To further demonstrate this point, we coimmunoprecipitated CDK4 and p21 from mesenchymal-like and epithelial-like cancer cells (Figure 4B). In 344SQ and 344SQ_vector cells, lower amounts of CDK4-p21 complex coimmunoprecipitated compared with 393P and 344SQ_miR-200 cells, where an increased binding of CDK4 and p21 was detected. We also observed the seemingly contradictory presence of phospho-RB in epithelial-like cells alongside p21 expression. An explanation for this observation is the sequestration of p21 into the CDK4 complex, alleviating the repression from the CDK2–Cyclin E complex, which can phosphorylate RB to maintain cell cycle progression. In fact, we observed that epithelial cells are more sensitive to the CDK2 inhibitor (miciclib) than mesenchymal cells (Supplemental Figure 6B), indicating that CDK2 may be the primary regulator of cell cycle in epithelial cells. A similar outcome was observed with pharmacological inhibition of CDK4 (Supplemental Figure 6C), including a partial suppression of phospho-RB and an accumulation of phospho-CDK4 with the presence of p21 in 393P cells, versus a lack of p21 with a near complete suppression of phospho-RB in the 344SQ cells by 48 hours (Supplemental Figure 6C). Lower binding of p21 to CDK4 in mesenchymal-like cancer cells is only sufficient to maintain the activity of the CDK4 complex but is not enough to exert an inhibitory effect on the downstream pathway.

Constitutive (344SQ_pCMV6) or doxycycline-inducible (344SQ_pTripZ) expression of p21 in 344SQ cells was used to determine the direct effect of p21 on CDK4 activity. Upon p21 expression, there was suppression of phospho-RB and phospho-CDK4, which correlated with detection of higher amounts of the CDK4-p21 complex (Figure 4, C and F). These cells also demonstrated slower in vitro growth compared with the vector only cells (Figure 4, D and G) and a decreased sensitivity to palbociclib (Supplemental Figure 6, D and F). When the cells were subcutaneously implanted in syngeneic WT mice, the p21-overexpressing tumors grew significantly slower (Figure 4, E and H; and Supplemental Figure 6, E and G), with about one-third of tumors undergoing complete regression. We also generated 393P cells with stable or doxycycline-inducible knockdown of p21 (Supplemental Figure 6, H and L). A modest increase in phospho-RB was detected with p21 knockdown (Supplemental Figure 6, I and M), along with slightly higher growth rates (Supplemental Figure 6, J and N) and enhanced sensitivity of 393P cells to palbociclib (Supplemental Figure 6, K and O).

We next tested the long-term effect of inhibiting CDK4 on the EMT status of tumor cells. With doxycycline-mediated induction of CDK4 shRNA for 7 days, we observed a shift toward an epithelial phenotype indicated by decreased ZEB1 and vimentin levels and an accumulation of phospho-Erk (Figure 4I). We also generated palbociclib-resistant cells with treatment of 344SQ cells for approximately 4 weeks. An epithelial phenotype was observed with an increase in E-cadherin and decrease in ZEB1 and vimentin levels (Supplemental Figure 7, A–C). Therefore, targeting CDK4 allows the tumor population to shift to a more epithelial state, which would prime the tumor cells for MEK inhibitor treatment. To test this, we transiently knocked down CDK4 and treated cells with AZD6244, which sensitized the previously unresponsive mesenchymal-like 344SQ and 344P cells to MEK inhibition (Supplemental Figure 7D). We then tested the effect of combination palbociclib and AZD6244 treatment using a series of fixed concentrations at 1:1 ratio and calculated the fraction affected (Fa) values after exposure to the drugs. The Chou-Talalay method (25) was used to determine the combination index (CI) and drug reduction index (DRI). The favorable DRI, shown in yellow (Supplemental Figure 7E), was used to confirm the CI data. The drug combinations showed favorable DRI (DRI > 1) and evidence of synergism (CI < 1) at Fa > 0.5 for palbociclib and AZD6244 (Supplemental Figure 7E). Additionally, when tumor cells were treated with single-agent MEK or CDK4 inhibitor, there was a reciprocal activation of the CDK4 or MEK signaling pathways, respectively (Figure 4J), showing a dynamic switching of signaling pathway activation and survival dependencies in the face of pharmacological treatments.

### Cotargeting CDK4 and MAPK pathways targets different tumor cell subsets.

**Figure 5:**
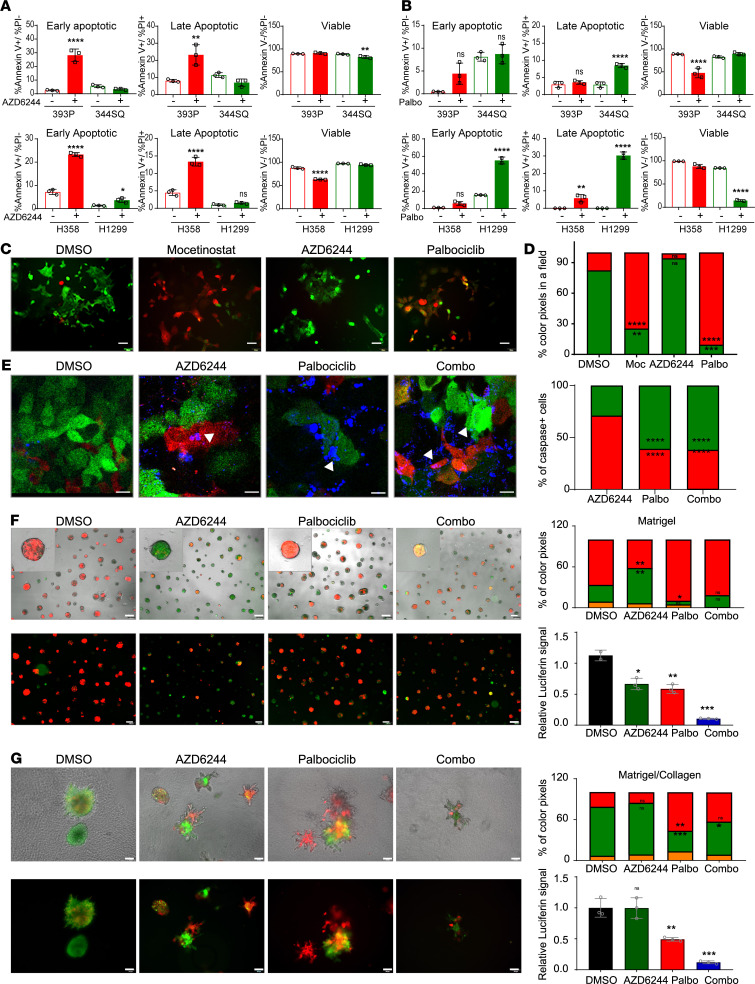
Cotargeting CDK4 and MAPK pathways targets different tumor cell subsets. (**A** and **B**) Apoptosis was determined by annexin V and propidium iodide staining after treatment with AZD6244 (5 μM) or palbociclib (5 μM) for 48 hours. Data are presented as the mean ± SD. (**C**) 344SQ_Z-cad cells were treated with DMSO, mocetinostat (1 μM), AZD6244 (5 μM), or palbociclib (5 μM) for 48 hours followed by fluorescent image acquisition. Scale bar: 50 μm. (**D**) Images from **C** were quantified for the percentage of RFP or GFP colored pixels calculated per field of view (FOV). *n* = 4–6 FOVs. (**E**) 344SQ_Z-cad cells were treated with DMSO, AZD6244 (5 μM), palbociclib (5 μM), or the combination. NucView 405 Caspase 3 substrate was added to each condition as a readout for apoptosis. Representative fluorescent images were acquired 48 hours after addition of drugs. Scale bar: 25 μm. Arrows indicate apoptotic cells. Images were quantified for total caspase-3^+^ cells as a percentage of total cells in 4–6 FOVs. (**F** and **G**) EVTs were plated in a Matrigel (MG) or a collagen/Matrigel (Coll/MG). After 24 hours, EVTs were treated with DMSO, AZD6244 (5 μM), palbociclib (5 μM), or the combination. Treatment in MG was continued for 9 days and Coll/MG for 5 days, with representative images from last day of the culture shown (scale bar: 200 μm for MG and 100 μm for Coll/MG). Red color indicates epithelial-like cells and green color indicates mesenchymal-like cells. Quantification of percentage RFP and GFP colored pixels in 4–6 FOVs. Data are represented as mean. At the end of the treatment, CellTiter-Glo reagent was added and relative luciferin signal was measured. Treatment groups were compared with DMSO using 1-way ANOVA in all the panels. ****P < 0.0001; ***P < 0.005; **P < 0.001; *P < 0.05.

To test if the pharmacological inhibitors have differential apoptotic effects on tumor subpopulations, we treated human (H1299 and H358) and murine (393P and 344SQ) tumor cells with AZD6244 and palbociclib and stained the cells with annexin V and propidium iodide. Mesenchymal-like tumor cells underwent greater apoptosis in response to CDK4 inhibitors, while epithelial-like tumor cells were highly sensitive to MEK inhibition (Figure 5, A and B; and Supplemental Figure 8, A and B).

Given that tumors are heterogeneous and consist of subpopulations with distinct phenotypes along a spectrum of EMT, we utilized a previously described sensor model that can detect the epithelial or mesenchymal state of individual tumor cells in real time (12, 26). Briefly, the 344SQ_Z-cad cell line expresses dual fluorescence sensors: a destabilized GFP with the ZEB1 3′-UTR cloned downstream and E-cadherin promoter driving expression of RFP. This tool exploits the ZEB1/miR-200 double-negative feedback loop. In an epithelial state, with high miR-200 and E-cadherin, the cells express RFP and emit red fluorescence, and the presence of miR-200 suppresses GFP production by binding the ZEB1 3′-UTR to prevent translation. Conversely, in a mesenchymal state with high ZEB1 and low miR-200, cells emit green fluorescence on account of GFP translation, whereas ZEB1 binds to the E-cadherin promoter to suppress transcription of RFP. As seen in Figure 5C, the majority of cells in 2-dimensional (2D) culture were mesenchymal and GFP^+^. With mocetinostat treatment, there was an enrichment of RFP^+^ epithelial cells. There was a reduction of epithelial RFP^+^ cells with AZD6244 treatment and mesenchymal GFP cells with palbociclib treatment (Figure 5, C and D). With dose escalation of single-agent treatment, reciprocal pathway activation occurred, while combination treatment with both drugs suppressed MAPK and CDK4 pathways and enhanced tumor cell killing (Supplemental Figure 8C). Since Western blots are bulk assays, we wanted to assess which specific populations undergo apoptosis within this heterogeneous dynamic system. We utilized a DNA binding dye that is cleaved by caspases present in the cells undergoing apoptosis to produce blue fluorescence. Colocalization of blue/green fluorescence with palbociclib treatment and blue/red fluorescence with AZD6244 treatment demonstrated the specificity of each individual drug to target specific cell types, whereas the combination of both drugs targeted both subpopulations (Figure 5, D and E; and Supplemental Figure 8, D and E).

In vitro 3D assays very closely recapitulate the tumor growth in vivo. An established ex vivo tumor (EVT) model to culture lung tumors that retains tumor cell heterogeneity (27) was utilized to test the therapeutic sensitivity of distinct tumor cell subpopulations (Figure 5, F and G). Similar to the observations in 2D cultures, we found different subpopulations targeted by individual drugs when EVTs were cultured in laminin-rich MG. Since MG is known to promote an epithelial phenotype (10, 27, 28), AZD6244 effectively eliminated this cell subtype and resulted in an enrichment of GFP^+^ cells. Palbociclib conversely caused a depletion of mesenchymal tumor cells within the heterogeneous EVTs and enrichment of the RFP^+^. We also noted a change in phenotype of EVTs treated with palbociclib, producing more structures with a central lumen as compared with other groups (Supplemental Figure 8F). Lumen formation and organization in a 3D matrix are characteristic of epithelial phenotype. Clearly, treatment with palbociclib not only targets mesenchymal phenotype but also promotes an epithelial phenotype, which makes it ideal to be combined with AZD6244. In combination treatment, both populations were targeted, which produced a net decrease in size and viability of EVTs (Figure 5F and Supplemental Figure 8G). EVTs were also cultured in a matrix containing MG/collagen type I, and as previously noted collagen promotes a mesenchymal phenotype in tumor cells (20, 27), allowing us to test the efficacy of palbociclib on this specific subpopulation. AZD6244 remained ineffective on the GFP^+^ mesenchymal tumor cells; however, there was a significant reduction in the viability of EVTs with palbociclib treatment (Figure 5G). Combination treatment proved to be significantly better over the individual treatments in both MG and collagen matrices in terms of suppression of viability of tumor cells. In summary, these results demonstrate the efficacy of CDK4 and MEK inhibitors in combination for effective therapeutic targeting of the tumor cell subpopulations.

### Combination of CDK4 and MEK inhibitors controls syngeneic tumor growth and prevents emergence of EMT-mediated resistance.

**Figure 6:**
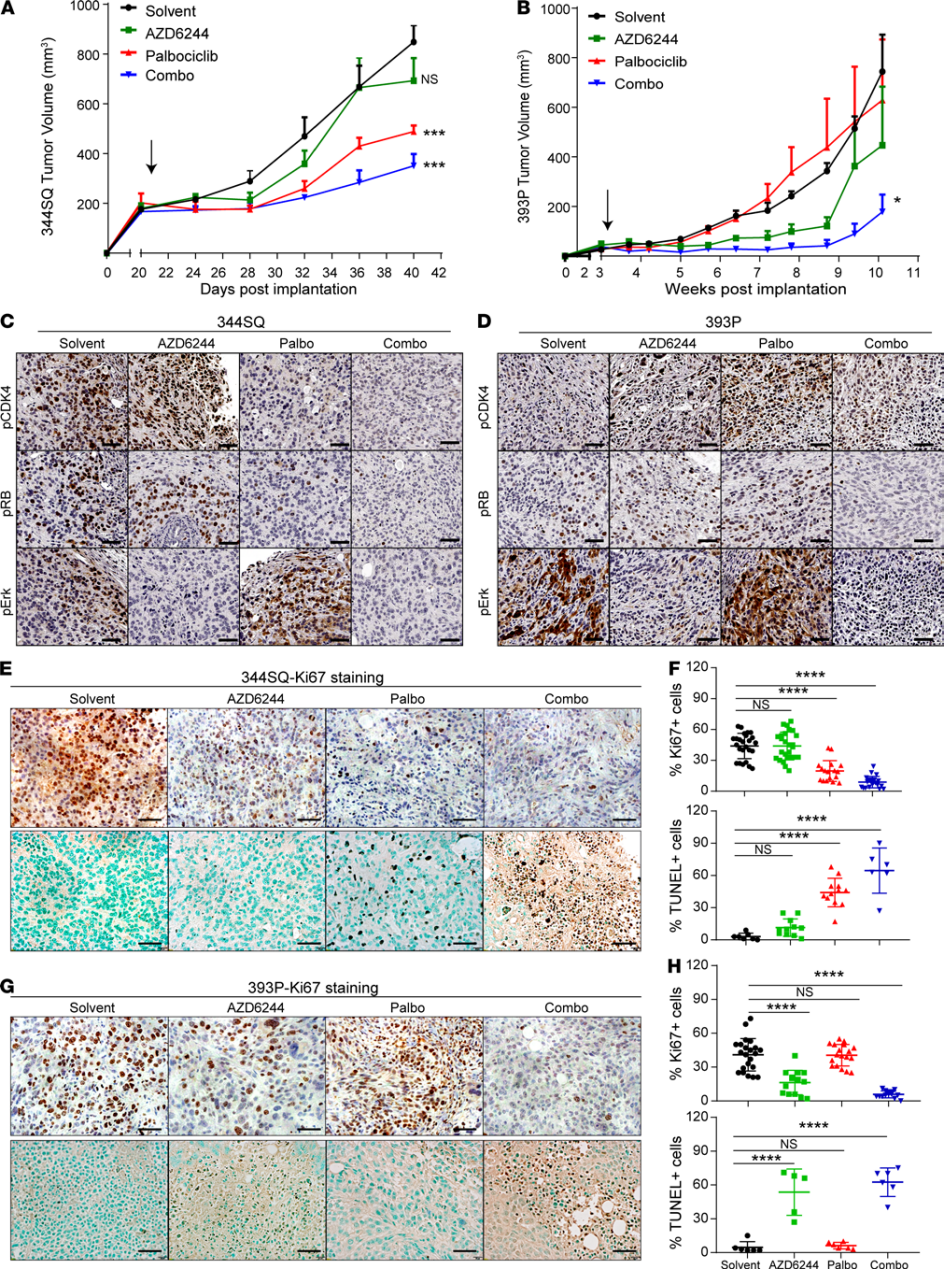
Combination of MEK and CDK4 inhibitors controls syngeneic tumor growth and prevents emergence of EMT-mediated resistance. (**A**) In vivo volume measurements at the indicated time points for 344SQ subcutaneous tumors in syngeneic WT mice (*n* = 5 per group) after daily treatment with solvent, AZD6244 (25 mg/kg), palbociclib (50 mg/kg), or combination. Arrow indicates start of the treatment. (**B**) In vivo volume measurements at indicated time points for 393P subcutaneous tumors in syngeneic WT mice (*n* = 5 per group) after daily treatment with solvent, AZD6244 (25 mg/kg), palbociclib (50 mg/kg), or combination. Arrow indicates start of treatment. (**C** and **D**) IHC analysis on 344SQ and 393P tumors harvested from **A** and **B**, respectively, with indicated markers. Scale bar: 50 μm. (**E**–**H**) Tumors from the experiments described in **A** and **B** were stained with Ki67 and TUNEL assay to measure cell proliferation and cell death respectively. Representative IHC images are shown (**E** and **G**). Scale bar: 50 μm. Images were quantified for Ki67 and TUNEL staining in each treatment group. *n* = 2–3 per group with 3–6 FOVs per mouse. Data are presented as mean ± SD. Statistical significance was determined by 1-way ANOVA in all the panels. ****P < 0.0001; ***P < 0.005; *P < 0.05.

We next evaluated in vivo tumor response to the combination of CDK4 and MEK inhibitors. Mesenchymal-like (344SQ) or epithelial-like (393P) tumor cells were subcutaneously implanted in syngeneic WT mice. Tumor growth in response to either single agent (palbociclib or AZD6244) or both was monitored over a period of 6–14 weeks. Mice bearing 344SQ tumors remained unresponsive to AZD6244 but responded to palbociclib alone or in combination with AZD6244 (Figure 6A). This treatment continued for approximately 6 weeks (short term) and scored as additive using Bliss effect analysis (Supplemental Figure 9D) (29). This promising tumor response in the short term led us to repeat the experiment to determine if there was a durable and sustained response to the combination treatment. Treatment of the cohorts for up to 10 weeks produced the emergence of resistance to palbociclib treatment alone (Supplemental Figure 9, A and B). The tumors acquired resistance to single-agent palbociclib over an extended period, which was prevented with combination treatment, and the group initially treated with only palbociclib was resensitized upon addition of AZD6244 at week 10, measured by tumor growth or fold change of tumor volume (Supplemental Figure 9, A and B). We also observed an increase in E-cadherin and a decrease in nuclear ZEB1 with single-agent palbociclib treatment (Supplemental Figure 9C), demonstrating the selective outgrowth of an epithelial phenotype. The combination treatment for a period of 14 weeks scored as an additive response (Supplemental Figure 9D). The number of lung metastatic nodules in short- and long-term experiments was also significantly lower with palbociclib or combination treatment (Supplemental Figure 9, E and F).

Epithelial-like 393P tumors that initially respond to AZD6244 develop resistance to treatment by undergoing EMT (11). When treated with single agents, 393P tumors were resistant to palbociclib alone and responded to AZD6244 for about 7 weeks (Figure 6B). However, the combination of both the drugs suppressed tumor growth with a durable response for approximately 10 weeks. In the 393P tumor model, combination of CDK4 and MEK inhibitor scored as synergistic using the Bliss effect analysis (Supplemental Figure 9D). Since 393P is a nonmetastatic model, there were no significant differences in lung metastases (Supplemental Figure 9, E and F). The previously described 393P-vehicle and 393P-AZD^R^ cells were also implanted in syngeneic WT mice to assess the sensitivity to CDK4 and MEK inhibitors. 393P-vehicle tumors retained their sensitivity to AZD6244 and resistance to palbociclib (Supplemental Figure 10A), whereas the tumors derived from 393P-AZD^R^ cells were unresponsive to AZD6244, and responsive to palbociclib, with 1 mouse showing complete tumor regression (Supplemental Figure 10B).

Primary tumor tissues were collected at the end of the mouse experiments and stained for the CDK4 and MAPK signaling pathway markers. Untreated 344SQ tumors showed higher phospho-CDK4 and phospho-RB compared with untreated 393P tumors, which had higher phospho-Erk (Figure 6, C and D). In 344SQ tumors, treatment with palbociclib led to suppression of phospho-CDK4 and phospho-RB staining, with an activation of MAPK signaling as marked by phospho-Erk; AZD6244 treatment led to an increase in phospho-CDK4, and the combination drug treatment suppressed both CDK4 and MAPK signaling. Conversely, 393P tumors showed suppression of phospho-Erk when treated with AZD6244, accompanied with an increased expression of phospho-Cdk4. Palbociclib caused an increase in phospho-Erk in 393P tumors as well. Combination drug treatment in both models markedly suppressed both pathways compared with either single agent (Figure 6, C and D).

To determine the effect of single- and combination-agent treatments on cell proliferation and cell death, we performed Ki67 staining and TUNEL assay on the tumor tissues. 344SQ tumors treated with palbociclib for 6 weeks had fewer proliferating and more apoptotic cells (Figure 6, E and F). 393P tumors treated with AZD6244 had fewer proliferating cells and more apoptotic cells (Figure 6, G and H). However, combination inhibitor treatment in both models significantly suppressed cell proliferation and produced apoptosis in more than 60% of the tumor cells. We also compared the cell proliferation and death in the 344SQ tumors treated long-term (10 weeks) with the single agents and combination. As noted, the 344SQ tumors acquired resistance to palbociclib alone after 10 weeks. This was reflected in the Ki67 and TUNEL staining, which were similar to the single-agent AZD6244-treated 344SQ tumors, which were unresponsive (Supplemental Figure 10, C–F). With coadministration of AZD6244 after 10 weeks of single-agent palbociclib, tumors underwent apoptosis with limited cell proliferation, similar to the tumors treated with combination from the start of the experiment (Supplemental Figure 10, C–F). Tumor growth and histological staining collectively demonstrate the efficacy of utilizing a combinatorial approach for treatment of heterogeneous tumors with different tumor subpopulations.

### Concomitant targeting of CDK4 and MAPK pathways augments response in Kras-mutant autochthonous lung tumors.

**Figure 7:**
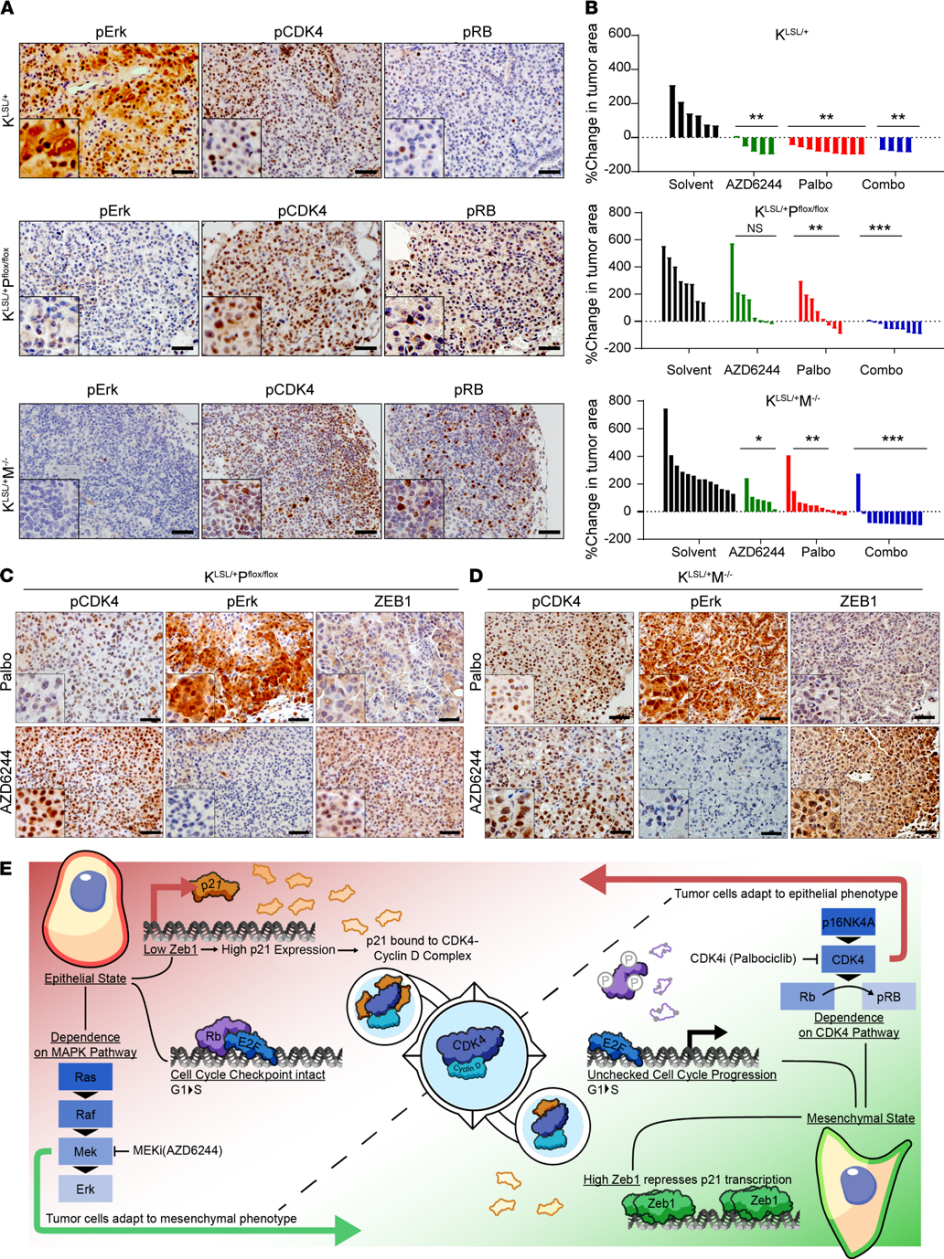
Concomitant targeting of CDK4 and MAPK pathways augments response in Kras-mutant autochthonous lung tumors. (**A**) IHC for indicated markers on *Kras^LSL/+^*, *Kras^LSL/+^ P^flox/flox^*, and *Kras^LSL/+^ M^–/–^* lung sections 18–20 weeks after Ad-Cre infection. Scale bar: 50 μm. (**B**) Percentage change in overall lung tumor area of *Kras^LSL/+^*, *Kras^LSL/+^ P^flox/flox^*, and *Kras^LSL/+^ M^–/–^* mice after 6–8 weeks of daily treatment with AZD6244 (25 mg/kg), palbociclib (50 mg/kg), or both as assessed by micro-CT imaging of mouse lungs. Significance was determined using Brown-Forsythe and Welch ANOVA tests. (**C** and **D**) IHC for indicated markers on lung sections from *Kras^LSL/+^ P^flox/flox^* and *Kras^LSL/+^ M^–/–^* mice treated with AZD6244 and palbociclib for 6–8 weeks. Scale bar: 50 μm. Data are shown as mean. ***P < 0.005; **P < 0.001; *P < 0.05. (**E**) Proposed working model demonstrating differential CDK4 and MAPK signaling pathway activation and sensitivity to CDK4 and MEK inhibitor treatments between epithelial and mesenchymal lung cancer cells due to ZEB1 regulation of p21 expression.

Autochthonous lung tumor models represent powerful and accurate preclinical models for recapitulating human cancer and exploration of treatment efficacy. Three different Kras-mutant genetically engineered mouse models were utilized to interrogate the CDK4 pathway in lung tumors, as well as response to palbociclib alone or in combination with AZD6244. Lung tumors in these conditional models harbor either *Kras* point mutation G12D (*Kras^LSL/+^*; Kras) alone or coupled with homozygous deletion of p53 (*Kras^LSL/+^ P^flox/flox^*; KP) or homozygous deletion of miR-141/200c (*Kras^LSL/+^ M^fl/fl^*; KM) (11, 30) generated through intratracheal administration of adenovirus expressing Cre recombinase (30). Schematic representations of the different alterations are presented in Supplemental Figure 11A. Histological analyses on lung tumors displayed differences in the signaling pathways dependent on the genetic background (Figure 7A). Tumors with mutant Kras alone showed greater MAPK pathway activation compared with KP and KM tumors, which instead showed an activation of CDK4 pathway as demonstrated by phospho-CDK4 and phospho-RB staining (Figure 7A). We also utilized these models to interrogate if the ZEB1/p21 axis was altered within these tumors and could determine their sensitivity to palbociclib. Tumor regions with high nuclear ZEB1 corresponded to lower levels of nuclear p21 in KP and KM tumors, as compared with Kras tumors alone (Supplemental Figure 11B).

Three months after induction, lung tumor formation was confirmed and monitored over 6–8 weeks by micro-CT scans for changes in overall lung tumor burden in response to pharmacological agents. Response to AZD6244 alone across all 3 genotypes was similar to our previous results (11), where Kras tumors showed complete regression upon treatment, and only tumor stability or lack of response was achieved in KP and KM tumors (Figure 7B, Supplemental Figure 11C, and Supplemental Figure 12A). Palbociclib alone had more significant tumor growth control in KP and KM mice than AZD6244 alone with approximately 30% of tumors demonstrating regression (Figure 7B, Supplemental Figure 11C, and Supplemental Figure 12A). Histological staining showed that treatment with each single agent led to an activation of the reciprocal signaling pathway in KP and KM tumors (Figure 7, C and D). Palbociclib led to suppression of ZEB1, indicating a shift to an epithelial phenotype, and AZD6244 led to an accumulation of ZEB1, indicating the presence of mesenchymal-like tumor cells (Figure 7, C and D). Combination palbociclib and AZD6244 produced a more significant reduction of tumors over a period of 8 weeks with complete regression in approximately 80% mice across all 3 genotypes (Figure 7B, Supplemental Figure 11C, and Supplemental Figure 12A). Lack of sufficient tumor burden precluded us from staining the lung sections obtained from combination treatments (Supplemental Figure 11C).

Figure 7E shows the working model for the activation of the different survival pathways in the tumor cell subpopulations in relation to the phenotypic EMT spectrum. Normal cells or epithelial-like cancer cells have an intact cell cycle regulation mediated by the intrinsic regulator p21. Increased binding of p21 to CDK4 prevents the kinase activity, limits RB phosphorylation, and arrests cells in the G_1_ phase. However, this pathway is dysregulated in cancer cells undergoing EMT. ZEB1 is highly upregulated in mesenchymal-like cancer cells, which exerts transcriptional repression on p21. Lack of p21 leads to low or no binding of p21 to CDK4, allowing the kinase activity of CDK4 to occur unchecked. Such high dependency on CDK4 makes mesenchymal cells especially vulnerable to CDK4 inhibitors such as palbociclib. In heterogeneous tumors, with a mix of plastic epithelial and mesenchymal cancer cells, net tumor killing requires drug combinations that preferentially target the vulnerabilities of each subpopulation (e.g., MEK inhibitors and CDK4 inhibitors) and prevent the outgrowth of resistant cells.

## Discussion

Phenotypic switching and transcriptional rewiring in cancer cells in response to the tumor microenvironment or selective pressures of drug treatments allows the escape of cancer cells from cell death. An understanding of the mechanisms by which tumor cells alter their cellular state and the molecular pathways involved can provide the basis for designing effective therapeutic strategies. EMT is a dynamic phenomenon that contributes to tumor heterogeneity. We demonstrate that lung tumors with high ZEB1 that display mesenchymal phenotype have increased dependence on CDK4 pathway for survival, which renders them especially vulnerable to CDK4 inhibitors. Combined with our previously published results that showed higher sensitivity of epithelial cancer cells to MEK inhibitors (11), we investigated the combination of CDK4 and MEK inhibitors in multiple preclinical models that recapitulate EMT-mediated tumor heterogeneity and demonstrated this as an effective strategy to combat heterogeneity and resistance.

CDK4 plays a key role in determining the progression of cells from G_1_ to S phase of the cell cycle. Disruption of the checkpoint leads to unregulated growth in cancer cells. Ordinarily, the cell cycle is regulated by extracellular mitogenic signals that are integrated by the MAPK pathway (14, 31). However, aberrant CDK4 activation in Kras-mutant mesenchymal-like cancer cells can occur in a cell-autonomous manner, without being coupled with extrinsic signals or the MAPK pathway. Thus, the independent activation of CDK4 serves as a survival mechanism activated in mesenchymal cancer cells allowing escape from MEK inhibitors. Epithelial tumor cells are less dependent on CDK4 for survival, and instead CDK2-dependent RB phosphorylation seems to be the major cell cycle pathway in epithelial cancer cells. A previous study identified that MAPK-mediated activation of CDK2 keeps a check on RB activity and prevents progression of Kras-mutant lung cancers (32). This is in line with our observations in the epithelial cancer cells that have activated MAPK pathway and a proper cell cycle regulation. Although the results from Walter et al. (32) were not studied in the context of EMT, our results show that the epithelial tumor cells are equally receptive to CDK2 and MEK inhibitors, whereas mesenchymal cancer cells are resistant. These findings reiterate the fact that CDK4 and MAPK pathways are closely linked in lung cancer and present an opportunity for therapeutic cotargeting.

Separate studies have presented contradictory findings for the correlation of EMT with CDK4 pathway signaling. CDK4 inhibition in triple-negative breast cancer reversed the EMT status of cancer cells (33, 34), as seen in the mesenchymal-like KP tumors treated with palbociclib in the present study. Within Kras-mutant pancreatic cancer, one study showed that tumor cells underwent EMT with palbociclib monotherapy (35) and MET in another (36). Another study in colorectal cancer noted no difference in EMT status of tumor cells in response to palbociclib (37). These findings highlight the fact that there are cell type– or context-specific phenomena that warrant further investigation in different cancer types. In our studies, we found that modulation of the EMT status of cancer cells by perturbing the ZEB1/miR-200 axis led to CDK4 pathway modulation and determined the sensitivity to CDK4 inhibitors both in vitro and in vivo.

Mechanistically, high ZEB1 levels in mesenchymal cancer cells were responsible for transcriptional repression of p21 by direct binding to the promoter region. Conventionally, p21 is described as a suppressor of CDK4 kinase activity, and downregulation in patients predicts poor survival (38, 39). Studies in recent years have further explored the role of p21 and revealed a dual function of p21, acting in some cases as an activator for CDK4 activity (40). Lower levels of p21 binding are generally required for the assembly and stability of the CDK4–cyclin D complex, which partially accounts for maintaining CDK4 phosphorylation and primes CDK4 for catalysis by releasing the activation segment without affecting kinase function (24). A sustained presence of p21 at higher stoichiometric concentrations can render CDK4 ineffective (24). We found that mesenchymal cancer cells had lower levels of p21 in the CDK4-p21 complex, which explains their increased CDK4 activity. Continued presence of an activated CDK4 rendered the mesenchymal cells highly dependent on CDK4 for survival. With p21 overexpression in mesenchymal cancer cells, we detected increased CDK4-p21 complex and reduced in vitro and in vivo growth of tumors. Interestingly, 344SQ cells demonstrated reduced sensitivity to palbociclib with p21 overexpression. A previous study had shown that p21 can interfere with the binding of small inhibitors to CDK4 complex, as we observed in WT epithelial tumor cells and in p21-overexpressing mesenchymal cells (41). Thus, p21 serves as a regulator of CDK4 activity and sensitivity to inhibitors in mesenchymal lung cancer cells.

With an understanding of how lung cancer cells adapt to therapeutic intervention, we interrogated the combination of CDK4 and MEK inhibitors. The success of CDK4 inhibitors in combination with endocrine therapy in breast cancer patients has encouraged investigations into the role of CDK4 inhibitors in other cancer types, including lung cancer (42–45). Kras-driven murine lung cancers were particularly susceptible to CDK4 ablation, and a sustained tumor response was achieved with concomitant CDK4 inactivation and RAF1 ablation in *Kras/p53*-driven murine lung cancers (16, 17). A phase II trial in NSCLC patients with inactivated *CDKN2A* treated with palbociclib monotherapy showed modest response with stable disease in 50% of the patients (46). Partial response to CDK4 inhibitor in a subset of lung cancer patients warranted an exploration of combination with other targeted therapies. Zhou et al. demonstrated a synergistic growth inhibition in *KRAS* and *CDKN2A* mutant NSCLC xenografts with AZD6244 and palbociclib (47). Ongoing phase I/II clinical trials (NCT03170206 and NCT02022982) in advanced *KRAS*-driven NSCLC patients are investigating the combinatorial effect of MEK and CDK4 inhibitors. Additionally, the combination of CDK4 and MAPK pathway inhibitors has shown tumor regression in xenograft models of cancers with *KRAS*, *NRAS*, or *BRAF* mutations, particularly BRAF- and NRAS-mutant melanoma, with promising results from phase I clinical studies (29, 48–52). Clinical trials are currently investigating BRAF and MEK inhibitors in combination with ribociclib in BRAF-mutant melanoma and other solid tumors with BRAFV600 mutations (53). Not only are the 2 therapies synergistic, but studies have also shown that CDK4 inhibitors may overcome MEK inhibitor resistance (54) and vice versa (55). These findings are corroborated by our results in the present study demonstrating the efficacy of CDK4 and MEK inhibitors.

Results in our immunocompetent syngeneic models will allow us to further extend our investigation into effects on the immune microenvironment. Evidence from past studies indicated that CDK4 depletion reduced infiltration of CD4^+^FoxP3^+^ Tregs (56), and CDK4 inhibitors increased tumor immunogenicity and cytotoxic T cell–mediated clearance of tumor cells (57). CDK4 inhibitors also enhanced effector T cell infiltration and activation (58). Additionally, programmed cell death ligand 1 (PD-L1) degradation was shown to be regulated by CDK4 through cullin3–SPOP E3 ligase via proteasome-mediated degradation, which primed the tumors for effective response to combination treatment with CDK4 inhibitor and PD-(L)1 immune checkpoint blockade (59). Other investigations revealed that PD-L1 expression was modulated by the RB/NF-κB axis, which could be exploited to overcome cancer immune evasion triggered by conventional or targeted therapies (60). Combination of CDK4 and MEK inhibitor induced a senescence-associated secretory phenotype that provoked a natural killer cell surveillance program and resulted in tumor cell death (61).

The application of combinatorial treatments with MEK and CDK4 inhibitors in multiple preclinical in vitro (dual fluorescence sensor system, 3D assays) and in vivo models (syngeneic and autochthonous mouse models) effectively prevented outgrowth of resistant tumor subpopulations and was substantially better than either monotherapy. Such findings demonstrate that CDK4 and MAPK pathways are intertwined in lung cancer progression and durable response can be attained if these pathways are targeted judiciously. Fighting cancer at 2 fronts, by interfering with 2 distinct regulatory networks and targeting tumor subpopulations, should benefit patients and help prevent resistance.

## Methods

Further information can be found in Supplemental Methods.

### shRNA screens.

Murine lung cancer cell lines (393P and 344P) were infected at a multiplicity of infection of 0.3 with a pooled shRNA lentiviral library targeting genes associated with known kinase activity (10 shRNA/gene, for target list and shRNA sequences, see Supplemental Table 2 and Supplemental Figure 4). Parallel in vivo and in vitro screens were performed, and the shRNA-coupled barcodes were detected by high-throughput sequencing technology (for detailed procedures and primer sequences, see ref. 62). In vivo and in vitro screens were carried out in triplicate and duplicate, respectively. Raw counts for the screen endpoints and a reference population, isolated after transduction, were normalized using the variance stabilizing transformation with the DESeq2 in R. The normalized counts were divided by the reference cells that were isolated immediately following transduction to estimate a fold change in barcode abundance. Four independent shRNAs targeting essential genes (*RPL30*, *PSMA1*) or luciferase (LUC) were subcloned with 5 unique barcodes each and incorporated in the library as positive and negative controls (20 reagents/control, see Supplemental Table 1 and Supplemental Figure 3). One *LUC* hairpin showed apparent off-target effect, which has been observed over a wide spectrum of in vitro and in vivo screens. One hairpin for *PSMA1* did not show robust dropout, and this pattern was consistent across the 5 barcodes, indicating that this result was not reflective of poor screen performance. The separation of positive and negative controls was evaluated by the robust strictly standardized mean (Supplemental Table 1 and Supplemental Figure 3), excluding the hairpins mentioned above. Fold change distribution was converted to percentiles, and biological replicates were collapsed for RSA analysis. The RSA log *P* values and ranks are provided in Supplemental Tables 2 and 4.

### Cell lines.

Human and murine lung cancer cell lines were cultured in RPMI1640 (Gibco, Thermo Fisher Scientific) supplemented with 10% fetal bovine serum (FBS, Gibco, Thermo Fisher Scientific). 293T (ATCC) cells were cultured in DMEM (Gibco, Thermo Fisher Scientific) supplemented with 10% FBS. All human cell lines were obtained through ATCC. Murine lung cancer cells were created from *Kras^LA1/+^/p53^R172H^* genetically engineered mice as previously described (10). Manipulated human and murine cell lines with ZEB1 and miR-200 expression were derived as previously described (11). All cells were cultured at 37°C in a humidified incubator at 5% CO_2_. Cell lines with inducible ZEB1, miR-200, sh-CDK4, and sh-p21 expression were treated with a final concentration of 2 μg/mL doxycycline (dox) from MilliporeSigma. *Kras^G12D^* mutant (K1) and *Kras^G12D^/p21^–/–^* (KC3 and KC4) cell lines were provided by Jonathan M. Kurie at MD Anderson Cancer Center, Houston, Texas, USA.

### In vitro drug response and cell growth assays.

Cells were seeded in 96-well plates at 1000 cells per well, and each row was treated with the indicated concentrations of drugs (the first row was the solvent control without any drug). After 48 hours of drug treatments, MTT reagent (MilliporeSigma) was added to each well at a final concentration of 0.5 mg/mL and incubated at 37°C for 1 hour. Color intensity was measured at 570 nm, with 630 nm reading subtracted for background. Percent surviving fraction of cells was normalized against cells treated with solvent control only.

### Synergy determination.

The Chou-Talalay method was used to determine possible synergistic effects between inhibitors (25). CompuSyn software (ComboSyn Inc.) was used to determine synergy between drug combinations. A fixed ratio of 1:1 μM was utilized over the concentration series of 0.03, 0.06, 0.125, 0.25, 0.5, 1, 2 μM. Drug-drug interactions were analyzed based on combination index whereby interactions can be additive (CI = 1), antagonistic (CI > 1), or synergistic (CI < 1). Drug concentrations in the combination were compared to the amount of drug alone required to reach same effects. This was expressed as the dose reduction index, DRI.

### Statistics.

Statistical analysis was carried out as described in each corresponding figure legend. A *P* value of less than 0.05 was considered statistically significant. Data are presented as mean ± SD unless otherwise noted. All analyses were performed in GraphPad Prism software (version 8).

### Study approval.

All animal experiments were reviewed and approved by the Institutional Animal Care and Use Committee at the University of Texas MD Anderson Cancer Center. All mice used in the studies were immunocompetent and assessed for health daily by the Department of Veterinary Medicine and Surgery. All mice were genotyped to determine the mutational status by tail snips 2 weeks after birth. In vivo cells were implanted subcutaneously into the right flanks of 129/Sv mice and allowed to form tumors for 2 to 3 weeks, at which point tumor volumes were approximately 150 to 200 mm^3^ measured using digital calipers. For conditional mouse models of lung adenocarcinoma (*Kras^LSL/+^*, *Kras^LSL/+^ p53^flox/flox^*, and *Kras^LSL/+^ miR-200^flox/flox^*) (previously described in ref. 11), adenovirus expressing Cre recombinase was administered into mouse lungs at 3 months of age by intratracheal intubation at a viral titer of 2.5 × 10^7^ viruses per mouse. At 3 months postinduction, mouse lungs were visualized by micro-CT scans to confirm tumor formation. For drug treatments, mice were randomized to either treatment or vehicle control groups. AZD6244 (Selleckchem) and palbociclib (MedChemExpress) were administered daily by oral gavage at a dosage of 25 mg/kg mouse weight and 50 mg/kg mouse weight, respectively. Tumor sizes were measured weekly. AZD6244 was dissolved at 5 mg/mL in solvent (4% DMSO, 30% PEG 300, 5% Tween 80), and palbociclib was dissolved at 10 mg/mL in solvent (lactic acid buffer, 50 mM, pH 4.0). Control mice received solvent at a volume equal to the drug dosage at the indicated drug concentrations. After euthanasia by CO_2_ exposure at 3 L/min, syngeneic primary tumors and/or mouse lungs were formalin-fixed, paraffin-embedded, and sectioned for histological analysis. In vivo combination synergy analysis was done using the method of Bliss Independence as previously described (29).

## Author contributions

AP and DLG conceived the project and designed experiments. AP wrote the manuscript and performed and/or assisted in all experiments and data analyses. JMK generated p21-overexpression and -knockdown cell lines. BLR assisted with flow cytometry analysis. AP and JJF generated the adeno-Cre–induced mouse lung tumors. BLR and JJF assisted with the animal studies. JKO assisted with IHC staining of murine tissues. LD and JW performed bioinformatics analyses on the RPPA data set and human lung cancer cell line panels. RM, MDP, and AC executed functional genomics screens. CAB processed and analyzed deep sequencing barcode data. WL and LSS performed IHC on human lung cancer specimens and HB performed digital image analysis. MGR provided overall supervision for IHC staining. DLG supervised and oversaw all aspects of the project and the writing of the manuscript.

## Supplementary Material

Supplemental data

## Figures and Tables

**Table 1:**
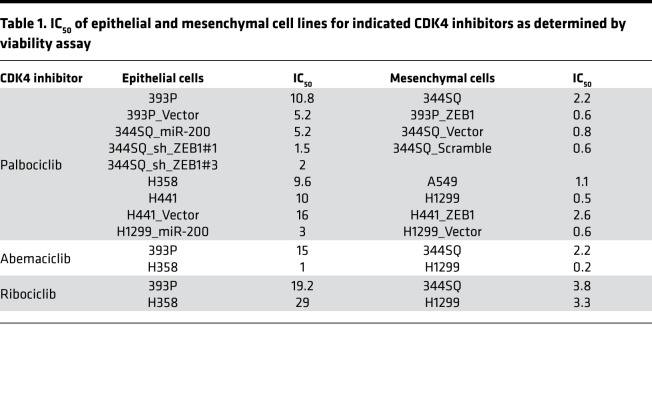
IC_50_ of epithelial and mesenchymal cell lines for indicated CDK4 inhibitors as determined by viability assay

## Version 1 07/26/2021

In-Press Preview

## Version 2 09/08/2021

Electronic publication
