# Supplementary material for: Targeting CDK4 overcomes EMT-mediated tumor heterogeneity and therapeutic resistance in KRAS-mutant lung cancer
Source: JCI Insight. 2021 Sep 8;6(17):e148392. doi: 10.1172/jci.insight.148392 (PMC8492319; doi:10.1172/jci.insight.148392)
Supplement: Supplemental data [file jciinsight-6-148392-s277.pdf]

Targeting CDK4 overcomes EMT-mediated tumor heterogeneity and therapeutic resistance in KRAS mutant lung cancer

Aparna Padhye[1,2], Jessica M. Konen[1], B. Leticia Rodriguez[1], Jared J. Fradette[1], Joshua K. Ochieng[1], Lixia Diao[3], Jing Wang[3], Wei Lu[4], Luisa S. Solis[4], Harsh Batra[4], Maria G. Raso[4], Michael D. Peoples[5], Rosalba Minelli[5], Alessandro Carugo[5], Christopher A. Bristow[5], Don L. Gibbons[1,6*]

1. Department of Thoracic/Head and Neck Medical Oncology, University of Texas MD Anderson Cancer Center, Houston, TX 77030, USA.
2. University of Texas Graduate School of Biomedical Sciences, Houston, TX 77030, USA.
3. Department of Bioinformatics and Computational Biology, University of Texas MD Anderson Cancer Center, Houston, TX 77030, USA.
4. Department of Translational Molecular Pathology, University of Texas MD Anderson Cancer Center, Houston, TX 77030, USA
5. TRACTION Platform, Division of Therapeutics Development, University of Texas MD Anderson Cancer Center, Houston, TX 77030, USA.
6. Department of Molecular and Cellular Oncology, University of Texas MD Anderson Cancer Center, Houston, TX 77030, USA.

*Corresponding author. Email: dlgibbon@mdanderson.org

Supplemental Methods

Plasmids, Transfections, and Lentiviral Generation and Transduction

Transfections of si-RNAs werr performed using the Lipofectamine 2000 Transfection Reagent (Thermo Fisher Scientific). Constitutive Cdkn1a overexpression cell lines were generated by using Cdkn1a mouse Tagged ORF Clone (Origene (NM_007669)). Cdkn1a ORF was also subcloned into dox-inducible pTRIPZ-GFP vector to generate doxycycline inducible cell lines using EcoRI and AgeI restriction cut sites. Constitutive Cdkn1a shRNAs were purchased from Milipore sigma. The sequences used in the experiments are listed in table S11. Dox-inducible shRNAs were expressed in Tet-pLKO-puro vector with a scramble sequence as the non-targeting control. Tet-pLKO-puro was a gift from Dmitri Wiederschain (Addgene plasmid # 21915; http://n2t.net/addgene:21915; RRID: Addgene_21915) (71). CDK4 and Cdkn1a shRNA oligo sequences that were used in the studies are listed in table S11. For lentiviral transductions, viruses were first generated by co-transfecting packaging vector psPAX2, envelope vector pMD2.G, and the pLenti-puro expression vector into 293T cells using Lipofectamine LTX. Transfection medium was removed and 293T cells were cultured in RPMI 1640 supplemented with FBS for 48 hours. Viruses were then syringe-filtered through a 0.45 μm nylon filter and Polybrene (Santa Cruz) was added to a final concentration of 8 μg/mL. Medium containing lentiviruses was then added to cells, left to allow infection of the cells for 48 hours, and replaced with fresh medium for further experiments.

Reverse Phase Protein Array (RPPA) Preparation and Analysis

Cultured cells described above were washed with PBS, and proteins were extracted by the addition of lysis buffer (1% Triton X-100, 50 mM HEPES [pH 7.4], 150 mM NaCl, 1.5 mM MgCl2, 1 mM EGTA, 100 mM NaF, 10 mM NaPPi, 10% glycerol, 1 mM phenylmethylsulfonyl fluoride, 1 mM Na3VO4, and protease and phosphoprotease inhibitors from Roche), incubated on ice for 20 minutes, centrifuged at 14,000 rpm for 10 minutes, and collected for supernatant. Protein concentration was measured using the Pierce BCA Protein Assay Kit (Thermo Fisher Scientific), and protein samples were prepared to a final concentration of 1 μg/μl after mixing with 4x SDS sample buffer (40% glycerol, 8% SDS, 0.25M Tris-HCl pH 6.8, 10% 2-mercaptoethanol) to produce a 1x SDS sample buffer solution. Protein samples were then boiled at 100°C for 5 minutes and stored at -80°C for RPPA processing described here

(https://www.mdanderson.org/research/research-resources/core-facilities/functional-proteomicsrppa-core/rppa-process.html).

RNA Isolation and qPCR Analysis

Cultured cells were washed with PBS, and total RNA was isolated using TRIzol Reagent (Thermo Fisher Scientific). For qPCR analysis of mRNA expression, cDNA was generated from purified mRNA using qSCRIPT reverse transcriptase mix (Quanta Biosciences). QPCR assays were performed using SYBR Green PCR Master Mix (Thermo Fisher Scientific) along with primers in table S9 and normalized to the *L32* gene. All qPCR reactions were performed using the 7500 Fast Real-Time PCR System (Applied Biosystems).

Protein Isolation and Western Blotting Analysis

Cultured cells were washed with PBS, and proteins were extracted from cell lysates using 1xRIPA buffer (Cell Signaling) with protease inhibitors and phosphatase. Samples were loaded onto a 10% SDS polyacrylamide gel, separated by SDS-PAGE, and transferred to a nitrocellulose membrane. Membranes containing the transferred proteins were blocked with 5% w/v fat free dry milk (Bio-Rad) dissolved in TBST. Membranes were probed with the primary antibodies listed in table S5 diluted in 1% w/v bovine serum albumin (BSA) and dissolved in TBST overnight at $4^{O}C$. Next, horseradish peroxidase (HRP)-conjugated secondary antibodies diluted in blocking solution were added to the membranes and incubated at room temperature for 1 hour. HRP-induced chemiluminescence signal was produced using Pierce ECL Western Blotting Substrate (Thermo Fisher Scientific). ECL signal from antibody-probed protein was detected using autoradiography film (BioExpress) and developer.

Subcellular fractionation

Fractionation assay was performed using Cell Fractionation Kit #9038 according to manufacturer instructions.

Cell cycle analysis

Cells were harvested by trypsinization followed by PBS washes. Cold 70% ethanol added to cells dropwise while vortexing was used for fixation for 30 min at $4^{O}C$. Cells were washed with PBS, centrifuged to collect cell pellet. Cells were treated with RNAase and then stained with

propidium iodide for 30 min at room temperature. Cell cycle was analyzed by flow cytometry using FlowJo software (version 10).

Immunohistochemistry

Paraffin-embedded tissue sections were rehydrated, and heat-mediated antigen retrieval was performed using citrate buffer, pH 6.0 (Dako Agilent Technologies). Endogenous peroxidases were blocked with 3% H_2O_2 in TBS, and slides were further blocked with 5% goat serum in TBST. Tissues were probed with primary antibodies listed in table S7, diluted in goat serum overnight at 4°C. Slides were then washed three times with TBST and incubated with streptavidin-conjugated secondary antibodies targeting rabbit IgG diluted in goat serum for 1 hour at room temperature. Slides were washed again and incubated with biotinylated HRP in goat serum for 30 minutes at room temperature. After washing, signal was attained by developing with DAB reagent (Dako) for 5 minutes at room temperature. Slides were washed with ddH_2O to stop the reaction and then stained with Harris Hematoxylin (Thermo Fisher Scientific) for 1 minute and rinsed with warm tap water for 5 minutes. Slides were dipped eight times in 0.25% HCl in 70% ethanol and rinsed with tap water again for 5 minutes. Slides were dehydrated and mounted for further analysis by bright field microscopy.

Immunofluorescence

Cells were plated on poly-L-lysine coated cover slips. Cells were fixed with 100% methanol, permeabilized with 0.3% Triton-X, and blocked with 5% normal goat serum. Primary antibodies listed in table S8 were incubated overnight. Alexa-Fluor conjugated secondary antibodies were used for protein visualization (anti-mouse 546, anti-rabbit 488) and coverslips were mounted with ProLong Gold with DAPI for nuclear stain. Images were taken on the Olympus IX73 using a 40x objective.

Luciferase Reporter Assay

pGL2-p21 promoter-Luc was purchased from Addgene. Human cells (H441, H358 and H1299) expressing ZEB1 and miR-200 were co-transfected with 500 ng of the reporter construct and 50 ng of the pRL-TK renilla luciferase vector (Promega) using Lipofectamine 2000 (Thermo Fisher Scientific). Assays were carried out using Dual-Luciferase Reporter Assay System (Promega), where renilla signal was used as an internal control. Relative luciferin signal was normalized to signal from the empty pGL2 promoter vector control.

3D-assays

EVTs were cultured for one day using microwells as described in (26) followed by plating on a matrix comprised of Matrigel (BD-Biosciences) or a Matrigel/Collagen (1.5 mg/ml) mixture (BD-Biosciences). Media was replenished every 48 hours. Cell viability was measured using Cell Titer-Glo (Promega) following manufacturer recommendations. Luminescence intensity was measured with a Synergy HT microplate reader (BioTek) and normalized to intensities of controls. Spheres were imaged using an inverted microscope. Adobe Photoshop software (RRID:SCR_014199) was used for RFP and GFP pixel analysis by histogram analysis of green and red pixels as a percentage of total colored pixels per field of view (FOV). Minimum number of independent FOVs counted was 4. Spheres were also imaged using an Olympus IX73 microscope.

Apoptosis detection

Annexin and PI staining: Cells were harvested by trypsinization followed by PBS wash and twice with cold BioLegend's Cell Staining Buffer. 5 µL of FITC Annexin V and 10 µL of Propidium Iodide Solution were added to 100 µL of cell suspension followed by gentle vortexing and incubation for 15 min at room temperature in the dark. 400 µL of Annexin V Binding Buffer was added to sample. Analysis was done by flow cytometry using FlowJo software (version 10).

NucView 405 Caspase-3 Substrate assay: For detecting apoptosis in live Zcad fluorescent cells, a blue fluorescent caspase-3/7 substrate was used at 2 µM concentration. Images were acquired using 405 nm laser excitation using an Eclipse Ti inverted microscope with A1+ confocal scanner (Nikon). Flow cytometry was also performed on cells incubated with NucView reagent. Briefly, cells were detached from culture substrate using trypsin. Cells were resuspended in media and NucView substrate at final concentration of 2 µM was added to the cells. Cells were incubated at room temperature for 30 minutes, protected from light followed by analysis by flow cytometry.

Immunoprecipitation assay

Cells were washed twice with PBS on ice, scraped and pelleted. Supernatant was removed and the pellet was lysed in 500 µl lysis buffer [150 mM NaCl, 50 mM Tris-HCl (pH 7.5), 0.5% NP-40, 50 mM NaF, 1 mM Na orthovanate, 1 mM *β*-glycerophosphate, 10% glycerol, PMSF and protease inhibitor] and incubated on ice for 30 min. Samples were sonicated for 1 min and centrifuged at 13,000 rpm for 10 min. The supernatant was collected and precleared for 1 hourat

4°C with non-specific IgG and protein A/G agarose beads. Dynabeads were incubated for 1h with 2µg of antibody at 4°C on a rotating platform. 500 µg of pre-cleared lysate was subjected to immunoprecipitation overnight at 4°C. Antibody-antigen complexes were washed with lysis buffer and then eluted with 2x SDS sample Buffer at 100°C and analyzed by western blot (Supplemental Table 6).

Chromatin Immunoprecipitation (ChIP)

Cells were cross-linked with 1% formaldehyde at room temperature for 10 min. Glycine was added to a final concentration of 0.125 M for 5 min at room temperature. Cells were then washed with PBS with protease inhibitor, scraped and centrifuged (12,000 rpm, 4°C for 2 min). The supernatant was removed and the pellet was resuspended in 1 ml of lysis buffer [50mM Tris-HCl (pH 8.1), 10mM EDTA, 1% SDS, protease inhibitor] incubated on ice for 10 min. Samples were sonicated on ice for 30 cycles at 50% amplitude with 5 seconds pulse intervals and 10 seconds rest intervals. Supernatants were recovered by centrifugation at 12,000 rpm for 5 min. Lysates are diluted 1:10 in ChIP dilution buffer [16.7 mM Tris-HCl (pH 8.1), 16.7 mM NaCl, 1.2 mM EDTA, 0.01% SDS, 1.1% Triton X-100, protease inhibitor]. Sheared DNA was precleared with 2 µg sheared salmon sperm DNA and 30 µL protein A/G beads (sc-2003) at 4°C for 1 hour with rotation. Beads were pelleted, the supernatant was collected and an aliquot (1/20th) of the chromatin preparation was set aside and designated as the Input Fraction. The rest of the sample was divided into parts for incubation with 2 µg rabbit IgG (sc-2027) and ZEB1 (santacruz) antibodies overnight at 4°C with rotation. The immune complexes were captured the next day by incubation with 30 µL of pre-cleared beads and 2 µg sheared salmon sperm DNA for 2 h at 4°C. Beads were pelleted by centrifugation for 1 min at 4°C at 100 g and washed sequentially for 10 min at 4°C with rotation with 1 ml of the following buffers: low salt wash buffer [20 mM Tris-HCl (pH 8.1), 150 mM NaCl, 2 mM EDTA, 1% Triton X-100, 0.1% SDS]; high salt wash buffer [20 mM Tris-HCl (pH 8.1), 500 mM NaCl, 2 mM EDTA, 1% Triton X-100, 0.1% SDS]; LiCl wash buffer [10 mM Tris-HCl (pH 8.1), 1 mM EDTA, 1% sodium deoxycholate, 1% NP-40, 0.25 mM LiCl]. Finally, the beads were washed twice with 1 ml TE buffer [1 mM EDTA, 10 mM Tris–HCl (pH 8.0)] for 5 min at 4°C. The immuno-complexes were then eluted in 120 µl elution buffer [1% SDS, 100 mM NaHCO3] for 15 min with rotation at room temperature. Reverse cross-linking was done by adding NaCl to the final concentration of 200 mM to ChIP and input samples and incubating at 65°C for 6 hrs. This followed by treatment with RNAase and proteinase K (40 µg/ml) incubating for 1 hourat 45°C. DNA was purified using QIAquick PCR Purification Kit (Qiagen), and 50 ng of eluted DNA was used for each qPCR reaction with primers listed in Table S10 to quantify relative ChIP signal.

Figure S1

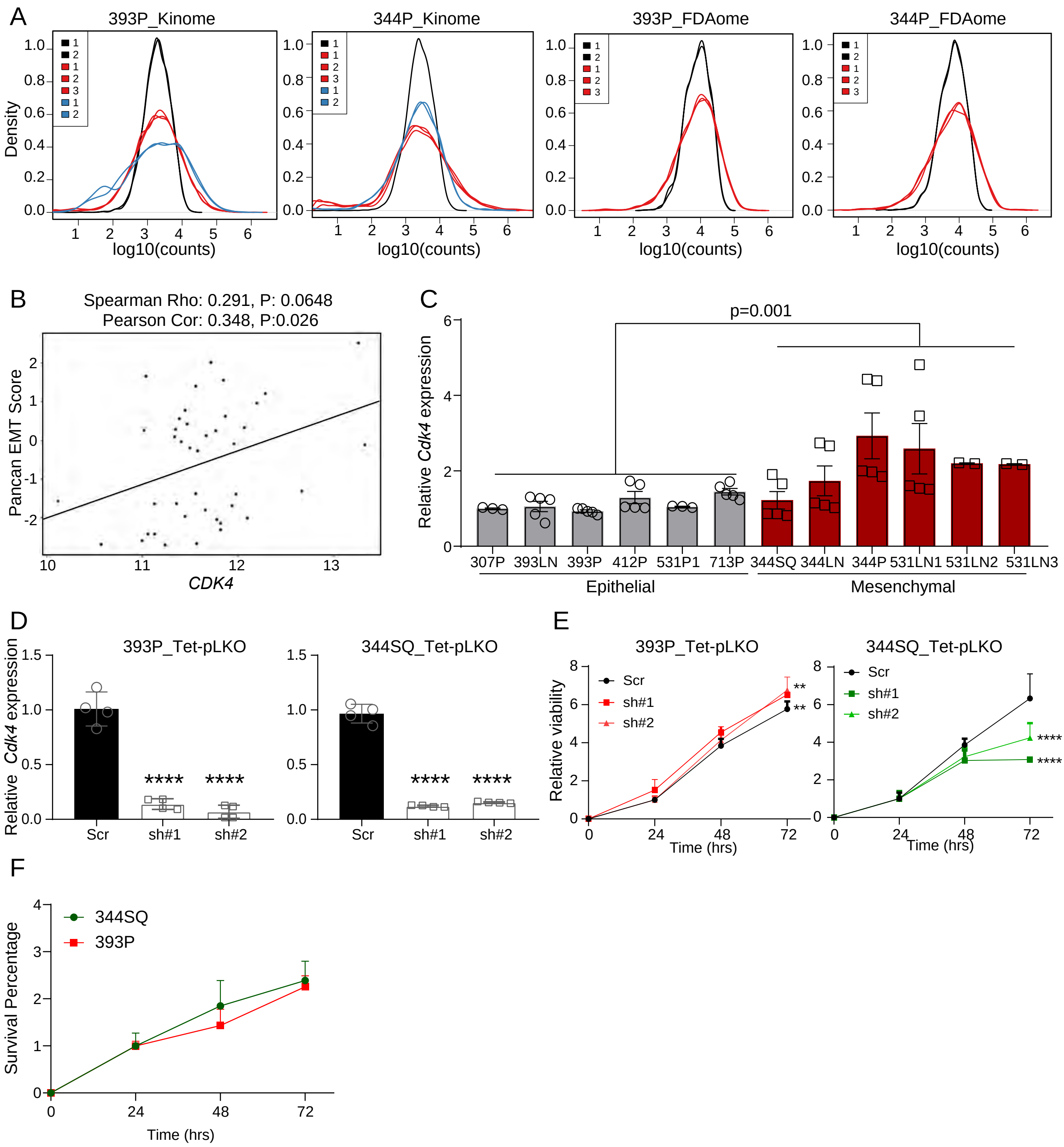
A
393P_Kinome
344P_Kinome
393P_FDAome
344P_FDAome
Density
log10(counts)
B
Spearman Rho: 0.291, P: 0.0648
Pearson Cor: 0.348, P:0.026
Pancan EMT Score
CDK4
C
p=0.001
Relative Cdk4 expression
307P
393LN
393P
412P
531P1
713P
344SQ
344LN
344P
531LN1
531LN2
531LN3
Epithelial
Mesenchymal
D
393P_Tet-pLKO
344SQ_Tet-pLKO
Relative Cdk4 expression
Scr
sh#1
sh#2
****
E
393P_Tet-pLKO
344SQ_Tet-pLKO
Relative viability
Scr
sh#1
sh#2
**
****
Time (hrs)
F
344SQ
393P
Survival Percentage
Time (hrs)

Figure S1: Mesenchymal lung cancer cells exhibit increased dependency on CDK4 for growth. (A) Viral integration distribution of reference population, cell lines and tumors determined through barcode sequencing (counts per million). Black = reference, red = in vivo samples, blue = in vitro samples. (B) Cluster plot analysis of correlation between EMT score and *CDK4* mRNA expression of 41 human KRAS mutant NSCLC cell lines. (C) Relative *CDK4* mRNA expression in murine epithelial and mesenchymal cells. Data is represented as mean $\pm$ SEM. Unpaired t-test was used for statistical analysis. (D) Relative *Cdk4* mRNA expression upon doxycycline mediated knockdown of *Cdk4* for 6 days in 393P and 344SQ cells. Data is represented as mean $\pm$ SD. One and two-way ANOVA test was used for statistical analysis. (E) Cell viability of 393P and 344SQ cells with *Cdk4* knockdown over 72 hours measured by MTT assay. Data is represented as mean $\pm$ SD. Two-way ANOVA test was used for statistical analysis. (F) Growth rates of 393P and 344SQ cells over 72 hours as measured by WST-1 assay. Data is represented as mean $\pm$ SD.

Figure S2

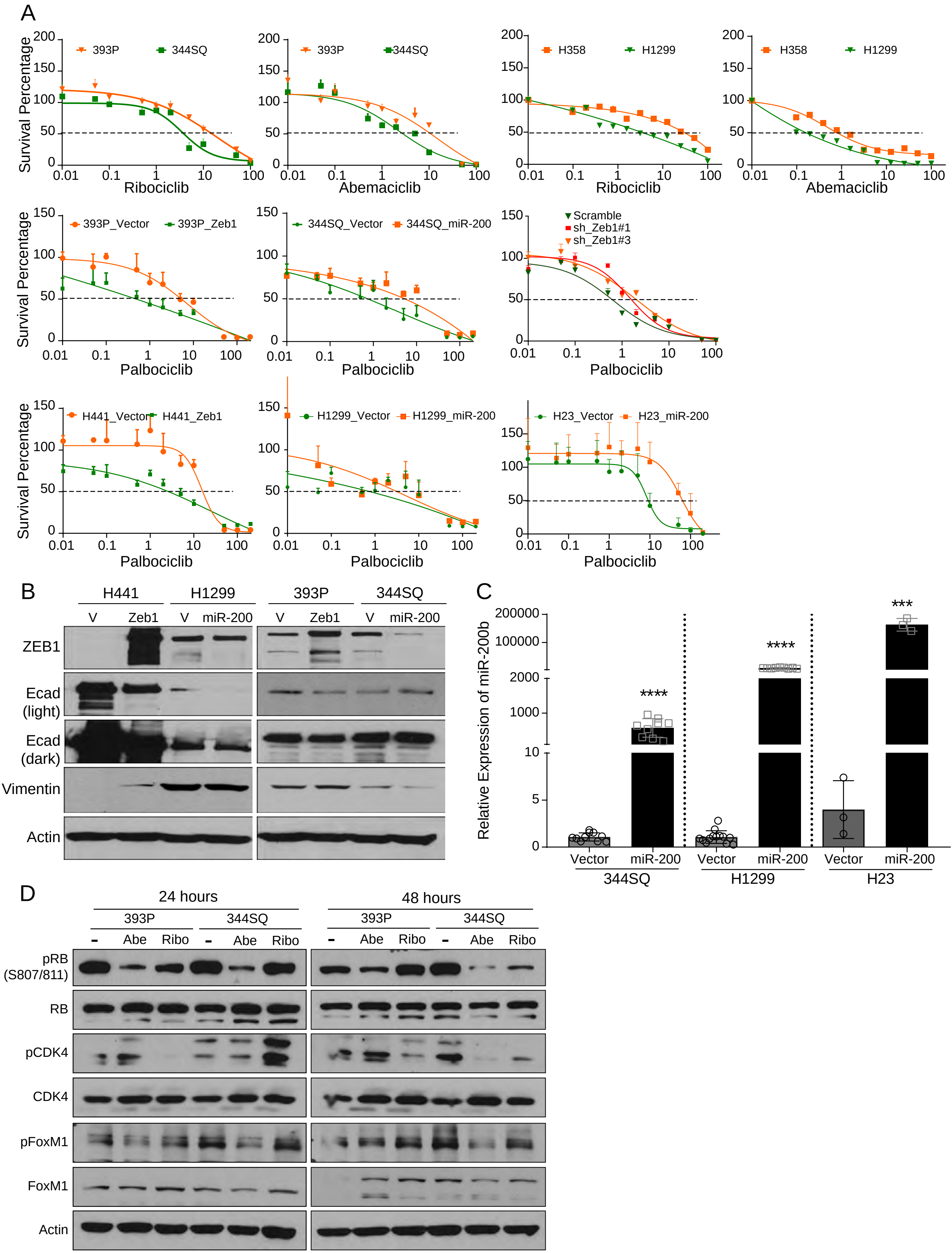

A
Survival Percentage
393P
344SQ
Ribociclib
Abemaciclib
H358
H1299
393P_Vector
393P_Zeb1
344SQ_Vector
344SQ_miR-200
Scramble
sh_Zeb1#1
sh_Zeb1#3
Palbociclib
H441_Vector
H441_Zeb1
H1299_Vector
H1299_miR-200
H23_Vector
H23_miR-200
B
H441
H1299
393P
344SQ
V
Zeb1
miR-200
ZEB1
Ecad (light)
Ecad (dark)
Vimentin
Actin
C
Relative Expression of miR-200b
****
****
***
Vector
miR-200
344SQ
H1299
H23
D
24 hours
48 hours
393P
344SQ
-
Abe
Ribo
pRB (S807/811)
RB
pCDK4
CDK4
pFoxM1
FoxM1
Actin

Figure S2: Mesenchymal lung cancer cells exhibit increased dependency on CDK4 for growth. (A) In vitro cell survival response after 48 hour abemaciclib, ribociclib or palbociclib treatment in a panel of murine and human lung cancer cell lines. n=8 per each drug concentration. The curve was generated using a nonlinear regression fit model. (B) Western blot analysis showing EMT and MET induction isogenic pairs of human and murine cell lines. (C) Relative expression of miR-200b in isogenic pairs of human and murine cell lines overexpressing miR-200. Data is represented as mean $\pm$ SD. Unpaired t-test was used for statistical analysis. **** $p<0.0001$; *** $p<0.005$ (D) Western blot showing the effect on signaling in 393P and 344SQ tumor cells upon treatment with abemaciclib (A: 2 µM) and ribociclib (R: 2 µM) for 24 and 48 hours.

Figure S3

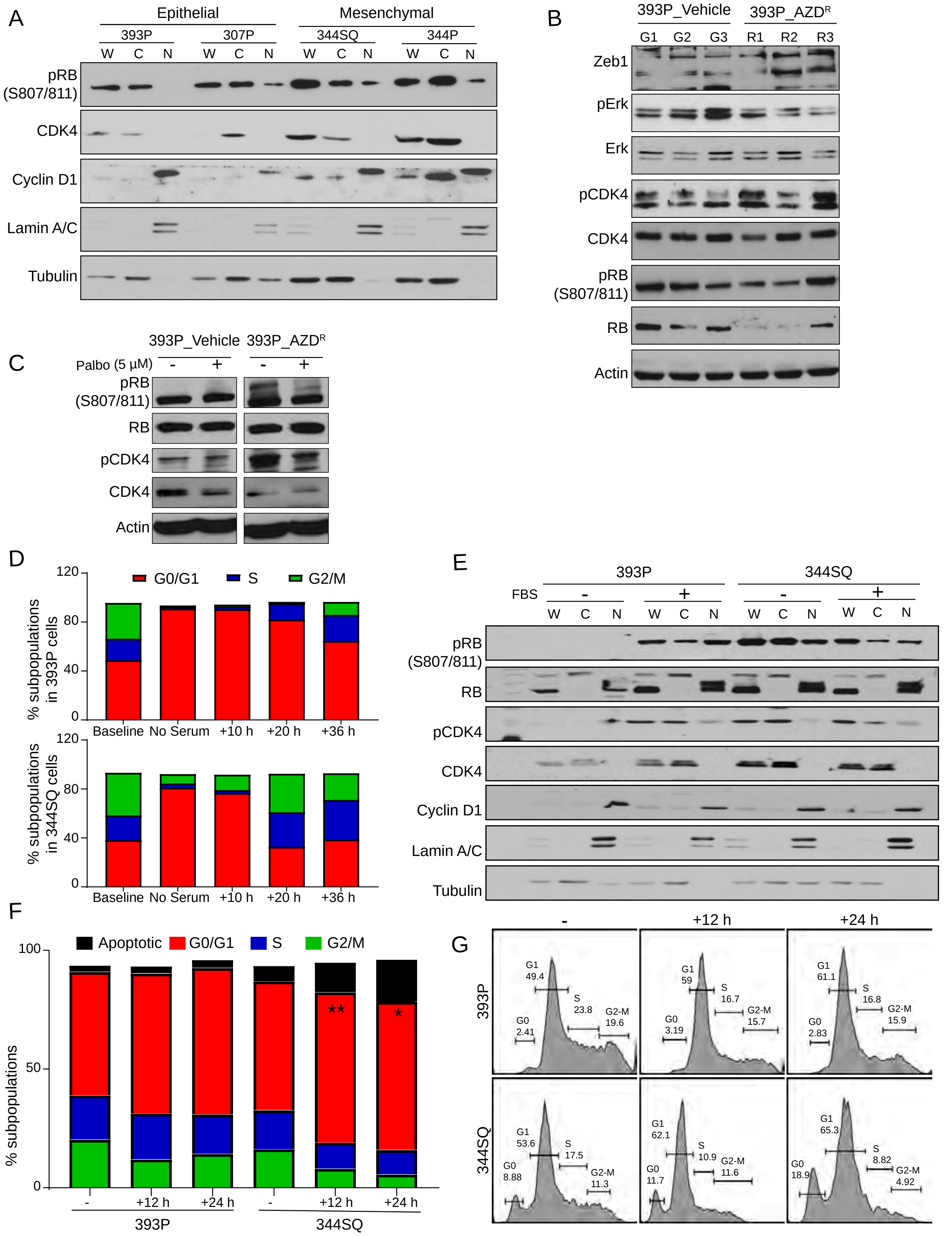
A
Epithelial
Mesenchymal
393P
307P
344SQ
344P
W C N W C N W C N W C N
pRB (S807/811)
CDK4
Cyclin D1
Lamin A/C
Tubulin
B
393P_Vehicle
393P_AZDR
G1 G2 G3 R1 R2 R3
Zeb1
pErk
Erk
pCDK4
CDK4
pRB (S807/811)
RB
Actin
C
393P_Vehicle 393P_AZDR
Palbo (5 µM) - + - +
pRB (S807/811)
RB
pCDK4
CDK4
Actin
D
G0/G1
S
G2/M
% subpopulations in 393P cells
% subpopulations in 344SQ cells
0
40
80
120
Baseline No Serum +10 h +20 h +36 h
E
393P
344SQ
FBS - + - +
W C N W C N W C N W C N
pRB (S807/811)
RB
pCDK4
CDK4
Cyclin D1
Lamin A/C
Tubulin
F
Apoptotic
G0/G1
S
G2/M
% subpopulations
0
50
100
**
*
- +12 h +24 h - +12 h +24 h
393P
344SQ
G
- +12 h +24 h
393P
344SQ
G1 49.4
S 23.8
G2-M 19.6
G0 2.41
G1 59
S 16.7
G2-M 15.7
G0 3.19
G1 61.1
S 16.8
G2-M 15.9
G0 2.83
G1 53.6
S 17.5
G2-M 11.3
G0 8.88
G1 62.1
S 10.9
G2-M 11.6
G0 11.7
G1 65.3
S 8.82
G2-M 4.92
G0 18.9

Figure S3: The CDK4 pathway is dynamically regulated by the EMT status of tumor cells. (A) Subcellular fractionation and western blot analysis of 393P, 307P and 344SQ, 344P cells for indicated markers. (B) Western blot analysis on cell lines derived from 393P tumors treated with vehicle or AZD6244 for indicated markers. (C) Western blot analysis of 393P_vehicle and 393P_AZD^{R} cells after treatment with palbociclib (5µM) for 48 hours for indicated markers. (D) Cell cycle analysis 393P and 344SQ cells using propidium iodide (PI). Baseline cell cycle was determined by staining cells in culture with PI. Cell cycle arrest was induced by serum starvation of cells for 24 hours followed by release into cell cycle by addition of FBS containing media and analyzed after 10, 20 and 36 hours. (E) Subcellular fractionation followed by western blot analysis of indicated cell cycle markers. (F) Cell cycle analysis 393P and 344SQ cells treated with palbociclib (5µM) for 12 and 24 hours using propidium iodide (PI). (G) Quantification of subpopulations in different cell cycle phase. Data are presented as mean $\pm$ SD and statistical analysis was done using one-way ANOVA test.* $p< 0.05$, ** $p< 0.01$.

Figure S4

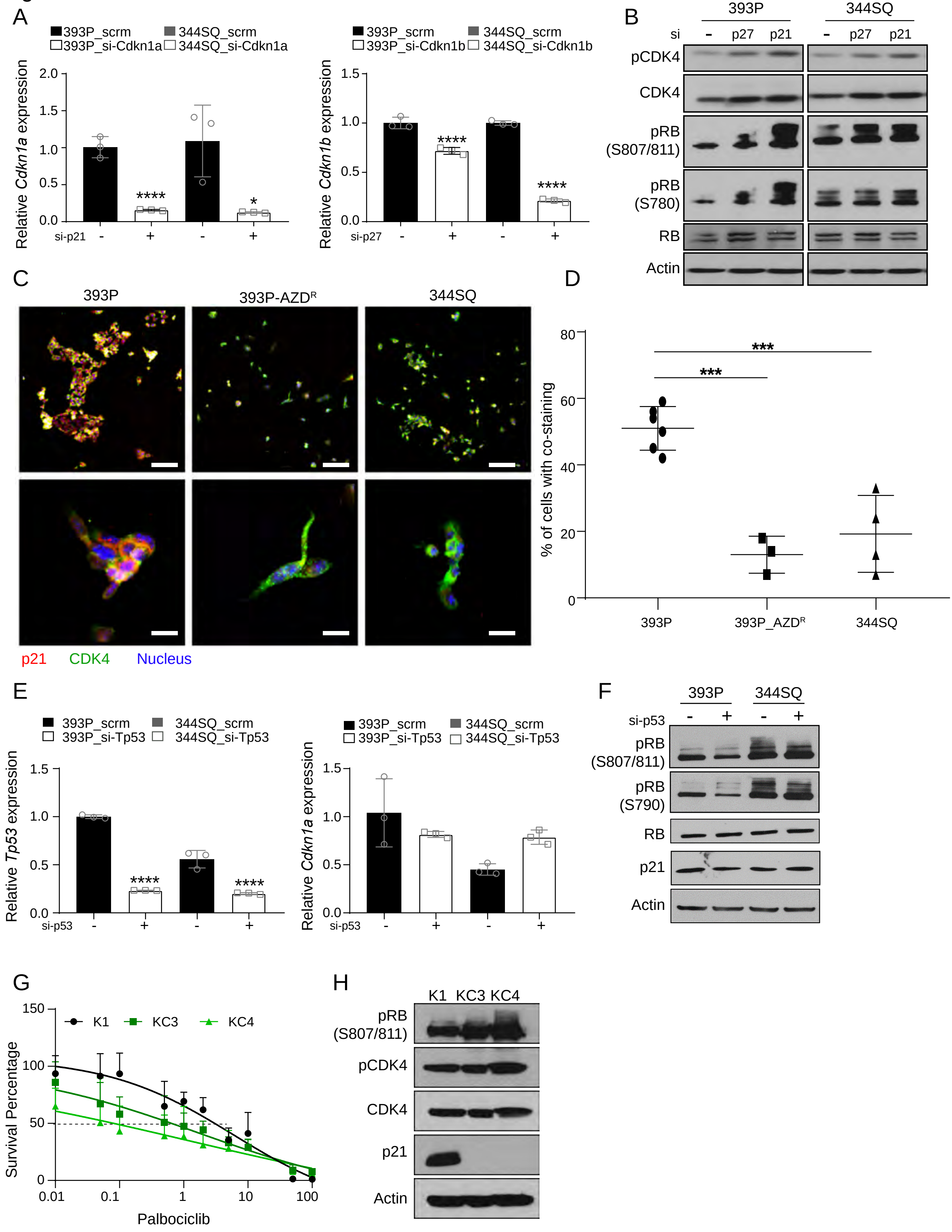
A
393P_scrm
344SQ_scrm
393P_si-Cdkn1a
344SQ_si-Cdkn1a
Relative Cdkn1a expression
2.0
1.5
1.0
0.5
0.0
****
*
si-p21 - + - +
393P_scrm
344SQ_scrm
393P_si-Cdkn1b
344SQ_si-Cdkn1b
Relative Cdkn1b expression
1.5
1.0
0.5
0.0
****
****
si-p27 - + - +
B
393P
344SQ
si - p27 p21 - p27 p21
pCDK4
CDK4
pRB (S807/811)
pRB (S780)
RB
Actin
C
393P
393P-AZDR
344SQ
p21
CDK4
Nucleus
D
% of cells with co-staining
80
60
40
20
0
***
***
393P
393P_AZDR
344SQ
E
393P_scrm
344SQ_scrm
393P_si-Tp53
344SQ_si-Tp53
Relative Tp53 expression
1.5
1.0
0.5
0.0
****
****
si-p53 - + - +
393P_scrm
344SQ_scrm
393P_si-Tp53
344SQ_si-Tp53
Relative Cdkn1a expression
1.5
1.0
0.5
0.0
si-p53 - + - +
F
393P
344SQ
si-p53 - + - +
pRB (S807/811)
pRB (S790)
RB
p21
Actin
G
150
100
50
0
K1
KC3
KC4
Survival Percentage
0.01
0.1
1
10
100
Palbociclib
H
K1 KC3 KC4
pRB (S807/811)
pCDK4
CDK4
p21
Actin

Figure S4: Differential p21 expression in epithelial and mesenchymal cells. (A) Transient knockdown of p21 and p27 using 20 nM si-RNAs for 48 hours in 393P and 344SQ cells. (B) Western blot analysis indicated markers after 48 hours of transient knockdown of p21 and p27 in 393P and 344SQ cells. (C) Immunofluroscent staining of 393P, 393P_AZD^R and 344SQ cells. Scale bar top: 100 µm, bottom: 10 µm. (D) Quantification of cells co-staining positive for CDK4 and p21 in each cell lines. 4-6 biological replicates were analyzed for quantification of the fluorescent signal. (E) Relative mRNA expression of *Tp53* and *Cdkn1a* with transient knockdown of *Tp53*. (F) Western blot analysis of indicated markers upon transient knockdown of *Tp53* for 48 hours. (G) In vitro cell viability after 48 hour palbociclib treatment of K1, KC3 and KC4 cell lines. n=8 per each drug concentration. The curve was generated using a nonlinear regression fit model. (H) Western blot analysis of indicated markers in K1, KC3 and KC4 cell lines. Data are presented as mean $\pm$ SD and unpaired t-test was used in all panels.

Figure S5

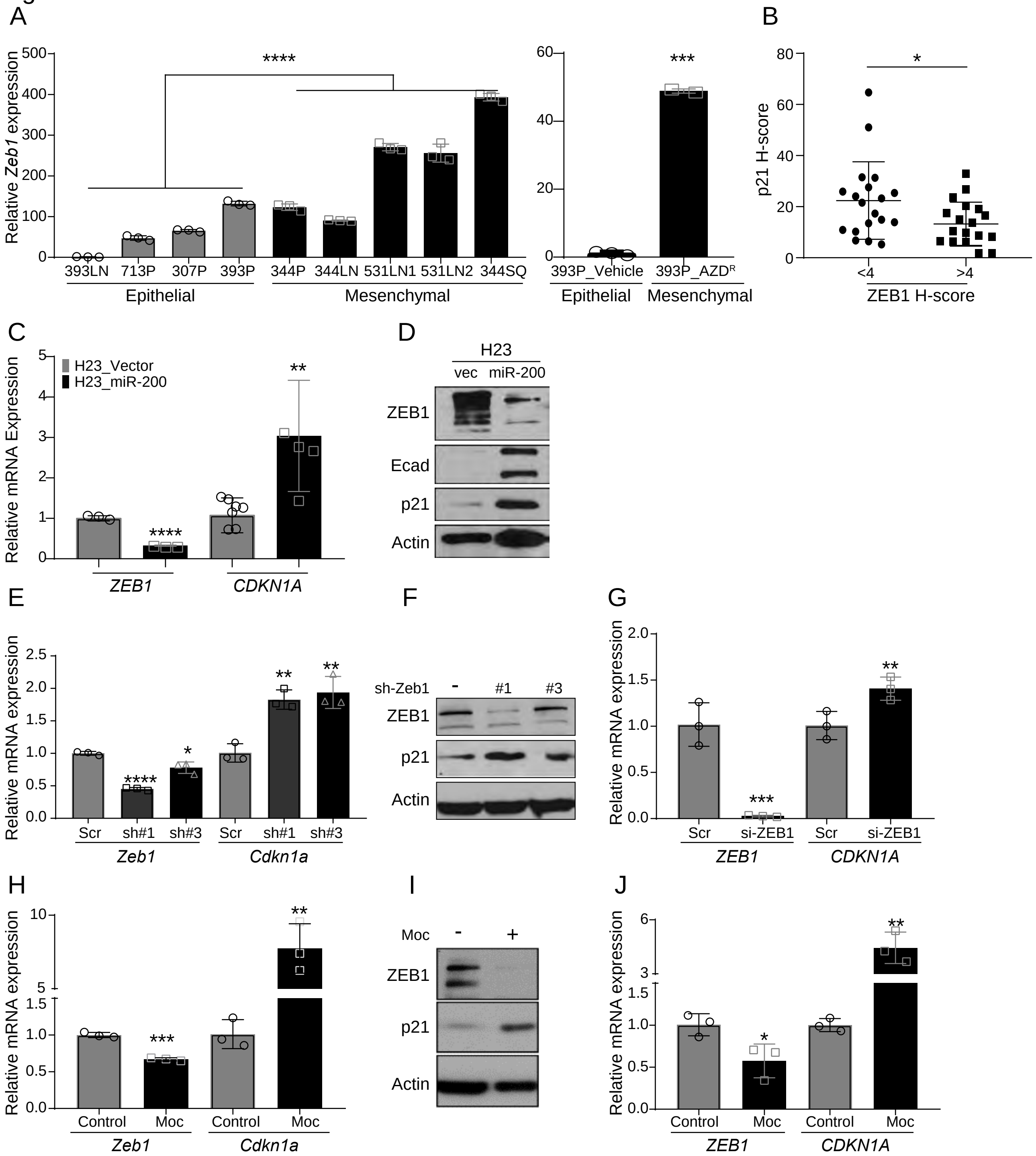
A
Relative Zeb1 expression
500
400
300
200
100
0
****
393LN
713P
307P
393P
344P
344LN
531LN1
531LN2
344SQ
Epithelial
Mesenchymal
60
40
20
0
***
393P_Vehicle
393P_AZDR
Epithelial
Mesenchymal
B
p21 H-score
80
60
40
20
0
*
<4
>4
ZEB1 H-score
C
Relative mRNA Expression
H23_Vector
H23_miR-200
5
4
3
2
1
0
**
****
ZEB1
CDKN1A
D
H23
vec
miR-200
ZEB1
Ecad
p21
Actin
E
Relative mRNA expression
2.5
2.0
1.5
1.0
0.5
0.0
****
*
**
**
Scr
sh#1
sh#3
Scr
sh#1
sh#3
Zeb1
Cdkn1a
F
sh-Zeb1
-
#1
#3
ZEB1
p21
Actin
G
Relative mRNA expression
2.0
1.5
1.0
0.5
0.0
***
**
Scr
si-ZEB1
Scr
si-ZEB1
ZEB1
CDKN1A
H
Relative mRNA expression
10
5
1.5
1.0
0.5
0.0
***
**
Control
Moc
Control
Moc
Zeb1
Cdkn1a
I
Moc
-
+
ZEB1
p21
Actin
J
Relative mRNA expression
6
3
1.5
1.0
0.5
0.0
*
**
Control
Moc
Control
Moc
ZEB1
CDKN1A

Figure S5: ZEB1 regulates p21 expression and causes differential CDK4 pathway activation. (A) Relative expression of *Zeb1* mRNA in a panel of murine lung cancer cells. (B) NSCLC specimens divided into two groups based on ZEB1 H-score and correlated with p21 H-score. (C) Relative mRNA expression of *ZEB1* and *CDKN1A* in H23 cells with miR-200 overexpression. (D) Western blot analysis in H23 cells with miR-200 overexpression for indicated markers. (E) Relative mRNA expression of indicated markers in 344SQ cells with stable *ZEB1* knockdown. (F) Western blot analysis of indicated markers in 344SQ cells with stable *ZEB1* knockdown. (G) Relative mRNA expression of indicated markers in H1299 cells with transient *ZEB1* knockdown. (H) Relative mRNA expression of indicated markers in 344SQ cells upon treatment with mocetinostat (1µM) for 48 hours.. (I) Western blot analysis of indicated markers in 344SQ cells upon treatment with mocetinostat (1µM) for 48 hours. (J) Relative mRNA expression of indicated markers in H1299 cells upon treatment with mocetinostat (1µM) for 48 hours. One-way ANOVA was used for statistical analysis. Data is represented as mean $\pm$ SD. Statistical analysis for all panels unpaired t-test. **** $p<0.0001$; *** $p<0.005$; ** $p<0.001$; * p,< 0.05.

Figure S6

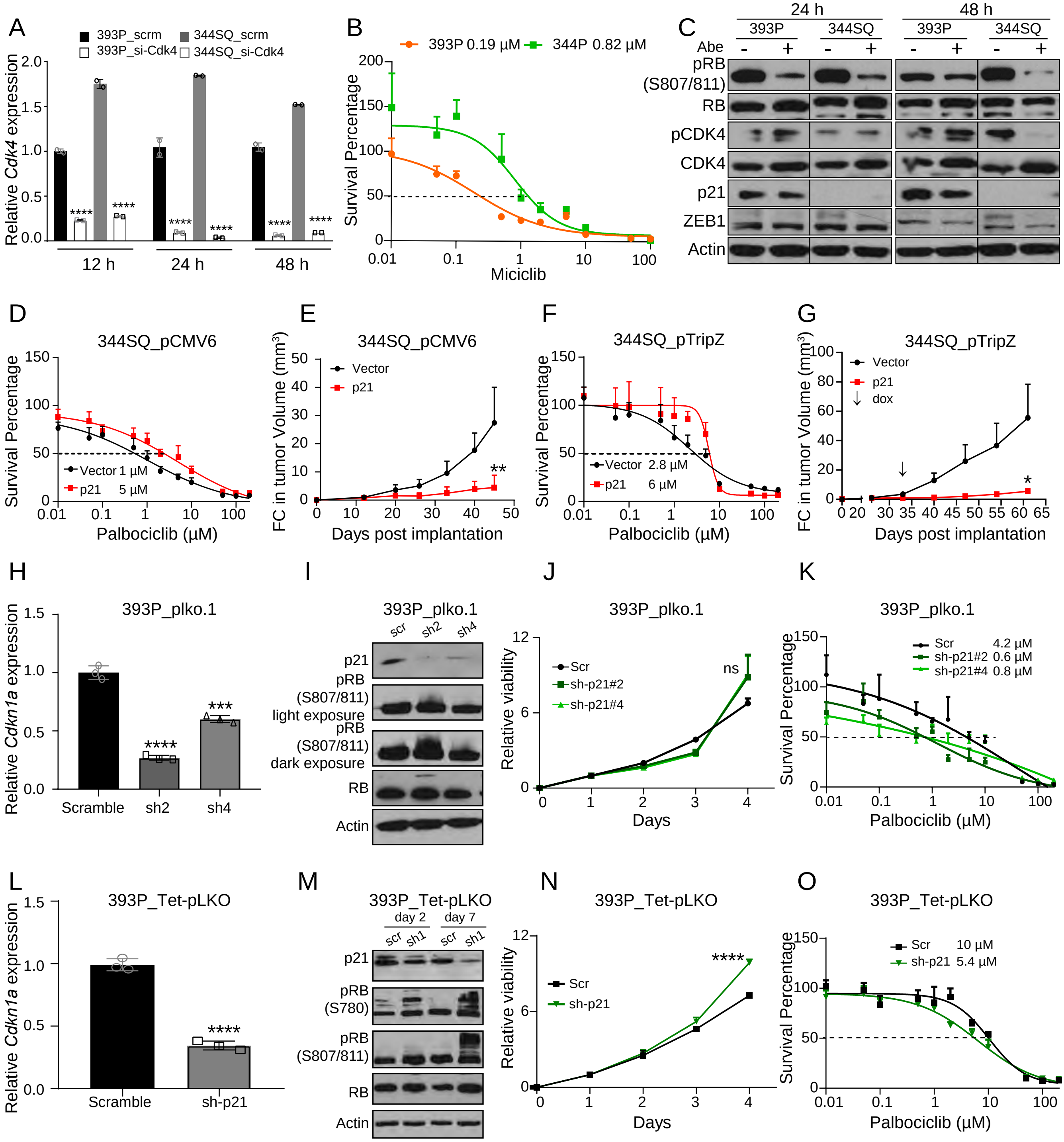
A
393P_scrm
344SQ_scrm
393P_si-Cdk4
344SQ_si-Cdk4
Relative Cdk4 expression
2.0
1.5
1.0
0.5
0.0
****
12 h
24 h
48 h
B
393P 0.19 µM
344P 0.82 µM
Survival Percentage
200
150
100
50
0
0.01
0.1
1
10
100
Miciclib
C
24 h
48 h
393P
344SQ
Abe
-
+
pRB (S807/811)
RB
pCDK4
CDK4
p21
ZEB1
Actin
D
344SQ_pCMV6
Survival Percentage
Vector 1 µM
p21 5 µM
Palbociclib (µM)
E
344SQ_pCMV6
FC in tumor Volume (mm³)
Vector
p21
**
Days post implantation
F
344SQ_pTripZ
Survival Percentage
Vector 2.8 µM
p21 6 µM
Palbociclib (µM)
G
344SQ_pTripZ
FC in tumor Volume (mm³)
Vector
p21
dox
*
Days post implantation
H
393P_plko.1
Relative Cdkn1a expression
****
***
Scramble
sh2
sh4
I
393P_plko.1
scr
sh2
sh4
p21
pRB (S807/811) light exposure
pRB (S807/811) dark exposure
RB
Actin
J
393P_plko.1
Relative viability
Scr
sh-p21#2
sh-p21#4
ns
Days
K
393P_plko.1
Survival Percentage
Scr 4.2 µM
sh-p21#2 0.6 µM
sh-p21#4 0.8 µM
Palbociclib (µM)
L
393P_Tet-pLKO
Relative Cdkn1a expression
****
Scramble
sh-p21
M
393P_Tet-pLKO
day 2
day 7
scr
sh1
p21
pRB (S780)
pRB (S807/811)
RB
Actin
N
393P_Tet-pLKO
Relative viability
Scr
sh-p21
****
Days
O
393P_Tet-pLKO
Survival Percentage
Scr 10 µM
sh-p21 5.4 µM
Palbociclib (µM)

Figure S6: Suppression of p21 in mesenchymal cells regulates CDK4 pathway. (A) Relative *CDK4* mRNA expression after transient knockdown using 20 nM si-RNA. (B) Cell viability after 48 hour miciclib treatment in 393P and 344P cells. (C) Western blot analysis of CDK4 pathway after treatment with 2 µM of abemaciclib at indicated times. (D) Cell viability after 48 hours of palbociclib treatment in 344SQ cells ± p21. (E) Fold change in tumor volume with constitutive p21 overexpression in 344SQ cells. Data are presented as mean ± SEM. (F) Cell viability after 48 hour palbociclib treatment in 344SQ cells ± p21 with doxycycline induction. (G) Fold change in tumor volume with p21 overexpression in 344SQ cells with doxycycline feed. Doxycycline feed was started after tumors reached a size of 100-150 mm^3 (indicated by arrow). Data are presented as mean ± SEM. (H) Relative mRNA *Cdkn1a* expression in 393P cells with stable knockdown using shRNA for *Cdkn1a.* (I) Western blot analysis for CDK4 pathway in response to *Cdkn1a* knockdown. (J) Growth rates of 393P cells ± p21 knockdown over 4 days measured by WST-1 assay. (K) Cell viability after 48 hours of palbociclib treatment in 393P cells with *Cdkn1a* knockdown. (L) Relative mRNA expression of *Cdkn1a* in 393P cells with knockdown using doxycycline inducible shRNA for *Cdkn1a* for 48 hours. (M) Western blot analysis for CDK4 pathway in 393P in response to *Cdkn1a* knockdown using doxycycline inducible shRNA for *Cdkn1a* for 2 and 7 days. (N) Growth rates of 393P cells ± p21 knockdown with doxycycline induction of shRNA over 4 days measured by WST-1 assay. (O) Cell viability after 48 hours of palbociclib treatment in 393P cells with *Cdkn1a* knockdown with doxycycline induced shRNA. Cell viability curves in *B, D, F,K, O* were generated using a nonlinear regression fit model. Data are presented as mean ± SD. n=8 per each drug concentration. IC50 for each cell line and drug is listed in the graph. Statistical analysis for *A, H* and *L*: One-way ANOVA, *E* and *G:* Unpaired t-test and *J* and *N:* Two-way ANOVA. **** p<0.0001; *** p<0.005; ** p<0.001; * p< 0.05; ns: not significant.

Figure S7

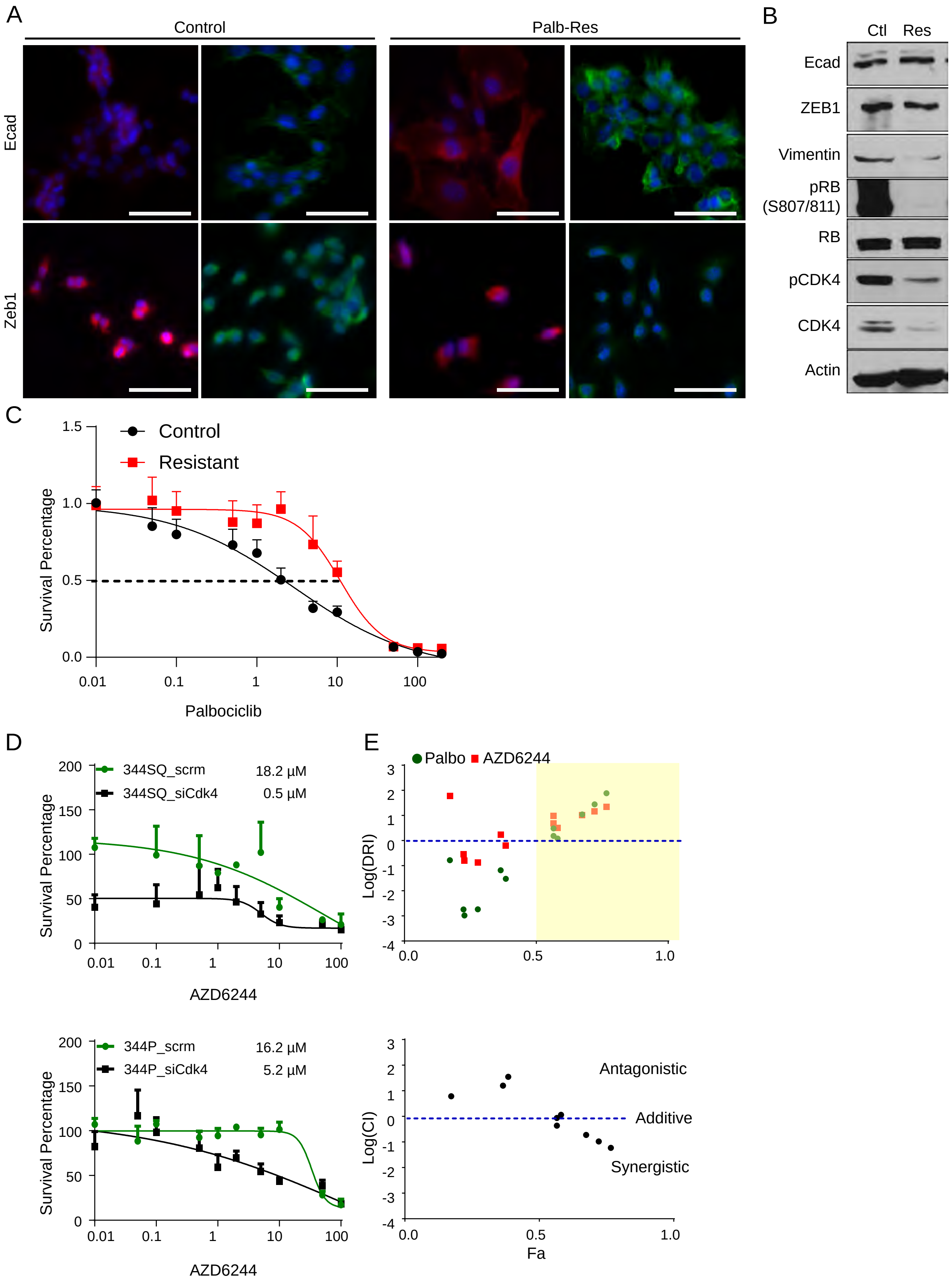

A
Control
Palb-Res
Ecad
Zeb1
B
Ctl
Res
Ecad
ZEB1
Vimentin
pRB (S807/811)
RB
pCDK4
CDK4
Actin
C
Control
Resistant
Survival Percentage
Palbociclib
D
344SQ_scrm 18.2 µM
344SQ_siCdk4 0.5 µM
Survival Percentage
AZD6244
344P_scrm 16.2 µM
344P_siCdk4 5.2 µM
Survival Percentage
AZD6244
E
Palbo
AZD6244
Log(DRI)
Log(CI)
Antagonistic
Additive
Synergistic
Fa

Figure S7: Palbociclib treatment induces MET in lung cancer cells. (A) Immunofluroscent staining in 344SQ control and palbociclib resistant cells for indicated markers. Blue: DAPI. Scale bar: 50 µm (B) Western blot analysis of indicated markers in 344SQ control and palbociclib resistant cells. (C) Cell viability after 48 hours of palbociclib treatment in 344SQ control and palbociclib resistant cells. Cell viability curves were generated using a nonlinear regression fit model. Data are presented as mean ± SD. n=8 per each drug concentration. (D) In vitro cell viability assay on 344SQ (top) and 344P (bottom) lung cancer cell lines with or without transient knockdown of CDK4 and treatment with a range of concentrations of AZD6244 for 48 hours. n=8 per drug concentration. The curve was generated using a nonlinear regression fit model. (E) Palbociclib and AZD6244 were used to determine the Drug Reduction Index (DRI) and Combination Index (CI) using Chou-Talalay method on 344SQ cells.

Figure S8

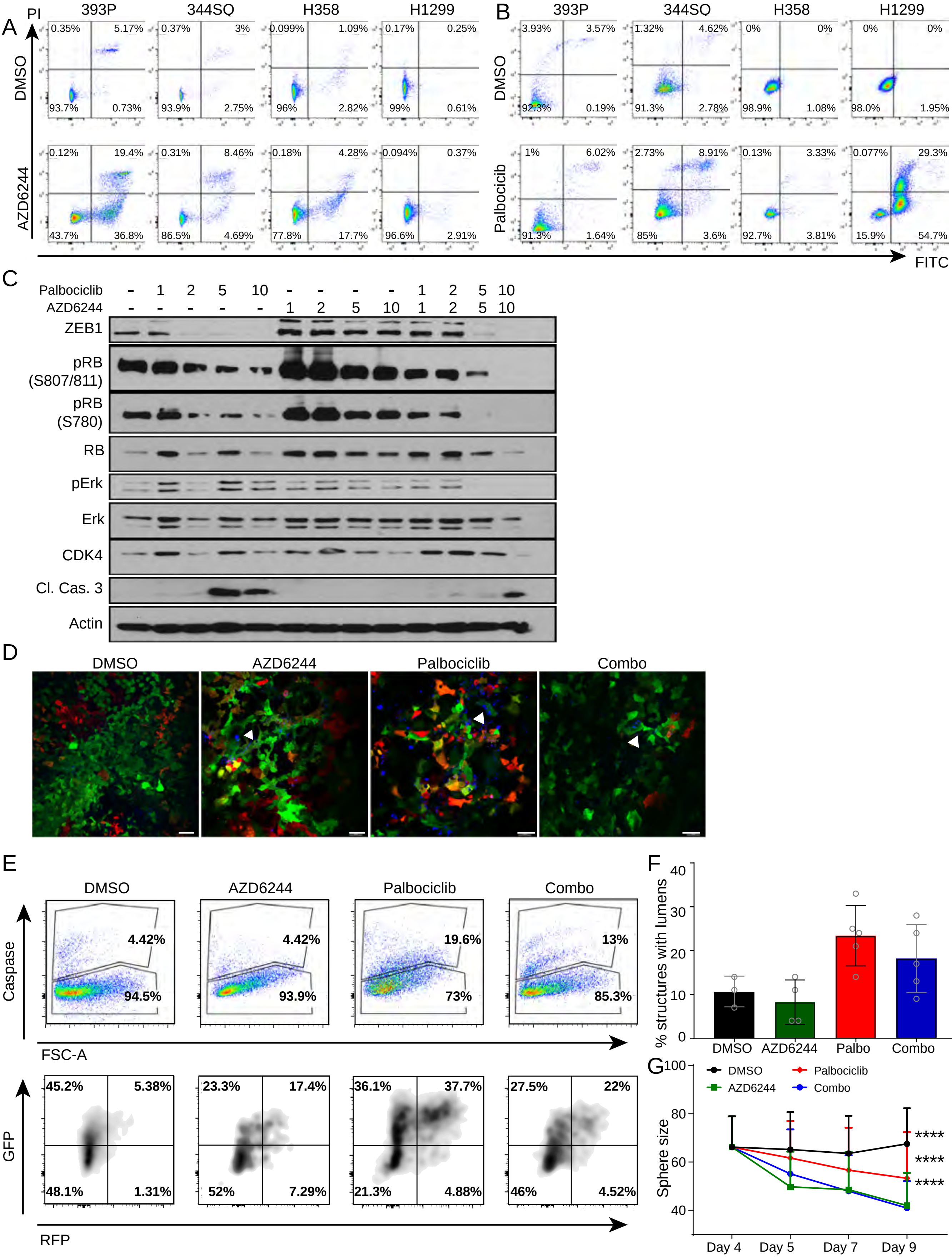
A
B
C
D
E
F
G
393P
344SQ
H358
H1299
PI
FITC
DMSO
AZD6244
Palbociclib
Combo
ZEB1
pRB (S807/811)
pRB (S780)
RB
pErk
Erk
CDK4
Cl. Cas. 3
Actin
Caspase
FSC-A
GFP
RFP
% structures with lumens
DMSO
AZD6244
Palbo
Combo
Sphere size
Day 4
Day 5
Day 7
Day 9
****

Figure S8: Co-targeting CDK4 and MAPK pathways targets different tumor cell subsets. (A-B) Representative plots from one set of annexin V and PI staining experiments performed in triplicate in 5A are shown. Early apoptotic cells (annexin V+ and PI-) are shown in the lower right quadrant, and late apoptotic cells (annexin V+ and PI+) are shown in the upper right quadrant. (C) Western blot analysis of 344SQ_Z-cad cells treated with increasing concentrations of palbociclib, AZD6244 or both. (D) 344SQ_Z-cad cells treated with DMSO, AZD6244 (5 μM), palbociclib (5 μM) or the combination for 48 hours. NucView® 405 Caspase 3 substrate was added to each condition as a readout for apoptosis. Representative fluorescent images were acquired 48 hours after addition of drugs (left). Scale bar = 50 μm. Arrows indicate apoptotic cells. (E) 344SQ_Z-cad cells treated with indicated drugs were stained with NucView® 405 Caspase 3 substrate and analyzed by flow cytometry. Representative plots are shown. (F) EVTs cultured in MG and treated with drugs indicated were scored for percentage of structures with central lumens. n=5 FOVs. Treatment groups were compared to DMSO using one-way ANOVA. (G) Quantification of size of EVTs from 5F at indicated times after plating in MG and treatment with indicated drugs. One-way ANOVA was used to determine statistical significance. **** $p<0.0001$; * $p< 0.05$.

Figure S9

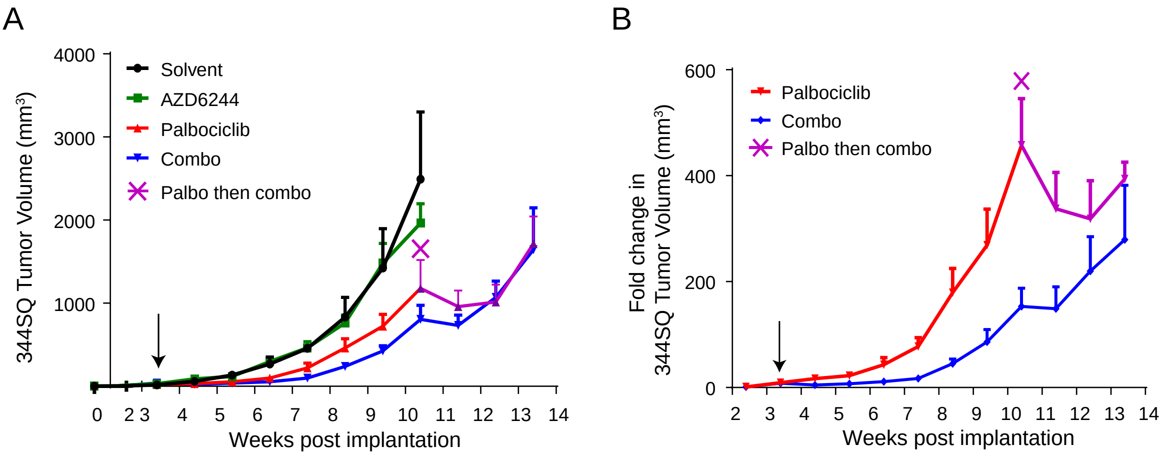

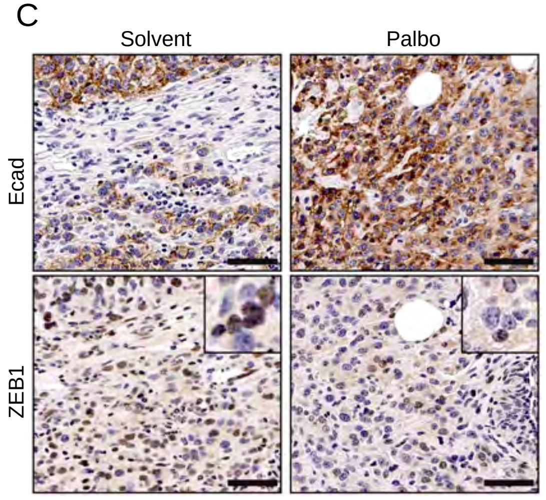

D

Model	Combo EAR	Combo actual	Bliss effect
344SQ_short term	383.4	350.9	Additive
344SQ_long term	342.8	237.4	Additive
393P	378.0	178.4	Synergistic

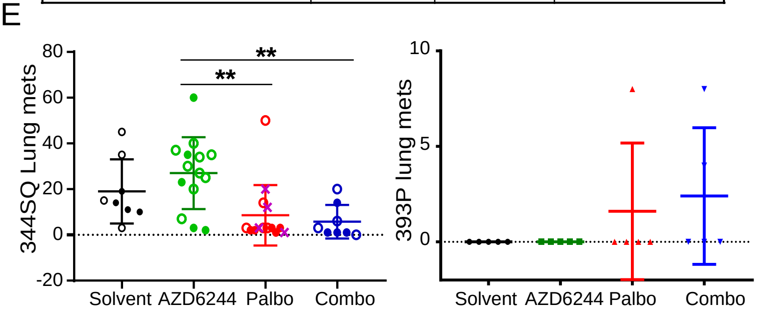

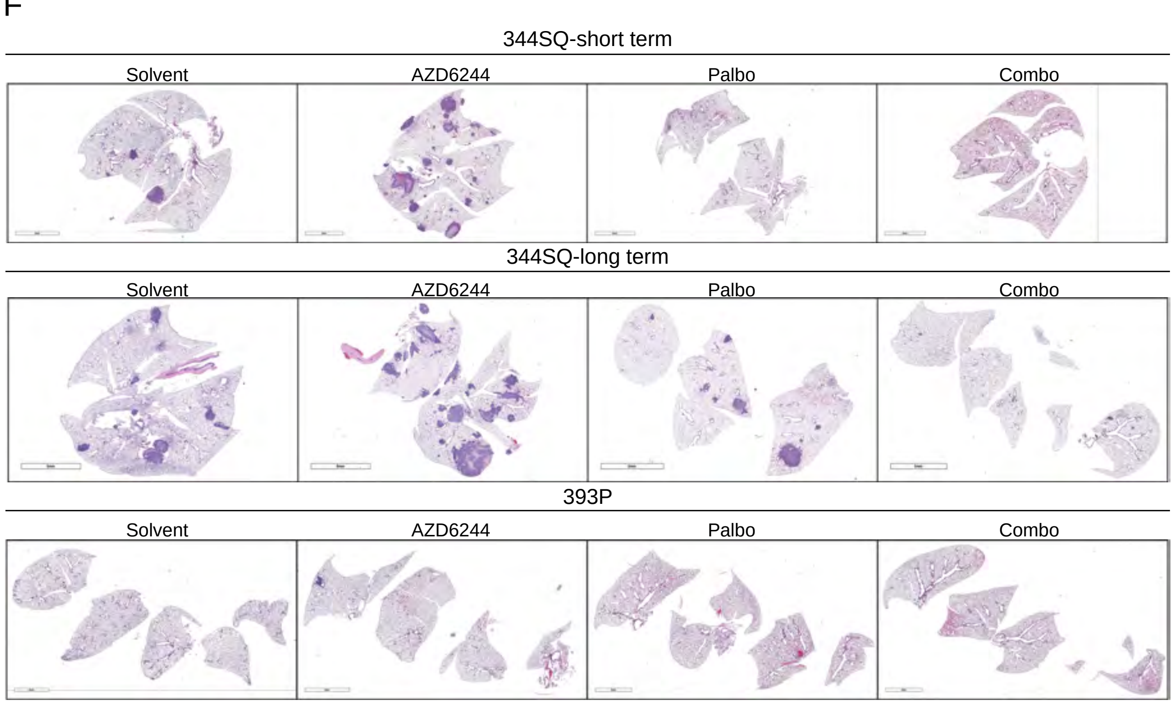

Figure S9: Combination of MEK and CDK4 inhibitors controls syngeneic tumor growth and prevents emergence of EMT mediated resistance. (A) 344SQ subcutaneous tumors were treated with solvent (n=5), AZD6244 (25 mg/kg) (n=10), palbociclib (50 mg/kg) (n=10) or combination (n=5) for 7 weeks at which point resistance to palbociclib emerged. 5 mice from palbociclib treatment alone arm were converted to combination arm and treatment was continued for another 3 weeks (marked by purple X). (B) Fold change in 344SQ tumors in the palbociclib and combo arms over 13 weeks. (C) Immunohistochemical analysis on 344SQ tumors treated with solvent or palbociclib for 7 weeks with indicated markers. Scale bar: 50 µm. (D) Summary of Bliss effect analysis of the in vivo studies (5A, 5B, S9A and S9B). The mean of expected additive tumor volumes with combination (combo EAR) treatment were calculated by Bliss independent method. The actual tumor volumes with combination (combo actual) treatment lower than the expected additive tumor volumes are considered as synergistic. (E) At necropsy, mouse lungs from the experiment described in (5A, 5B, S9A and S9B) were analyzed for macroscopic metastatic lung nodules in each treatment group. 344SQ tumors: Black solid circles: short term solvent, black open circles: long term solvent. Green solid circles: short term AZD6244, green open circles: long term palbociclib, purple crosses: palbo then combo. Blue solid circles: short term combo, blue open circles: long term combo. (F) Hematoxylin and eosin (H&E) staining of whole lung sections. Representative images of lungs harvested in (E). Data are presented as mean ± SD. Statistical significance was determined by one-way ANOVA.

Figure S10

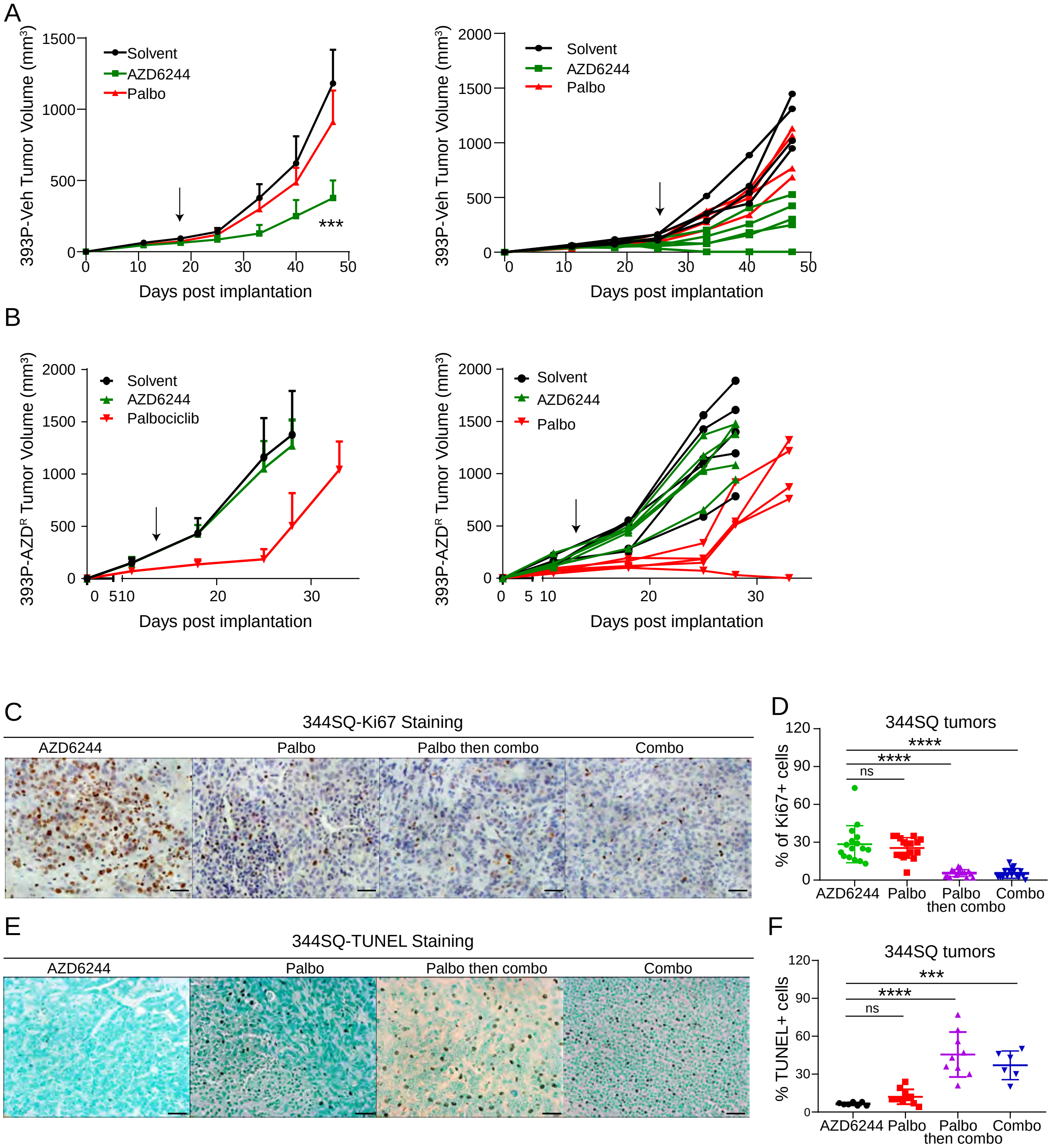
A
393P-Veh Tumor Volume (mm³)
Solvent
AZD6244
Palbo
***
Days post implantation
B
393P-AZD^R Tumor Volume (mm³)
Palbociclib
C
344SQ-Ki67 Staining
AZD6244
Palbo
Palbo then combo
Combo
D
344SQ tumors
% of Ki67+ cells
ns
****
E
344SQ-TUNEL Staining
F
% TUNEL+ cells
then combo

Figure S10: Combination of MEK and CDK4 inhibitors controls syngeneic tumor growth and prevents emergence of EMT mediated resistance. (A) In vivo volume measurements of 393P-vehicle subcutaneous tumors in syngeneic WT mice (n=5 per group) after daily treatment with AZD6244 (25 mg/kg) or palbociclib (50 mg/kg). Arrow indicates start of treatment at day 20. Individual growth curves of tumors (right). (B) In vivo volume measurements of 393P-AZD^R subcutaneous tumors in syngeneic WT mice (n=5 per group) after daily treatment with AZD6244 (25 mg/kg) or palbociclib (50 mg/kg). Arrow indicates start of treatment at day 14. Individual growth curves of tumors (right). (C-F) 344SQ tumors from the long term experiments described in (S9A) were stained with Ki67 and TUNEL assay to measure cell proliferation and cell death respectively. Representative IHC images are shown in C and E. Scale bar: 50 μm. Images were quantified for Ki67 (D) and TUNEL (F) staining in each treatment group. n=2-3 per group with 3-6 FOV per mouse. Data are presented as mean ± SD. Statistical significance of was determined by one-way ANOVA.

Figure S11

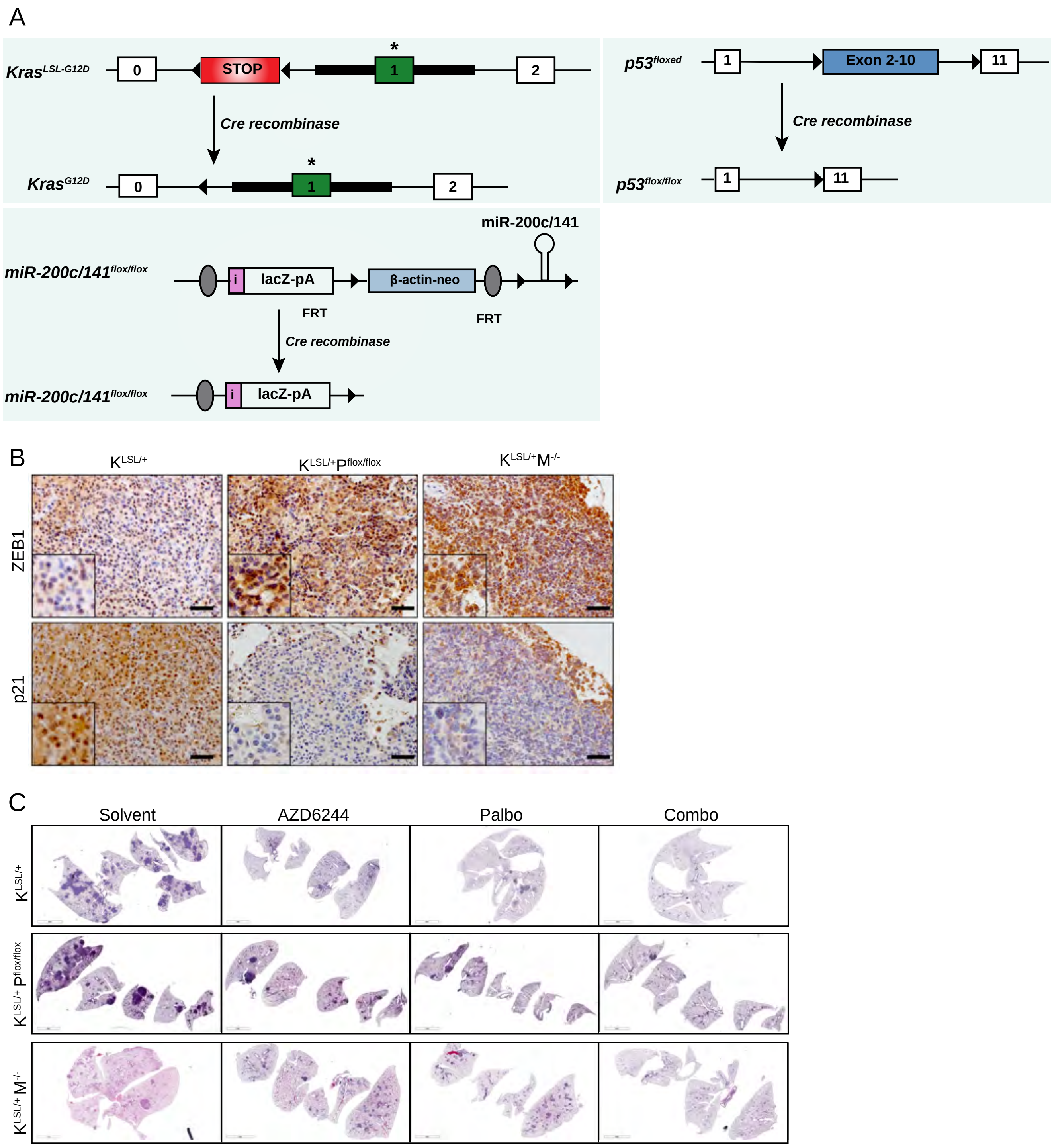
A
Kras LSL-G12D
0
STOP
*
1
2
Cre recombinase
Kras G12D
0
*
1
2
p53 floxed
1
Exon 2-10
11
Cre recombinase
p53 flox/flox
1
11
miR-200c/141
miR-200c/141 flox/flox
i
lacZ-pA
β-actin-neo
FRT
FRT
Cre recombinase
miR-200c/141 flox/flox
i
lacZ-pA
B
K LSL/+
K LSL/+ P flox/flox
K LSL/+ M -/-
ZEB1
p21
C
Solvent
AZD6244
Palbo
Combo
K LSL/+
K LSL/+ P flox/flox
K LSL/+ M -/-

Figure S11: Concomitant targeting of CDK4 and MAPK pathways augments response in Kras mutant autochthonous lung tumors. (A) Schematic representation of *Kras*$^{LSL/+}$*, Kras* $^{LSL/+}$*P*$^{flox/flox}$ and *Kras* $^{LSL/+}$*M*$^{-/-}$ before and after cre recombination. (B) Immunohistochemistry for indicated markers on *Kras*$^{LSL/+}$, *Kras* $^{LSL/+}$*P*$^{flox/flox}$ and *Kras* $^{LSL/+}$*M*$^{-/-}$ lung sections 18-20 weeks post Ad-Cre infection. Scale bar: 50 µm. (C) Hematoxylin and eosin (H&E) staining of whole lung section of *Kras*$^{LSL/+}$*, Kras* $^{LSL/+}$*P*$^{flox/flox}$ and *Kras* $^{LSL/+}$*M*$^{-/-}$ 18-20 weeks post Ad-Cre infection.

Figure S12

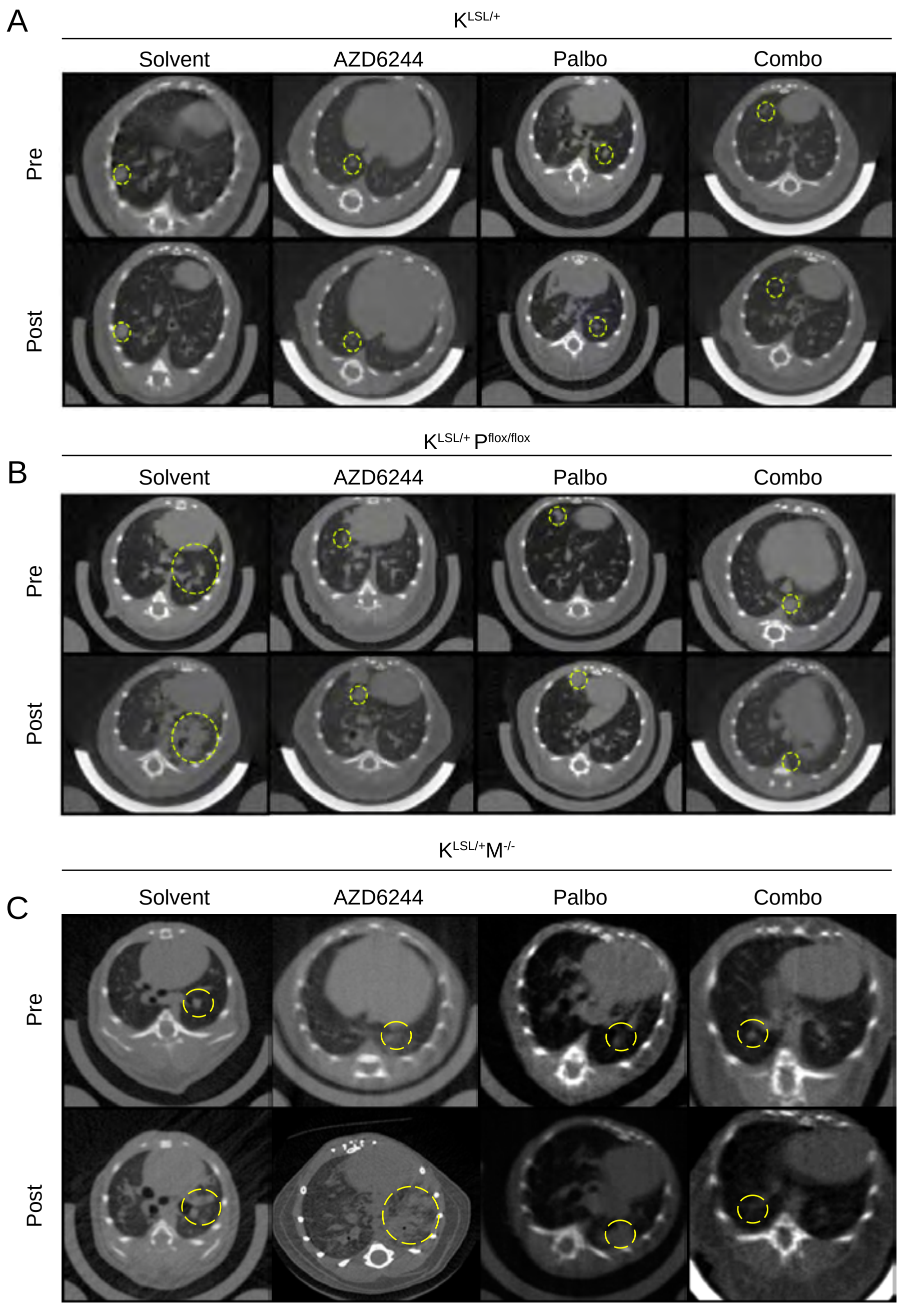

A
K^LSL/+
Solvent
AZD6244
Palbo
Combo
Pre
Post
B
K^LSL/+ P^flox/flox
Solvent
AZD6244
Palbo
Combo
Pre
Post
C
K^LSL/+M^-/-
Solvent
AZD6244
Palbo
Combo
Pre
Post

Figure S12: Concomitant targeting of CDK4 and MAPK pathways augments response in Kras mutant autochthonous lung tumors. (A) Representative cross-sectional micro-CT images of $Kras^{LSL/+}$, $Kras^{LSL/+}P^{flox/flox}$ and $Kras^{LSL/+}M^{-/-}$ mouse lungs before and after treatment administration (6-8 weeks). Yellow circles outline representative target lesions.

Table S1. Quality control metrics for the Kinome shRNA screens

	393P					344P				
	in-vivo			in-vitro		in-vivo			in-vitro	
reagent	1	2	3	1	2	1	2	3	1	2
LUC_10041	1.38	0.39	-0.02	3.72	2.88	0.31	1.16	4.12	0.24	0.74
LUC_10042	-0.28	2.36	3.94	-1.22	2.9	-0.61	-6.96	0.67	0.88	0.41
LUC_10043	-0.02	0.09	-0.99	1.86	3.15	0.71	1.05	0.8	0.41	0.17
LUC_10044	2.85	0.71	1.06	1.09	4.05	-0.32	-0.01	1.59	0.84	1.51
LUC_10045	0.42	0.07	0.49	1.03	1.27	1.4	4.17	0	0.55	0.49
LUC_10046	-0.57	-1.06	-0.2	-1.01	-4.08	0.16	3.94	5.75	-0.38	1.14
LUC_10047	-0.34	0.06	0.21	-1.19	-2.85	0.45	1.81	2.79	3.95	3.98
LUC_10048	2.32	2.82	0.34	3.24	2.22	-0.12	3.86	1.17	1.13	0.68
LUC_10049	0.58	0.97	1.98	0.9	2.21	1.72	1.99	0.18	0.67	0.49
LUC_10050	-1.72	-0.38	-1.06	-1.63	1.06	-11.82	-0.14	-5.76	0.4	0.08
LUC_10051	0.73	4.01	1.21	3.5	2.2	-0.06	0.55	-0.66	-0.11	0.5
LUC_10052	3.63	-1.99	0.52	3.37	1.95	-0.58	1.84	-1.68	0.97	0.87
LUC_10053	0.36	0.7	2.6	3.35	3.99	0.59	-0.62	-9.14	0.95	0.16
LUC_10054	-1.47	0.69	-0.44	-1.81	3.4	-1.25	0.53	-12.03	0.11	0.23
LUC_10055	-1.3	-0.68	-0.49	-0.44	-0.99	0.16	0.59	0.24	1.81	1.67
LUC_10056	-2.53	-3.06	-0.09	-0.69	-1.69	-11.55	-11.55	-11.55	-1.36	-2.68
LUC_10057	1.28	0.7	-1.12	0.07	0.75	0.36	0.42	-0.87	0.34	0.39
LUC_10058	-0.74	-10.09	-2.44	-4.1	-0.78	0.43	0.29	-10.58	-6.65	-10.58
LUC_10059	-0.02	-1.96	-1.36	-4.1	-4.93	0.72	-12.24	-0.3	-3.95	-4.25
LUC_10060	-1.89	-5.64	-2.07	-4.09	-4.19	-10.99	-10.99	-10.99	-10.99	-10.99
Rpl30_1000[illegible]	-0.99	1.45	-2.72	-4.68	-4.75	1.67	-1.7	-0.04	-1.73	-0.26
Rpl30_1000[illegible]	-2.81	-6.85	-2.84	-4.59	-5.92	-0.94	-11.57	-8.5	-1.04	-11.57
Rpl30_1000[illegible]	2.72	-0.86	-2.75	-1.42	0.24	-1.28	0.33	-2.12	-4.64	-2.6
Rpl30_1000[illegible]	-3.98	-1	-1.36	-0.69	-0.05	-8.09	-12.99	-12.99	-4.6	-5.72
Rpl30_1000[illegible]	-4.59	-4.45	-4.55	-3.42	-4.88	-1.08	-13.05	-13.05	-13.05	-13.05
Rpl30_1000[illegible]	0.14	-1.62	-2.69	-3.05	-1.94	-12.41	1.24	-12.41	-1.98	-5.41
Rpl30_1000[illegible]	-1.39	-1.88	-3.38	-2.45	-1.91	-1.46	-1.44	-14.32	-0.93	-3.42
Rpl30_1000[illegible]	-4.03	-4.86	-2.98	-3.95	-2.52	-1.42	-1.73	-2.76	-12.6	-5.04
Rpl30_1000[illegible]	-2.2	-2	-1.2	-2.46	2.67	-1.75	-1.17	-1.15	-2.98	-2.59
Rpl30_1001[illegible]	1.11	-3.03	-1.45	-4.62	-1.4	-11.93	-11.93	-7.51	-2.85	-7.36
Rpl30_1001[illegible]	-3.37	-0.88	-0.81	-1.58	-1.24	-3.91	-0.71	-2.21	-2.07	-3.95
Rpl30_1001[illegible]	-0.79	-10.23	-6.2	-4.48	-5.47	-0.37	-10.03	0.74	-10.03	-10.03
Rpl30_1001[illegible]	-2.1	-2.79	-7.91	-4.82	-4.16	-11.51	-11.51	-11.51	-2.57	1.55
Rpl30_1001[illegible]	-6.31	-1.31	-1.56	-6.23	-6.16	-1.26	-11.57	0.13	-4.59	-2.36
Rpl30_1001[illegible]	-4.73	-2.52	-5.41	-3.72	-4.28	-11.85	-11.85	-11.85	-0.82	-2.19
Rpl30_1001[illegible]	-2.42	-6.94	0.17	-4.55	-5.7	-12.57	-8.97	-0.58	-4.23	-5.01
Rpl30_1001[illegible]	-2.78	-2.13	-3.33	1.11	-0.56	-1.21	-8.07	0.48	-2.9	-5.83
Rpl30_1001[illegible]	-2.01	1.65	-6.21	-0.57	-0.82	-4.46	0.22	-10.87	-0.7	-7.47
Rpl30_1001[illegible]	-3.65	-6.19	-4.08	-4.56	-3.87	-1.21	-8.41	0.06	-2.81	-2.21
Rpl30_1002[illegible]	-4.31	-7.71	-2.93	0.3	-4.64	-10.75	-3.9	-10.75	-0.25	-10.75
Psma1_100[illegible]	0.48	1.8	0.46	-3.37	-2.48	0.97	2.85	3.32	0.9	1.26
Psma1_100[illegible]	0.05	0.54	0.16	-0.9	-3.02	1.72	5.68	1.73	1.07	0.74
Psma1_100[illegible]	0.67	1.09	0.55	-1.03	0.68	-1.27	0.63	0.97	1.45	1.84

Psma1_1002	-0.14	0.52	0.63	-1.45	-0.04	3.48	1.4	2.77	0.76	0.9
Psma1_1002	0.32	1.67	0.62	-0.46	-0.08	0.89	0.06	3.09	0.83	0.35
Psma1_1002	-2.71	0.77	-2.09	-5.09	-5.23	-2.62	-11.47	-11.47	-6.42	-6.66
Psma1_1002	-1.38	-1.87	-3.7	-4.41	-3.91	-0.26	1.44	-0.82	-2.51	-3.41
Psma1_1002	-1.6	-2.28	-3.82	-4.57	-2.51	-0.53	-1.47	-11.63	-11.63	-11.63
Psma1_1002	-2.94	-2.64	-1.95	-4.7	-1.94	-0.96	-0.17	-0.02	-4.07	-2.93
Psma1_1003	-5.53	-2.56	-5.78	-4.23	-4.39	-10.91	-10.91	-10.91	-10.91	-10.91
Psma1_1003	-4.04	2.34	-3.58	-2.61	-4.77	-1.23	-1.5	-13.16	-3.76	-3.89
Psma1_1003	-5.03	-3.91	-2.96	-4.66	-4.11	-1.4	-3.85	-1.65	-2.82	-2.18
Psma1_1003	-3.83	-3.54	-2.49	-2.58	-0.56	-3.57	6.82	-1.08	-3.53	-6.22
Psma1_1003	-1.81	-1.99	-7.37	-4.93	-4.59	-7.15	-11.13	0.94	-11.13	-11.13
Psma1_1003	-0.32	5.82	-0.4	0.36	-0.75	-2.96	-0.14	-1.94	-3.48	-4.76
Psma1_1003	-10.25	-10.25	-1.74	-3.73	-4.29	-11.08	-11.08	-11.08	-11.08	-11.08
Psma1_1003	-8.81	-8.81	-8.81	-4.43	-5.23	-11.15	-11.15	-11.15	-1.96	-11.15
Psma1_1003	-6.37	-6.37	-6.37	-3.65	-6.37	-10.44	-10.44	-10.44	-10.44	-10.44
Psma1_1003	-6.23	-5.79	-6.48	-4.16	-3.97	0.23	-10.7	-10.7	-10.7	-10.7
Psma1_1004	-1.85	-5.17	-3.4	-4.68	-4.3	-10.76	-10.76	-10.76	-10.76	-10.76

Table S2. Gene level dropout scores for each of the Kinome screens

**model**	**393P**				**344P**			
**condition**	**nude**		**in-vitro.2**		**nude**		**in-vitro.2**	
metric	logP	rank	logP	rank	logP	rank	logP	rank
Adrbk2	-5.86	4	-5.11	5	-5.29	1	-5.06	6
Cdk4	-4.98	7	-0.33	432	-5.04	2	-7.40	2
Csnk2a2	-1.71	101	-1.90	78	-4.36	3	-2.36	56
Dapk3	-3.43	21	-0.12	490	-4.29	4	-1.84	108
Wnk1	-2.80	40	-1.16	189	-4.23	5	-1.16	207
Rps6kc1	-1.57	117	-1.47	129	-4.07	6	-4.67	9
Btk	-0.54	368	-2.91	23	-3.90	7	-1.64	134
Rps6ka5	-1.64	106	-2.21	54	-3.90	8	-1.22	194
Kalrn	-2.86	37	-0.89	250	-3.87	9	-2.48	48
Aurka	-2.41	46	-2.72	33	-3.78	10	-5.49	4
Plk1	-5.44	6	-5.98	2	-3.68	11	-4.72	8
Hipk4	-0.18	474	-0.46	392	-3.64	12	-0.43	396
Bmpr1b	-0.70	322	-2.59	37	-3.57	13	-0.85	266
Akt1	-2.74	43	-1.44	134	-3.47	14	-1.49	156
Smg1	-2.09	70	-1.32	151	-3.46	15	-3.86	11
Stk16	-0.72	309	-0.43	403	-3.44	16	-3.28	19
Wnk2	-1.20	178	-1.04	216	-3.43	17	-3.16	21
Erbb3	-5.92	2	-4.34	6	-3.41	18	-2.64	40
Map2k5	-3.58	18	-2.47	43	-3.28	19	-5.97	3
Raf1	-5.50	5	-4.30	7	-3.28	20	-1.66	132
Flt4	-1.27	163	-2.10	62	-3.22	21	-3.69	14
Fes	-4.09	12	-2.86	27	-3.13	22	-1.64	136
Nek7	-4.06	13	-3.21	18	-3.07	23	-1.64	135
Akt3	-2.30	53	-1.67	110	-3.01	24	-0.79	286
Wnk3	-0.32	438	-0.61	335	-2.99	25	-0.97	242
Rps6ka6	-2.41	45	-3.46	14	-2.95	26	-2.17	70
Pomk	-0.48	383	-1.30	155	-2.87	27	-1.25	183
Mtor	-3.79	16	-5.49	4	-2.87	28	-2.52	45
Bub1b	-2.90	35	-3.56	12	-2.84	29	-3.62	15
Pik3cg	-0.16	482	-0.05	505	-2.74	30	-0.90	255
Stk35	-0.79	275	-1.38	143	-2.68	31	-0.77	291
Mark3	-2.31	52	-0.66	315	-2.68	32	-1.71	122
Csnk1e	-2.38	48	-2.11	59	-2.63	33	-1.61	139
Jak1	-1.65	105	-2.42	45	-2.59	34	-0.84	268
Nrbp1	-3.29	25	-1.79	92	-2.58	35	-1.67	130
Ttk	-1.32	153	-3.17	19	-2.54	36	-2.73	36
Tnik	-1.55	124	-1.85	82	-2.54	37	-0.49	378
Prkdc	-0.91	242	-0.86	257	-2.53	38	-2.02	86
Map3k7	-0.39	414	-0.04	509	-2.47	39	-0.10	488
Akt2	-1.57	121	-6.37	1	-2.46	40	-2.13	75
Cdkl4	-0.66	336	-0.59	342	-2.46	41	-0.38	417
Pik3ca	-3.82	15	-1.06	211	-2.45	42	-4.31	10

Pkn2	-1.87	85	-1.93	75	-2.43	43	-2.88	30
Bckdk	-0.97	230	-1.70	105	-2.35	44	-2.31	57
Adck4	-1.08	202	-1.78	94	-2.30	45	-2.30	58
Eif2ak3	-1.28	162	-1.29	159	-2.30	46	-3.17	20
Pi4ka	-0.77	289	-0.44	400	-2.29	47	-2.05	83
Atr	-5.98	1	-3.94	9	-2.27	48	-7.69	1
Stk38	-2.06	74	-1.01	223	-2.25	49	-0.92	249
Snrk	-2.09	69	-1.88	79	-2.24	50	-1.96	93
Pask	-1.58	115	-2.56	40	-2.23	51	-0.52	365
Sik3	-2.14	64	-1.42	140	-2.23	52	-1.55	145
Cask	-2.00	78	-1.99	69	-2.22	53	-1.54	148
Mapk12	-0.46	390	-0.41	414	-2.19	54	-1.73	120
Lats1	-0.70	321	-0.70	304	-2.19	55	-1.06	228
Gsg2	-1.58	114	-1.98	72	-2.17	56	-1.14	211
Mark1	-1.29	159	-2.03	67	-2.17	57	-2.44	51
Pak2	-1.19	181	-3.85	10	-2.16	58	-1.89	101
Mapk7	-0.59	360	-0.90	246	-2.16	59	-1.48	158
Prkg1	-2.86	38	-0.70	302	-2.14	60	-1.00	236
Ryk	-3.74	17	-1.05	214	-2.14	61	-3.84	12
Map3k6	-2.27	56	-0.35	427	-2.13	62	-2.44	52
Mastl	-1.19	183	-0.96	234	-2.13	63	-1.55	146
Stk3	-1.79	93	-1.25	170	-2.13	64	-1.98	90
Rps6kl1	-1.46	137	-0.32	434	-2.12	65	-2.10	80
Prkcg	-1.90	82	-1.30	156	-2.11	66	-0.54	360
Cdk10	-1.30	157	-0.58	349	-2.10	67	-1.80	111
Ntrk1	-0.04	513	-0.48	382	-2.10	68	-0.02	515
Ripk4	-0.63	346	-0.82	269	-2.10	69	-1.99	88
Mapk6	-0.85	256	-1.43	136	-2.10	70	-1.52	150
Pxk	-1.74	99	-2.07	64	-2.09	71	-1.75	118
Camk1d	-0.80	271	-0.48	384	-2.09	72	-0.86	263
Yes1	-3.37	23	-0.90	245	-2.05	73	-1.78	113
Prkab2	-1.08	198	-0.63	326	-2.04	74	-3.82	13
Nek3	-2.74	42	-0.96	233	-2.04	75	-2.52	44
Musk	-0.73	304	-0.43	402	-2.03	76	-3.10	23
Riok2	-0.80	272	-0.64	322	-2.03	77	-1.63	137
Vrk1	-1.21	170	-0.48	383	-2.02	78	-0.05	506
Pink1	-0.78	283	-3.26	16	-2.02	79	-3.07	24
Cdk6	-1.49	134	-0.87	255	-2.01	80	-2.18	67
LUC	-0.78	278	-1.24	173	-2.00	81	-1.61	140
Mapkapk5	-0.30	443	-1.70	106	-1.98	82	-1.92	95
Mapk9	-2.07	71	-1.98	73	-1.96	83	-1.92	97
Bmp2k	-1.46	138	-0.89	251	-1.96	84	-2.52	47
Epha5	-2.24	58	-1.83	86	-1.95	85	-1.77	114
Prkd3	-1.87	84	-0.22	466	-1.94	86	-0.63	332
Ern1	-1.19	180	-3.62	11	-1.93	87	-0.51	372
Sik1	-0.30	445	-0.42	404	-1.93	88	-0.65	326
Prkcd	-4.60	9	-2.87	26	-1.89	89	-2.54	43

Nek1	-1.75	96	-4.30	8	-1.88	90	-1.97	92
Trrap	-3.31	24	-3.26	17	-1.88	91	-2.03	84
Cnksr1	-0.25	456	-0.60	341	-1.88	92	-1.53	149
Mylk	-1.58	116	-0.27	449	-1.84	93	-1.25	184
Map2k6	-0.31	440	-0.66	316	-1.84	94	-1.38	167
Mast1	-3.50	19	-0.52	366	-1.84	95	-0.83	270
Map2k2	-1.19	182	-1.43	138	-1.83	96	-0.56	352
Map3k13	-0.14	491	-2.73	32	-1.82	97	-0.23	456
Epha1	-1.05	213	-0.83	266	-1.81	98	-0.64	327
Nek9	-1.36	148	-2.43	44	-1.81	99	-2.79	32
Epha4	-0.71	315	-1.03	219	-1.80	100	-0.40	408
Egfr	-1.86	87	-1.07	207	-1.79	101	-3.12	22
Dstyk	-1.06	205	-0.28	441	-1.79	102	-1.23	191
Ddr1	-0.85	257	-1.19	183	-1.78	103	-2.14	72
Fgfr4	-2.41	47	-2.78	29	-1.77	104	-2.66	38
Trib1	-1.57	120	-1.25	169	-1.76	105	-1.22	193
Chek1	-0.94	235	-1.10	199	-1.76	106	-1.45	161
Camk2a	-1.82	88	-1.27	161	-1.76	107	-0.87	259
Prkci	-0.24	460	-1.06	209	-1.75	108	-0.80	278
Map3k5	-0.55	367	-0.59	344	-1.75	109	-1.50	153
Met	-0.70	323	-0.37	424	-1.75	110	-0.13	479
Cdk7	-0.39	415	-0.05	504	-1.74	111	-0.45	389
Ptk2	-1.33	151	-1.29	158	-1.71	112	-3.02	27
Camkk1	-0.74	301	-0.96	235	-1.71	113	-0.59	343
Ulk1	-2.22	61	-0.63	330	-1.70	114	-0.42	403
Zap70	-1.07	203	-0.80	277	-1.68	115	-0.68	318
Srpk3	-1.06	204	-1.04	217	-1.68	116	-0.44	395
Map3k14	-1.21	175	-0.73	297	-1.67	117	-1.01	235
Crkl	-0.50	379	-0.82	268	-1.67	118	-1.26	181
Aatk	-1.63	108	-1.04	215	-1.66	119	-1.27	179
Pik3r3	-0.37	423	-0.02	514	-1.66	120	-0.57	350
Cdk8	-1.24	166	-2.08	63	-1.66	121	-2.13	74
Axl	-0.96	231	-0.67	313	-1.65	122	-0.62	335
Ddr2	-0.49	381	-1.05	212	-1.65	123	-1.11	219
Sgk2	-1.00	218	-0.24	462	-1.64	124	-0.63	331
Mok	-4.38	10	-1.84	83	-1.63	125	-1.83	110
Epha6	-0.77	291	-1.16	193	-1.62	126	-2.19	64
Stk32c	-2.80	41	-1.29	160	-1.62	127	-0.88	258
Rps6ka4	-0.93	238	-2.50	41	-1.61	128	-1.43	163
Sik2	-0.91	244	-1.07	208	-1.60	129	-1.15	209
Csk	-0.19	467	-0.66	317	-1.58	130	-0.86	265
Mknk1	-1.12	191	-0.59	346	-1.55	131	-0.60	339
Adck5	-3.48	20	-2.57	39	-1.54	132	-1.16	206
Rock2	-0.76	295	-0.91	241	-1.52	133	-0.45	388
Frk	-1.21	172	-2.18	55	-1.51	134	-0.89	256
Map4k1	-0.71	313	-1.02	222	-1.49	135	-1.12	217
Ret	-0.10	496	-0.35	428	-1.48	136	-0.37	418

Map3k2	-2.35	51	-1.56	120	-1.48	137	-0.25	449
Gm711	-1.10	193	-1.90	76	-1.47	138	-1.18	200
Camk4	-0.52	373	-0.21	472	-1.47	139	-1.27	177
Alpk3	-2.02	77	-0.51	372	-1.46	140	-0.18	469
Araf	-1.51	130	-0.53	363	-1.45	141	-0.99	239
Bmpr2	-0.77	290	-2.33	49	-1.45	142	-2.71	37
Npr2	-0.68	329	-0.48	381	-1.44	143	-0.91	253
Kdr	-0.28	449	-0.58	350	-1.44	144	-2.96	29
Mst1r	-1.28	161	-0.31	436	-1.44	145	-0.92	251
Txk	-0.50	378	-0.48	385	-1.43	146	-2.18	68
Stk17b	-0.70	324	-0.28	442	-1.42	147	-0.16	474
Cdk16	-0.96	232	-1.19	184	-1.42	148	-2.29	60
Pkn1	-0.91	241	-1.38	144	-1.41	149	-2.19	65
Csnk1d	-0.38	421	-0.54	361	-1.41	150	-0.55	354
Hipk3	-0.99	224	-1.56	119	-1.41	151	-0.79	280
Stk33	-1.68	104	-0.90	243	-1.41	152	-0.78	288
Ripk3	-0.10	500	-1.21	178	-1.40	153	-1.52	151
Bub1	-1.62	109	-2.12	57	-1.37	154	-1.19	198
Plk4	-0.83	264	-1.45	132	-1.37	155	-2.07	81
Mos	-0.60	357	-1.79	93	-1.37	156	-1.57	143
Pdpk1	-2.84	39	-2.70	34	-1.37	157	-2.37	55
Cdk11b	-1.41	145	-1.76	97	-1.36	158	-1.83	109
Mark4	-1.52	128	-0.80	274	-1.35	159	-3.00	28
Pak7	-0.92	239	-0.63	331	-1.35	160	-1.25	182
Mapk8	-1.50	133	-0.65	319	-1.35	161	-1.13	215
Aurkb	-4.96	8	-1.45	133	-1.35	162	-5.47	5
Dapk1	-1.76	95	-0.26	451	-1.34	163	-1.31	173
Styk1	-1.73	100	-0.37	421	-1.34	164	-2.28	61
Stk40	-1.57	118	-0.51	373	-1.33	165	-1.49	154
Map3k9	-0.40	412	-0.48	380	-1.33	166	-0.10	487
Prkch	-0.69	328	-0.77	285	-1.32	167	-0.31	436
Csnk1g1	-0.08	504	-1.16	191	-1.32	168	-0.75	302
Blk	-1.43	142	-1.26	167	-1.31	169	-1.35	170
Pim3	-1.29	160	-0.62	332	-1.31	170	-2.14	73
Fgr	-0.88	251	-0.72	299	-1.31	171	-0.76	297
Pdgfrb	-1.49	135	-0.83	265	-1.30	172	-0.31	438
Nek11	-1.87	83	-0.21	471	-1.30	173	-2.39	53
Trib2	-1.45	140	-1.15	194	-1.30	174	-0.53	363
Tbk1	-1.55	125	-2.33	50	-1.30	175	-2.02	85
Limk1	-0.45	393	-0.30	439	-1.30	176	-0.09	491
Sbk1	-1.44	141	-1.23	174	-1.29	177	-0.51	373
Ikbkb	-0.80	274	-0.51	375	-1.29	178	-1.75	117
Tex14	-4.20	11	-1.34	148	-1.28	179	-1.14	210
Jak2	-3.25	26	-2.26	52	-1.28	180	-1.94	94
Prkd2	-0.93	237	-0.99	228	-1.28	181	-2.45	50
Mapk14	-2.38	49	-1.57	117	-1.27	182	-0.77	289
Stk32b	-1.57	122	-1.32	153	-1.27	183	-1.37	168

Nim1k	-1.24	167	-1.36	145	-1.26	184	-1.17	202
Lrrk2	-1.51	129	-2.61	36	-1.25	185	-0.81	274
Map4k3	-3.08	29	-1.43	137	-1.24	186	-0.62	333
Mapk1	-0.83	263	-2.88	24	-1.21	187	-0.26	445
Pik3c3	-0.59	361	-0.64	321	-1.21	188	-0.54	361
Cdk9	-2.22	60	-1.11	196	-1.21	189	-0.47	385
Phkg2	-0.21	465	-0.13	488	-1.20	190	-2.10	77
Lmtk2	-0.47	388	-0.24	463	-1.20	191	-1.03	230
Mapk15	-3.08	30	-1.30	157	-1.19	192	-0.93	247
Eif2ak4	-0.97	228	-1.10	198	-1.18	193	-0.48	381
Cdkl5	-0.72	308	-0.25	459	-1.18	194	-1.22	196
Mapk4	-5.86	3	-0.64	324	-1.17	195	-0.48	382
Fastk	-0.83	262	-1.16	190	-1.17	196	-1.74	119
Riok1	-3.02	32	-2.88	25	-1.17	197	-3.39	18
Irak2	-1.61	110	-0.90	247	-1.15	198	-1.88	103
Fgfr1	-0.64	340	-0.39	417	-1.14	199	-0.68	320
Fert2	-1.09	195	-0.77	281	-1.13	200	-0.20	462
Ulk4	-0.66	337	-0.54	362	-1.13	201	-0.33	428
Erbb2	-3.02	31	-1.27	163	-1.12	202	-1.20	197
Mast4	-0.57	366	-0.26	452	-1.12	203	-0.77	290
Nek8	-0.13	493	-2.73	31	-1.12	204	-1.75	116
Trib3	-2.11	68	-1.27	162	-1.11	205	-0.79	281
Map3k11	-0.32	434	-1.04	218	-1.11	206	-1.18	201
Epha10	-1.18	184	-0.91	242	-1.11	207	-1.42	164
Scyl2	-1.08	197	-2.11	60	-1.11	208	-0.80	277
Insr	-2.13	65	-0.52	368	-1.11	209	-0.87	262
Ttbk1	-0.33	433	-0.81	271	-1.09	210	-0.76	298
Sgk3	-1.13	189	-0.59	347	-1.09	211	-0.33	431
Tec	-1.50	132	-0.86	258	-1.08	212	-2.19	66
Nrk	-0.78	281	-0.30	438	-1.08	213	-0.50	375
Stk19	-2.07	72	-0.41	413	-1.07	214	-2.25	63
Epha3	-0.41	409	-2.59	38	-1.06	215	-2.29	59
Riok3	-0.74	298	-1.03	220	-1.06	216	-1.98	89
Ror2	-0.99	222	-1.75	98	-1.05	217	-2.10	78
Map3k19	-0.86	254	-1.09	204	-1.05	218	-1.54	147
Pim1	-0.64	342	-1.42	139	-1.05	219	-1.51	152
Strada	-0.72	310	-0.63	329	-1.05	220	-0.05	505
Ttbk2	-1.48	136	-0.77	283	-1.05	221	-1.26	180
Pskh1	-1.80	90	-2.29	51	-1.03	222	-1.69	125
Tssk6	-1.74	98	-1.34	149	-1.03	223	-2.00	87
Ulk2	-1.34	149	-2.84	28	-1.03	224	-0.88	257
Hunk	-0.53	372	-1.16	188	-1.03	225	-1.69	126
Wee1	-3.17	27	-5.74	3	-1.02	226	-1.91	98
Mknk2	-0.70	319	-0.10	498	-1.02	227	-0.79	284
Aak1	-1.11	192	-1.32	152	-1.02	228	-1.13	213
Chek2	-0.88	252	-0.23	465	-1.02	229	-1.88	102
Pbk	-0.40	410	-0.37	425	-1.02	230	-0.76	295

Dclk3	-1.03	215	-0.42	406	-1.01	231	-1.00	238
Braf	-0.73	307	-1.96	74	-1.01	232	-0.18	466
Mylk4	-0.78	277	-0.58	352	-1.00	233	-0.74	304
Cdkl2	-0.26	453	-0.95	236	-1.00	234	-1.24	186
Ilk	-2.94	33	-1.36	146	-1.00	235	-1.68	128
Prkca	-0.58	362	-0.54	358	-1.00	236	-0.57	351
Mapk13	-0.75	296	-3.51	13	-0.98	237	-1.03	232
Myo3b	-0.45	391	-1.78	95	-0.98	238	-1.22	195
Ern2	-1.80	89	-1.81	89	-0.98	239	-3.05	25
Taf1	-2.35	50	-2.11	58	-0.97	240	-0.80	275
Nek10	-0.71	318	-1.05	213	-0.97	241	-0.55	355
Hipk2	-1.06	208	-1.74	100	-0.97	242	-1.11	218
Cdk14	-0.42	401	-0.07	502	-0.96	243	-0.74	303
Ripk1	-0.52	374	-0.24	461	-0.96	244	-1.23	189
Dclk2	-0.18	476	-0.75	293	-0.96	245	-0.08	500
Tnni3k	-1.77	94	-2.37	47	-0.96	246	-0.72	308
Matk	-0.90	247	-0.34	429	-0.96	247	-1.09	223
Trpm6	-1.21	174	-0.87	254	-0.95	248	-0.21	458
Tesk2	-1.64	107	-0.52	367	-0.95	249	-0.48	380
Stk24	-0.97	229	-0.78	279	-0.95	250	-0.06	502
Acvr1c	-0.96	233	-0.77	282	-0.95	251	-2.83	31
Tssk4	-0.41	406	-0.49	379	-0.94	252	-1.06	227
Acvr2a	-0.74	299	-0.51	371	-0.94	253	-0.61	338
Alk	-1.32	155	-1.74	101	-0.94	254	-1.23	190
Irak4	-2.51	44	-0.76	287	-0.93	255	-1.62	138
Fgfr2	-0.59	359	-0.79	278	-0.93	256	-2.18	69
Stk11	-1.71	102	-0.42	409	-0.93	257	-0.33	430
Amhr2	-0.38	420	-1.40	141	-0.93	258	-0.34	424
Cdk20	-1.08	201	-1.87	80	-0.92	259	-2.78	33
Itk	-0.34	427	-1.09	203	-0.91	260	-1.07	226
Map3k10	-2.89	36	-1.31	154	-0.90	261	-2.05	82
Dyrk2	-0.39	418	-0.67	310	-0.90	262	-0.39	409
Map2k3	-1.33	152	-0.31	437	-0.90	263	-0.54	356
Ptk2b	-0.71	312	-1.01	224	-0.90	264	-0.59	344
Mast3	-0.67	332	-0.47	387	-0.89	265	-0.58	347
Cdc42bpb	-0.35	426	-0.38	420	-0.89	266	-0.93	246
Cdk12	-0.65	338	-0.15	484	-0.89	267	-0.37	419
Nek2	-1.06	206	-0.93	239	-0.88	268	-0.72	307
Srms	-0.14	488	-0.11	493	-0.88	269	-0.35	422
Sbk2	-0.62	350	-0.81	272	-0.87	270	-1.91	99
Nuak1	-2.24	59	-0.47	388	-0.87	271	-0.33	426
Twf1	-1.60	111	-0.28	445	-0.86	272	-0.87	261
Ikbke	-0.69	325	-1.27	165	-0.85	273	-2.65	39
Scyl1	-1.54	126	-0.67	311	-0.85	274	-1.60	141
Wnk4	-1.79	91	-1.00	227	-0.83	275	-0.22	457
BC021891	-0.39	419	-0.44	398	-0.83	276	-1.41	165
Gucy2f	-1.26	164	-1.82	88	-0.83	277	-2.54	42

Tlk2	-1.56	123	-1.47	128	-0.83	278	-2.11	76
Grk6	-0.38	422	-0.12	492	-0.83	279	-0.08	497
Ankk1	-0.84	258	-1.09	205	-0.82	280	-0.69	316
Stk36	-2.06	73	-1.54	125	-0.80	281	-1.24	187
Pdk1	-1.20	179	-2.10	61	-0.80	282	-0.91	252
Hipk1	-1.94	80	-0.64	323	-0.79	283	-2.63	41
Ulk3	-2.28	55	-1.19	180	-0.78	284	-1.79	112
Eif2ak1	-0.28	452	-0.14	486	-0.78	285	-0.09	494
Mapk11	-0.31	442	-0.49	378	-0.78	286	-0.64	330
D8Ertd82e	-0.37	424	-0.18	479	-0.77	287	-0.17	472
Tesk1	-0.74	303	-0.23	464	-0.77	288	-0.15	475
Lats2	-0.30	444	-0.89	249	-0.77	289	-1.48	157
Obscn	-0.68	331	-0.71	301	-0.77	290	-0.21	460
Pkn3	-0.41	404	-0.40	416	-0.77	291	-0.28	443
Rps6kb2	-2.18	63	-2.92	22	-0.77	292	-2.10	79
Lmtk3	-1.57	119	-1.63	115	-0.76	293	-0.46	386
Mink1	-0.86	253	-0.80	276	-0.76	294	-0.75	301
Plk3	-2.25	57	-0.45	397	-0.76	295	-2.46	49
Tek	-1.01	217	-0.45	395	-0.76	296	-1.76	115
Pkdcc	-0.85	255	-1.18	185	-0.74	297	-2.77	34
Trio	-0.59	358	-0.21	470	-0.74	298	-2.26	62
Trp53rk	-0.78	282	-0.77	284	-0.74	299	-2.16	71
Pdk4	-3.42	22	-1.54	124	-0.74	300	-0.51	369
Kit	-0.12	494	-0.75	291	-0.73	301	-0.71	310
Fgfr3	-0.58	365	-0.81	270	-0.73	302	-0.80	276
Limk2	-0.91	245	-0.13	489	-0.73	303	-0.96	244
Adck2	-0.83	265	-0.98	229	-0.73	304	-0.50	376
Epha7	-1.45	139	-0.75	292	-0.72	305	-0.10	489
Grk5	-0.62	354	-0.25	460	-0.72	306	-0.44	394
Melk	-0.33	431	-0.83	267	-0.72	307	-0.79	279
Prkaa1	-0.32	436	-0.97	231	-0.71	308	-1.89	100
Ksr2	-0.84	260	-0.55	357	-0.70	309	-0.62	336
Vrk2	-1.25	165	-0.63	328	-0.70	310	-4.95	7
Pi4k2a	-0.39	417	-1.71	102	-0.69	311	-3.48	16
Rps6kb1	-0.58	363	-0.92	240	-0.69	312	-1.55	144
Camk2g	-1.43	143	-0.10	496	-0.69	313	-0.26	447
Map3k15	-3.17	28	-2.35	48	-0.68	314	-1.67	131
Pik3c2a	-0.98	227	-1.75	99	-0.68	315	-0.73	305
Jak3	-1.03	214	-1.06	210	-0.68	316	-0.56	353
Cit	-1.00	221	-0.10	500	-0.68	317	-0.24	455
Mark2	-0.88	250	-1.47	131	-0.67	318	-1.40	166
Cdk17	-0.77	288	-0.46	390	-0.67	319	-0.37	420
Rnasel	-0.23	461	-0.08	501	-0.67	320	-0.08	498
Trpm7	-1.92	81	-0.90	244	-0.66	321	-1.72	121
Myo3a	-0.44	394	-1.98	70	-0.66	322	-0.45	390
Lck	-0.90	246	-1.08	206	-0.66	323	-0.24	454
Erbb4	-0.32	437	-0.19	477	-0.65	324	-0.70	314

BC030499	-0.34	428	-1.57	118	-0.65	325	-0.42	400
Tie1	-1.42	144	-1.82	87	-0.65	326	-0.51	370
Pak6	-0.69	327	-0.26	454	-0.65	327	-0.12	482
Eif2ak2	-0.74	300	-0.71	300	-0.65	328	-1.05	229
Cdk13	-0.76	292	-0.76	286	-0.65	329	-0.02	512
Taok2	-0.90	248	-1.65	113	-0.65	330	-1.27	178
Camkk2	-0.15	485	-0.25	455	-0.65	331	-0.09	496
Ripk2	-0.70	320	-1.47	130	-0.64	332	-2.52	46
Stk31	-1.74	97	-1.35	147	-0.64	333	-1.30	175
Nrbp2	-1.21	171	-3.05	20	-0.64	334	-0.73	306
Flt1	-1.05	212	-1.67	109	-0.64	335	-0.52	366
Cdkl1	-2.05	75	-2.67	35	-0.63	336	-0.67	322
Vrk3	-1.20	176	-0.75	294	-0.63	337	-0.79	285
Pak1	-0.02	515	-0.47	389	-0.63	338	-0.33	427
Nuak2	-0.18	475	-0.37	423	-0.63	339	-1.68	129
Adck1	-0.47	386	-0.10	499	-0.62	340	-0.54	357
Ntrk3	-1.24	168	-1.51	127	-0.62	341	-0.38	415
Mertk	-0.78	279	-1.84	84	-0.62	342	-1.17	203
Ptk6	-0.75	297	-1.52	126	-0.62	343	-0.62	334
Bmpr1a	-0.33	432	-2.00	68	-0.61	344	-0.35	421
Pik3r2	-1.05	209	-1.90	77	-0.61	345	-1.00	237
Grk1	-1.21	173	-0.89	248	-0.60	346	-0.54	359
Prpf4b	-3.86	14	-2.78	30	-0.59	347	-3.03	26
Abl2	-0.01	516	-0.51	374	-0.59	348	-0.41	405
Ptk7	-2.92	34	-0.51	369	-0.59	349	-2.39	54
Dmpk	-0.63	347	-1.70	104	-0.59	350	-0.28	441
Dapk2	-0.29	447	-0.84	262	-0.58	351	-1.08	225
Aurkc	-0.26	454	-1.86	81	-0.58	352	-0.71	311
Lrrk1	-0.63	348	-1.27	166	-0.58	353	-1.13	216
Phkg1	-0.74	302	-0.42	410	-0.58	354	-1.17	205
Csnk2a1	-1.86	86	-1.55	122	-0.58	355	-1.69	127
Acvrl1	-0.71	316	-0.41	411	-0.57	356	-0.13	481
Cdk1	-2.12	66	-1.09	201	-0.57	357	-1.47	160
Eef2k	-0.62	351	-0.21	468	-0.57	358	-1.33	171
Irak3	-0.19	468	-1.02	221	-0.57	359	-0.26	448
Pdik1l	-1.12	190	-0.52	365	-0.56	360	-0.18	468
Dyrk4	-0.83	267	-1.84	85	-0.56	361	-1.13	214
Cdk18	-0.78	284	-0.28	443	-0.56	362	-1.03	231
Bmx	-0.61	355	-0.85	260	-0.56	363	-0.77	294
Tgfbr2	-0.41	407	-0.87	256	-0.56	364	-0.70	315
Bcr	-0.43	396	-0.67	314	-0.55	365	-0.75	300
Pik3c2g	-1.58	113	-0.60	339	-0.55	366	-0.54	358
Camk1g	-1.59	112	-1.27	164	-0.55	367	-1.01	233
Mylk2	-1.30	156	-0.61	337	-0.55	368	-1.98	91
Srpk2	-0.25	455	-0.12	491	-0.54	369	-0.41	406
Dyrk3	-0.28	451	-0.88	252	-0.54	370	-0.20	461
Srpk1	-0.05	510	-0.83	263	-0.54	371	-0.17	471

Tbck	-0.53	370	-0.18	481	-0.53	372	-0.08	499
Ephb3	-0.68	330	-0.68	308	-0.53	373	-0.38	416
Prkab1	-1.37	146	-0.62	334	-0.53	374	-0.67	321
Stk38l	-1.34	150	-0.81	273	-0.53	375	-1.65	133
Pik3cb	-0.65	339	-0.10	497	-0.52	376	-0.03	511
Brsk2	-1.08	199	-0.44	401	-0.52	377	-0.39	413
Rps6ka1	-0.20	466	-2.07	65	-0.52	378	-0.19	464
Map2k7	-0.42	403	-0.44	399	-0.52	379	-0.27	444
Stk10	-1.20	177	-0.69	305	-0.51	380	-0.39	412
Pik3cd	-0.10	497	-0.27	448	-0.51	381	-0.18	470
Map4k2	-0.99	225	-1.67	111	-0.51	382	-1.19	199
Ntrk2	-0.32	435	-1.22	176	-0.51	383	-1.10	221
Zak	-1.09	196	-0.21	469	-0.50	384	-1.87	105
Igf1r	-0.72	311	-0.54	359	-0.49	385	-0.42	399
Ephb2	-0.42	402	-0.27	446	-0.49	386	-0.54	362
Fyn	-0.71	317	-0.18	478	-0.49	387	-1.15	208
Pdgfra	-1.23	169	-0.18	480	-0.48	388	-0.76	296
Csf1r	-0.77	286	-1.24	172	-0.47	389	-0.43	397
Cdk19	-0.53	369	-0.04	511	-0.47	390	-0.29	439
Mak	-0.51	377	-0.59	343	-0.47	391	-0.04	509
Scyl3	-0.18	471	-0.45	396	-0.47	392	-0.64	328
Csnk1g3	-1.36	147	-2.15	56	-0.46	393	-0.44	393
Nlk	-0.48	384	-1.00	225	-0.45	394	-0.41	407
Ick	-0.31	439	-0.33	431	-0.45	395	-0.71	309
Map3k8	-0.15	483	-0.39	418	-0.45	396	-0.76	299
Prkacb	-1.00	220	-0.58	351	-0.45	397	-0.87	260
Map3k1	-0.62	353	-0.01	515	-0.45	398	-0.09	493
Alpk1	-0.29	448	-0.51	376	-0.45	399	-0.19	465
Camk2b	-0.19	470	-0.69	307	-0.45	400	-0.70	313
Ephb1	-0.71	314	-0.76	290	-0.45	401	-0.83	271
Cdc7	-0.18	473	-0.59	348	-0.44	402	-0.86	264
Acvr2b	-1.09	194	-0.97	232	-0.43	403	-0.49	379
Insrr	-2.20	62	-0.72	298	-0.43	404	-0.92	250
Map3k4	-0.82	270	-0.56	353	-0.43	405	-0.19	463
Hck	-0.82	269	-1.26	168	-0.43	406	-0.15	476
Cdkl3	-0.91	243	-0.22	467	-0.42	407	-0.11	483
Prkcb	-0.73	305	-0.06	503	-0.41	408	-0.70	312
Dclk1	-0.29	446	-0.46	393	-0.41	409	-0.39	411
Lyn	-1.32	154	-1.12	195	-0.41	410	-0.50	377
Adrbk1	-0.04	512	-0.20	476	-0.40	411	0.00	516
Irak1	-0.92	240	-0.69	306	-0.40	412	-0.58	346
2610018G03Rik	-1.95	79	-1.80	91	-0.40	413	-0.79	282
Ttn	-1.71	103	-1.66	112	-0.40	414	-0.66	324
Prkaa2	-0.40	411	-0.49	377	-0.39	415	-0.42	404
Tyro3	-0.24	458	-0.46	391	-0.39	416	-0.42	401
Ephb6	-0.14	489	-0.11	494	-0.39	417	-1.30	174
Pnck	-0.04	511	-0.25	458	-0.39	418	-1.69	124

Pdk2	-0.07	506	-0.39	419	-0.38	419	-0.43	398
Pak3	-1.52	127	-1.61	116	-0.37	420	-1.71	123
Tlk1	-0.64	344	0.00	517	-0.37	421	-0.35	423
Taok3	-0.43	398	-0.34	430	-0.37	422	-0.52	368
Epha8	-0.78	280	-0.88	253	-0.37	423	-0.57	349
Cdk2	-0.07	507	-0.14	485	-0.37	424	-1.85	106
Brsk1	-0.17	477	-0.26	453	-0.37	425	-0.57	348
Tssk1	-1.08	200	-0.80	275	-0.37	426	-0.09	495
Tyk2	-0.33	430	-1.19	179	-0.36	427	-1.36	169
Pi4kb	-1.02	216	-0.03	513	-0.36	428	-0.77	292
Camk1	-1.50	131	-1.11	197	-0.36	429	-0.48	384
Sgk1	-0.44	395	-0.51	370	-0.36	430	-1.49	155
Mapkapk3	-0.41	408	-0.37	422	-0.36	431	-0.33	429
Map4k4	-0.13	492	-0.55	355	-0.36	432	-0.68	319
Adck3	-0.14	490	-0.53	364	-0.36	433	-0.39	414
Cdk15	-0.21	464	-0.83	264	-0.35	434	-1.87	104
Rps6ka3	-0.52	375	-0.66	318	-0.35	435	-0.32	432
Src	-0.89	249	-1.18	186	-0.35	436	-0.60	341
Pik3c2b	-0.18	472	-0.73	296	-0.34	437	-0.25	451
Tnk1	-1.17	185	-0.56	354	-0.34	438	-0.66	325
Gsk3a	-0.94	236	-0.13	487	-0.34	439	-0.60	342
Epha2	-0.69	326	-0.17	482	-0.33	440	-0.28	442
Pim2	-0.83	266	-1.77	96	-0.33	441	-0.32	434
Csnk1g2	-0.84	259	-1.18	187	-0.32	442	-0.50	374
Prkd1	-1.79	92	-1.22	177	-0.32	443	-1.17	204
Ros1	-0.14	487	-0.61	338	-0.32	444	-1.11	220
Acvr1b	-0.67	334	-1.70	103	-0.32	445	-1.09	224
Gsk3b	-1.06	207	-0.41	412	-0.31	446	-0.53	364
Taok1	-0.08	505	-0.30	440	-0.31	447	-0.09	490
Prkaca	-1.30	158	-0.20	474	-0.30	448	-1.27	176
Stk32a	-0.73	306	-1.19	182	-0.30	449	-2.77	35
Gak	-0.83	261	-1.22	175	-0.29	450	-0.32	435
Pdk3	-0.79	276	-1.68	107	-0.29	451	-1.25	185
Map3k12	-0.36	425	-0.05	508	-0.29	452	-0.24	453
Pik3r5	-1.17	186	-0.76	288	-0.29	453	-0.77	293
Map2k1	-0.39	416	-1.40	142	-0.28	454	-0.52	367
Mylk3	-0.22	463	-1.68	108	-0.27	455	-0.83	272
Chuk	-0.64	343	-0.54	360	-0.27	456	-0.29	440
Peak1	-0.31	441	-0.33	433	-0.27	457	-0.02	514
Mapk10	-0.46	389	-1.81	90	-0.27	458	-1.23	192
Plk2	-0.47	387	-0.63	325	-0.27	459	-0.64	329
Mast2	-0.28	450	-0.42	407	-0.26	460	-0.15	477
Syk	-0.15	486	-1.54	123	-0.26	461	-1.01	234
Stk25	-0.24	459	-0.62	333	-0.25	462	-0.79	283
Ephb4	-0.76	294	-0.64	320	-0.24	463	-0.78	287
Clk2	-0.63	349	-0.21	473	-0.24	464	-0.48	383
Prkce	-2.12	67	-1.98	71	-0.24	465	-3.45	17

Prkg2	-0.76	293	-0.74	295	-0.24	466	-0.85	267
Clk4	-0.67	335	-0.03	512	-0.23	467	-0.06	501
Nek6	-0.99	223	-0.35	426	-0.23	468	-0.66	323
Clk1	-0.67	333	-0.59	345	-0.23	469	-1.32	172
Tssk2	-0.98	226	-1.09	202	-0.23	470	-0.92	248
Acvr1	-0.58	364	-0.63	327	-0.22	471	-0.99	240
Hspb8	-1.00	219	-2.23	53	-0.22	472	-1.84	107
Ror1	-0.41	405	-0.47	386	-0.21	473	-0.09	492
Abl1	-0.77	287	-1.09	200	-0.21	474	-1.44	162
Stk39	-0.47	385	-1.16	192	-0.21	475	-0.61	337
Mapk3	-0.17	479	0.00	516	-0.21	476	-0.32	433
Cdk5	0.00	517	-0.40	415	-0.20	477	-0.34	425
Camkv	-0.40	413	-0.76	289	-0.20	478	-0.05	504
Csnk1a1	-2.30	54	-1.43	135	-0.20	479	-0.11	485
Prkcq	-0.10	499	-1.24	171	-0.20	480	-0.05	503
Dyrk1a	-0.06	509	-3.43	15	-0.20	481	0.00	517
Pik3r4	-0.64	345	-2.38	46	-0.20	482	-0.69	317
Gucy2c	-0.95	234	-0.98	230	-0.20	483	-0.14	478
Clk3	-0.09	502	-0.93	237	-0.20	484	-0.90	254
Tgfbr1	-0.51	376	-0.93	238	-0.19	485	-0.11	484
Nek4	-0.80	273	-0.32	435	-0.19	486	-1.58	142
Camk2d	-0.42	400	-0.42	408	-0.19	487	-0.60	340
Pak4	-0.77	285	-0.67	309	-0.18	488	-1.92	96
Magi1	-1.05	210	-2.47	42	-0.18	489	-0.13	480
Twf2	-0.49	380	-1.00	226	-0.18	490	-0.21	459
Flt3	-0.17	478	-0.25	457	-0.17	491	-0.02	513
Map3k3	-1.05	211	-1.56	121	-0.17	492	-0.51	371
Map2k4	-0.09	501	-0.05	506	-0.17	493	-0.45	391
Map4k5	-0.08	503	-0.85	259	-0.16	494	-0.39	410
Alpk2	-0.62	352	-0.25	456	-0.15	495	-0.24	452
Tssk3	-0.22	462	-0.60	340	-0.15	496	-0.25	450
Tnk2	-0.64	341	-2.03	66	-0.14	497	-0.59	345
Mapkapk2	-0.53	371	-0.27	447	-0.14	498	-0.97	243
Prkx	-1.14	188	-0.85	261	-0.14	499	-0.99	241
Stk4	-2.04	76	-1.63	114	-0.14	500	-1.09	222
Slk	-0.15	484	-1.19	181	-0.13	501	-0.84	269
Nek5	-0.02	514	-0.05	507	-0.13	502	-0.03	510
Pkmyt1	-0.25	457	-0.61	336	-0.13	503	-0.11	486
Atm	-0.42	399	-0.42	405	-0.12	504	-1.13	212
Rps6ka2	-0.16	481	-0.55	356	-0.12	505	-1.48	159
Ltk	-0.10	498	-0.70	303	-0.11	506	-0.94	245
Oxsr1	-0.43	397	-0.28	444	-0.11	507	-0.45	392
Mlkl	-0.83	268	-1.33	150	-0.10	508	-0.04	508
Rock1	-0.49	382	-0.11	495	-0.10	509	-0.42	402
Npr1	-0.33	429	-0.04	510	-0.08	510	-1.23	188
Grk4	-0.45	392	-0.78	280	-0.07	511	-0.26	446
Dyrk1b	-0.07	508	-0.20	475	-0.06	512	-0.04	507

Prkcz	-1.14	187	-0.67	312	-0.05	513	-0.82	273
Gucy2e	-0.19	469	-2.96	21	-0.05	514	-0.46	387
Uhmk1	-0.12	495	-0.27	450	-0.05	515	-0.18	467
Pik3r1	-0.60	356	-0.46	394	-0.03	516	-0.16	473
Cdc42bpa	-0.16	480	-0.16	483	-0.01	517	-0.31	437

Table S3. Quality control metrics for the FDAome shRNA screens

	393P			344P		
	in-vivo (sv129)			in-vivo (sv129)		
reagent	1	2	3	1	2	3
LUC_CONT	-0.44	-0.99	0.24	-0.15	1.02	0.93
LUC_CONT	0.58	1.24	0.95	2.21	0.42	0.68
LUC_CONT	3.67	1.45	1.3	0.98	1.36	1.57
LUC_CONT	0.63	0.24	1.16	1.86	0.96	0.91
LUC_CONT	0.56	1.03	-0.32	0.53	1.6	0.02
LUC_CONT	1	0.52	-0.3	3.2	0.36	1.44
LUC_CONT	1.7	-0.11	1.08	1.34	2.09	1.42
LUC_CONT	0.42	0.38	0.29	1.5	1.36	1.5
LUC_CONT	0.97	1.68	0.11	1.37	-0.45	1.82
LUC_CONT	0.63	0.86	1.44	0.98	0.56	0.68
LUC_CONT	0.03	0.03	-0.39	0.43	0.16	0.3
LUC_CONT	0.5	0.6	0.46	0.35	-0.07	-0.07
LUC_CONT	-0.35	0.5	0.39	0.28	0.84	0.19
LUC_CONT	0.08	0.1	0.61	1.55	0.47	0.87
LUC_CONT	1.03	0.19	0.46	1.94	1.41	1.36
LUC_CONT	-0.46	-1.33	0.1	-0.56	-0.42	0.53
LUC_CONT	-0.9	-0.08	-0.29	-2.49	-0.73	-0.57
LUC_CONT	-1.48	-1.77	-0.53	-3.62	-1.35	-1.95
LUC_CONT	2.21	-0.97	2.33	-1.41	-2.23	-1.17
LUC_CONT	1.51	-1.22	-0.81	0.83	0.56	-0.12
Rpl30_CON	-3.66	-2.43	-6.01	-2.15	-5.47	-3.24
Rpl30_CON	-2.5	-2.2	-2.32	-2.16	-1.2	-2.24
Rpl30_CON	-2.81	-1.29	-1.84	-3.33	-2.66	-2.27
Rpl30_CON	-3.06	-2.16	-0.55	-1.52	-4.67	-4.42
Rpl30_CON	-4.35	-1.56	-7.23	-0.75	-3.11	-2.37
Rpl30_CON	-3.06	0.62	-2.12	-2.08	-1.62	3.62
Rpl30_CON	0.65	-2.41	-2.32	-3.85	-4.82	-1.05
Rpl30_CON	-1.19	-2.45	-1.86	-3.07	-2.75	4.05
Rpl30_CON	-1.83	-3.17	-5.23	-1.66	-5.34	-5.74
Rpl30_CON	-2.83	-2.35	-1.37	-2.67	-1.37	-1.43
Rpl30_CON	-1.45	-2.73	-0.46	-1.24	-0.96	-2.42
Rpl30_CON	-2.74	-0.93	-1.62	-1.71	-1.22	0.67
Rpl30_CON	-1.94	-2.15	-6.81	-4.87	-3.79	-5.3
Rpl30_CON	0.23	-1.86	-0.83	-2.45	0.16	-4.01
Rpl30_CON	-3.53	-3.75	2.1	2.08	3.35	3.3
Rpl30_CON	-2.34	-1.22	-1.02	-0.08	1.23	-0.08
Rpl30_CON	-1.35	-0.7	-2.25	-0.83	-2.53	0.91
Rpl30_CON	-2.4	-1.98	-1.94	-4.46	-0.8	-4.82
Rpl30_CON	-0.31	-0.07	-1.1	-1.2	-1.66	-0.3
Rpl30_CON	-1.82	-2.41	-0.65	-1.66	-0.59	0.17
Psma1_CO	-0.4	0.14	-0.21	0.07	-0.15	0.87
Psma1_CO	-0.48	0.12	-0.08	0.14	-0.8	0.57
Psma1_CO	-0.17	0.69	1.12	0.68	-0.82	1.08

Psma1_CO	-0.4	-0.13	0.39	0.32	-0.91	0.45
Psma1_CO	-0.64	1.2	-0.55	0.14	-0.77	0.69
Psma1_CO	-1.87	-1.15	-2.89	-3.15	-4.68	-2.21
Psma1_CO	-0.79	-2.69	-2.16	-3.74	-3.26	-2.09
Psma1_CO	-2.09	-1.83	-2.47	-1.06	-4.2	-0.41
Psma1_CO	-1.21	-1.04	-1.45	-3.77	-3.23	0.28
Psma1_CO	-0.13	-3.03	-1.85	-3.59	-0.89	-0.42
Psma1_CO	-5.79	-1.62	-2.03	-1.25	-1.41	-1.49
Psma1_CO	-2.39	-0.92	-3.94	2.18	1.05	-1.39
Psma1_CO	-3.55	-1.32	-1.94	-1.8	-2.79	-1.31
Psma1_CO	0.38	-2.77	-2.26	-0.4	0.05	-1.54
Psma1_CO	3.07	-2.07	-0.04	-0.66	-0.29	-1.57
Psma1_CO	-2.24	-2.29	-2.23	0.19	-1.96	-2.18
Psma1_CO	-1.99	-3.39	-5.29	-2.93	-0.8	-0.4
Psma1_CO	-0.88	-3.44	-3.52	-1.28	-2.67	-0.79
Psma1_CO	-3.08	-1.86	-1.86	1.67	-1.34	0.48
Psma1_CO	-1.98	-2.77	-4.74	-0.5	1.74	-0.22

Table S4. Gene level dropout scores for each of the FDAome screens

gene	393P sv129 logP	393P sv129 rank	344P sv129 logP	344P sv129 rank
Pik3ca	-5.5	4	-8.4	1
Cdk4	-3.2	16	-7.8	2
Myc	-4.1	11	-7.7	3
Bcl2	-2.9	18	-6.3	4
Prkcd	-4.4	8	-5.8	5
Cdk7	-0.7	110	-5.4	6
Ctnnb1	-0.8	102	-5.1	7
Plk1	-2.9	19	-4.7	8
Mtor	-2.9	17	-4.3	9
Cdk6	-2.0	32	-4.2	10
Psmd1	-4.4	9	-4.1	11
Top2b	-0.2	164	-4.0	12
Psmb1	-10.1	1	-3.9	13
Rarb	-0.8	96	-3.9	14
Erbb2	-5.4	5	-3.7	15
Mcl1	-1.4	56	-3.3	16
Esr1	-4.5	7	-3.2	17
Birc5	-0.9	88	-3.1	18
Insr	-1.8	39	-2.9	19
Rac1	-6.5	3	-2.8	20
Fgfr4	-1.3	61	-2.8	21
Tec	-1.1	69	-2.7	22
Top1	-1.4	54	-2.5	23
Epha2	-1.7	42	-2.5	24
Notch1	-1.7	41	-2.5	25
Itk	-0.8	100	-2.5	26
Rarg	-1.1	73	-2.5	27
Ptpn6	-1.1	68	-2.4	28
Raf1	-8.7	2	-2.4	29
Stat3	-4.8	6	-2.4	30
Axl	-0.7	114	-2.2	31
Gsk3a	-2.7	20	-2.2	32
Akt2	-0.2	162	-2.2	33
Pim1	-1.2	64	-2.2	34
Wee1	-2.6	23	-2.1	35
Top2a	-4.2	10	-2.1	36
Mapk3	-0.3	160	-2.1	37
Parp3	-1.2	65	-2.1	38
Prkcg	-1.0	81	-2.0	39
Chek2	-0.1	175	-2.0	40
Trim24	-1.6	48	-2.0	41
Whsc1	-1.2	63	-2.0	42
Cd274	-0.4	152	-1.9	43

Tnfsf13b	-0.2	167	-1.9	44
Prkci	-0.2	166	-1.9	45
Atr	-1.8	36	-1.9	46
Jak1	-0.7	112	-1.9	47
Aurkb	-2.2	28	-1.9	48
Cdk9	-1.3	60	-1.8	49
Mapk1	-3.6	13	-1.8	50
Aurka	-1.7	43	-1.8	51
Fgr	-0.1	170	-1.8	52
Pak4	-2.0	30	-1.8	53
Abl2	-0.1	172	-1.8	54
Mapk7	-1.9	35	-1.7	55
Mapk12	-0.7	104	-1.7	56
Flt4	-1.1	74	-1.7	57
Pik3cg	-1.2	66	-1.6	58
Rps6kb1	-0.7	107	-1.6	59
Hdac1	-0.6	123	-1.6	60
Tyms	-0.1	180	-1.6	61
Jak2	-2.6	22	-1.5	62
Met	-2.3	25	-1.5	63
Tyro3	-1.1	76	-1.5	64
Fgfr1	-0.9	91	-1.5	65
Ptgs2	-1.1	70	-1.5	66
Rrm1	-2.0	31	-1.5	67
Xpo1	-1.6	50	-1.5	68
Ntrk1	-1.4	55	-1.5	69
Egfr	-1.5	51	-1.4	70
Chek1	-0.2	163	-1.4	71
Cdk2	-1.8	37	-1.4	72
Gls	-0.4	144	-1.4	73
Parp1	-0.7	113	-1.4	74
Esr2	-1.1	77	-1.4	75
Mapk9	-0.5	135	-1.4	76
Dot1l	-1.1	72	-1.3	77
Il1b	-0.7	116	-1.3	78
Ptk2	-1.7	40	-1.3	79
Hdac3	-0.5	141	-1.3	80
Eif4e	-3.8	12	-1.3	81
Map3k14	-0.6	130	-1.3	82
Ehmt2	-1.9	34	-1.2	83
P4hb	-0.8	103	-1.2	84
Mapk11	-1.7	45	-1.2	85
Kdm1a	-1.0	86	-1.2	86
Brd4	-1.0	80	-1.2	87
Ppm1d	-0.7	111	-1.2	88
Mapk8	-1.3	59	-1.1	89
Abl1	0.0	187	-1.1	90

Mapk14	-0.4	153	-1.1	91
Nr2c2	-0.5	139	-1.0	92
Pigf	-0.4	146	-1.0	93
Lyn	-0.6	128	-1.0	94
Rxrb	-1.8	38	-1.0	95
Flt1	-1.2	67	-1.0	96
Ikbke	-2.2	27	-0.9	97
Cdk1	-1.0	83	-0.9	98
Prkdc	-3.4	14	-0.9	99
Alk	-0.7	106	-0.9	100
Irak4	-0.4	149	-0.9	101
Hsp90aa1	-1.4	53	-0.9	102
Smo	-0.8	97	-0.8	103
Casp3	-0.5	143	-0.8	104
Prkca	-0.9	93	-0.8	105
Syk	0.0	188	-0.8	106
Pdgfra	-0.5	134	-0.8	107
Src	-0.8	99	-0.8	108
Vegfa	-1.0	85	-0.8	109
Pgd	-1.3	57	-0.8	110
Bcr	-0.6	126	-0.8	111
Ezh2	-0.9	89	-0.8	112
Map4	-2.5	24	-0.8	113
Tubb4a	-1.0	87	-0.7	114
Parp2	-0.7	105	-0.7	115
Pdgfrb	-0.6	118	-0.7	116
Map2k1	-1.1	71	-0.7	117
Prkce	-0.8	95	-0.7	118
Txn1	-0.5	131	-0.7	119
Btk	-1.7	46	-0.7	120
Il6	-0.4	155	-0.7	121
Ccr5	-1.0	79	-0.7	122
Frk	-0.9	90	-0.7	123
Idh2	-0.1	186	-0.7	124
Mapk13	-0.4	148	-0.7	125
Tnfrsf8	-0.3	159	-0.7	126
Prkch	-0.7	108	-0.7	127
Ptch1	-0.6	127	-0.7	128
Rock2	-1.5	52	-0.6	129
Nfkb1	-2.7	21	-0.6	130
Fyn	-2.1	29	-0.6	131
Pdk1	-0.2	165	-0.5	132
Gsk3b	-1.7	44	-0.5	133
Fgfr2	-1.0	78	-0.5	134
Map3k8	-0.3	161	-0.5	135
Crebbp	-0.1	179	-0.5	136
Kit	-0.1	176	-0.5	137

Ar	-0.4	147	-0.4	138
Hdac6	-0.6	117	-0.4	139
Mapt	-0.7	109	-0.4	140
Xiap	-0.6	121	-0.4	141
Atm	-1.1	75	-0.4	142
Prkaa1	-0.4	154	-0.4	143
Tnf	-0.6	119	-0.4	144
Ret	-0.1	177	-0.4	145
Idh1	-0.5	132	-0.4	146
Hdac2	-0.6	129	-0.4	147
Dhfr	-3.4	15	-0.4	148
Drd2	-1.6	49	-0.4	149
Il6ra	-0.9	94	-0.4	150
Jak3	-0.1	173	-0.3	151
Blk	-0.8	101	-0.3	152
Pik3cd	-1.3	58	-0.3	153
Map2	-0.2	168	-0.3	154
Mknk1	-0.3	156	-0.3	155
Nampt	-0.6	125	-0.3	156
Lap3	-1.0	82	-0.3	157
Lck	0.0	191	-0.3	158
Rxra	-0.2	169	-0.3	159
Fgfr3	-0.6	120	-0.3	160
Akt1	-0.8	98	-0.2	161
Aurkc	-0.5	133	-0.2	162
Prkcq	-0.9	92	-0.2	163
Mdm2	-0.5	138	-0.2	164
Tek	-0.1	174	-0.2	165
Prkcb	-0.7	115	-0.2	166
Braf	-0.6	124	-0.2	167
Hdac8	-0.5	136	-0.2	168
Ldha	-1.6	47	-0.2	169
Prkcsh	-1.9	33	-0.2	170
Rara	-0.1	171	-0.2	171
Pim3	-0.4	150	-0.2	172
Prkcz	-0.1	182	-0.2	173
Akt3	-0.5	137	-0.1	174
Tnfsf11	-1.2	62	-0.1	175
Ms4a1	-0.4	151	-0.1	176
Trpv1	-0.5	140	-0.1	177
Ptpn11	-2.3	26	-0.1	178
Nudt1	-0.1	183	-0.1	179
Pik3cb	-1.0	84	-0.1	180
Flt3	-0.4	145	-0.1	181
Cd52	-0.1	185	-0.1	182
Bmx	0.0	192	-0.1	183
Kdr	-0.3	157	-0.1	184

Tbk1	-0.1	178	-0.1	185
Rock1	-0.1	184	-0.1	186
Igf1r	0.0	189	0.0	187
Map2k2	-0.6	122	0.0	188
Pak1	-0.5	142	0.0	189
Sgk3	-0.1	181	0.0	190
Porcn	-0.3	158	0.0	191
Pim2	0.0	190	0.0	192

Table S5. List of antibodies

Antibody	Source	Catalog	Dilution
Zeb1	GeneTex	GTX105278	1:500
E-cadherin	BD Biosciences	610181	1:1000
pCDK4	Abclonal	AP0593	1:500
CDK4	Abcam	ab137675	1:1000
p21	Santa cruz	sc-6246	1:500
pErk	Cell signaling	9101	1:1000
Erk	Cell signaling	4695	1:1000
pRBS780	Cell signaling	8180	1:1000
pRBS807/811	Cell signaling	8516	1:1000
RB	abcam	ab218526	1:1000
pFOXM1	abcam	ab180710	1:1000
FOXM1	abcam	ab207298	1:1000
Actin	ProteinTech	66009-1-Ig	1:10000
Cleaved caspase-3	Cell signaling	9661	1:500
Lamin A/C	Cell signaling	2032	1:1000
Tubulin	Cell signaling	2148	1:1000

Table S6. List of IP reagents

Reagent	Company	Catalog
CDK4 antibody	Santa cruz	sc-56277
p21 antibody	invitrogen	PA1-30399
Clean blot IP reagent	thermofisher	21230
normal mouse IgG	santa cruz	2025
normal rabbit IgG	cell signaling	2729
dynabeads protein a	thermofisher	10001D
dynabeads protein g	thermofisher	10003D

Table S7. List of IHC antibodies

**Antibody**	**Company**	**Catalog**	**Dilution**
Zeb1	Bethyl	IHC-00419	1:200
pRB	Cell signaling	8516	1:100
pCDK4	Invitrogen	PA5-64482	1:250
p21	Novus	NB100-1941	1:200
pErk	Cell signaling	9101	1:250
Ki67	Abcam	ab15580	1:1000

Table S8. List of IF antibodies

Antibody	Company	Catalog	Dilution
pRB	Cell signaling	8516	1:100
CDK4	Abcam	ab137675	1:200
pCDK4	Invitrogen	PA5-64482	1:250
p21	Santa cruz	sc-6246	1:200

Table S9. qPCR primers

Gene name	Primer sequence
ms-CDK4-F	CATACCTGGACAAAGCACCTCC
ms-CDK4-R	GAATGTTCTCTGGCTTCAGGTCC
ms-p21-F	GTGGCCTTGTCGCTGTCTT
ms-p21-R	GCGCTTGGAGTGATAGAAATCTG
ms-p27-F	AGCAGTGTCCAGGGATGAGGAA
ms-p27-R	TTCTTGGGCGTCTGCTCCACAG
ms-Zeb1-F	ATGCTCTGAACGCGCAGC
ms-Zeb1-R	AATCGGCGATCTTTGAGAGCT
hs-p21-F	CAGCAGAGGAAGACCATGTG
hs-p21-R	GGCGTTTGGAGTGGTAGAAA
hs-Zeb1-F	GGCATACACCTACTCAACTACGG
hs-Zeb1-R	TGGGCGGTGTAGAATCAGAGTC

Table S10. List of ChIP reagents

Gene name	Primer sequence
GAPDH-F	AGTGCCAGCCTCGTCCCGTAGACAAAATG
GAPDH-R	AAGTGGGCCCCGGCCTTCTCCAT
mir-200c-F	AGGGCTCACCAGGAAGTGT
mir-200c-R	AGATCCCTGGCTCCCATC
CDKN1A-cp-F	TATTAGCTGGGCATGGTGGT
CDKN1A-cp-R	GCAGCCCTGGCTTTTTGTTT

Reagent	Company	Catalog
ZEB1 antibody	santa cruz	H-102X
normal rabbit IgG	cell signaling	2729
dynabeads protein a	thermofisher	10001D

Table S11. List of shRNA oligos

Gene name
sh-p21-oligo_1.1
sh-p21-oligo_1.2
shCdk4-oligo_1.1
shCdk4-oligo_1.2
shCdk4-oligo_2.1
shCdk4-oligo_2.2

Primer sequence
CCGGGCCCGAGAACGGTGGAACTTTCTCGAGAAAGTTCCACCGTTCTCGGGCTTTTTG
AATTCAAAAAGCCCGAGAACGGTGGAACTTTCTCGAGAAAGTTCCACCGTTCTCGGGC
CCGGGCCCTCAAGAGTGTGAGAGTTCTCGAGAACTCTCACACTCTTGAGGGCTTTTT
AATTAAAAAGCCCTCAAGAGTGTGAGAGTTCTCGAGAACTCTCACACTCTTGAGGGC
CCGGCTGCCGGTTGAGACCATTAAGCTCGAGCTTAATGGTCTCAACCGGCAGTTTTTG
AATTCAAAAACTGCCGGTTGAGACCATTAAGCTCGAGCTTAATGGTCTCAACCGGCAG
